# Cardiovascular Response to *Beta*-Adrenergic Blockade or Activation in 23 Inbred Mouse Strains

**DOI:** 10.1371/journal.pone.0006610

**Published:** 2009-08-12

**Authors:** Corinne Berthonneche, Bastian Peter, Fanny Schüpfer, Pamela Hayoz, Zoltán Kutalik, Hugues Abriel, Thierry Pedrazzini, Jacques S. Beckmann, Sven Bergmann, Fabienne Maurer

**Affiliations:** 1 Service of Medical Genetics, Centre Hospitalier Universitaire Vaudois and University of Lausanne, Lausanne, Switzerland; 2 Department of Medical Genetics, University of Lausanne, Lausanne, Switzerland; 3 Swiss Institute of Bioinformatics, Lausanne, Switzerland; 4 Department of Pharmacology and Service of Cardiology, Centre Hospitalier Universitaire Vaudois and University of Lausanne, Lausanne, Switzerland; 5 Department of Clinical Research, University of Bern, Bern, Switzerland; 6 Department of Medicine, Centre Hospitalier Universitaire Vaudois and University of Lausanne, Lausanne, Switzerland; Duke University, United States of America

## Abstract

We report the characterisation of 27 cardiovascular-related traits in 23 inbred mouse strains. Mice were phenotyped either in response to chronic administration of a single dose of the *β*-adrenergic receptor blocker atenolol or under a low and a high dose of the *β*-agonist isoproterenol and compared to baseline condition. The robustness of our data is supported by high trait heritabilities (typically *H^2^*>0.7) and significant correlations of trait values measured in baseline condition with independent multistrain datasets of the Mouse Phenome Database. We then focused on the drug-, dose-, and strain-specific responses to *β*-stimulation and *β*-blockade of a selection of traits including heart rate, systolic blood pressure, cardiac weight indices, ECG parameters and body weight. Because of the wealth of data accumulated, we applied integrative analyses such as comprehensive bi-clustering to investigate the structure of the response across the different phenotypes, strains and experimental conditions. Information extracted from these analyses is discussed in terms of novelty and biological implications. For example, we observe that traits related to ventricular weight in most strains respond only to the high dose of isoproterenol, while heart rate and atrial weight are already affected by the low dose. Finally, we observe little concordance between strain similarity based on the phenotypes and genotypic relatedness computed from genomic SNP profiles. This indicates that cardiovascular phenotypes are unlikely to segregate according to global phylogeny, but rather be governed by smaller, local differences in the genetic architecture of the various strains.

## Introduction

The *β*-adrenergic system controls cardiac contractility and excitability, heart rate and vascular tone. Pharmacological targeting of *β*-adrenergic receptors is a well documented first-line therapeutic approach for the management of cardiac arrhythmias, cardio-protection after myocardial infarction, and hypertension [1], [2], while *β*-agonists are administered in cases of bradycardia, heart block or asthma [3], [4]. A major concern in clinical practice is the marked variability of human responses to such treatments, often leading to unwanted side-effects or showing at best little relief [5], [6].

The purpose of this study is to explore inter-individual variance of cardiovascular-related traits induced by sustained pharmacological perturbations of the *β*-adrenergic system, using inbred mouse strains as a model. Atenolol (*ate*) and isoproterenol (*iso*) were chosen for their antagonist effects on *β*-adrenergic receptors. *Ate* is a *β*-blocker with strong cardio-selectivity for *β*1-adrenoreceptors. It is widely prescribed to patients with hypertension, coronary heart disease, and arrhythmias. In contrast, *iso* is a classical *β*-adrenergic agonist that induces positive cardiac inotropy and chronotropy. It is administered acutely in cases of bradycardia, heart block or pulmonary emergencies. In both humans and mice, *ate* is mainly eliminated unchanged by the kidneys and the faeces [7], [8], while *iso* is metabolised within minutes post-administration into inactive metabolites by the liver catechol-O-methyltransferase (COMT) [9].

In humans, only about half of hypertensive individuals respond to *ate* by a significant reduction of systolic blood pressure [10], whereas acute delivery of *iso* may induce differential vasodilatory effects in healthy subjects [11]. In rodents and other mammals, sustained activation of the *β*-adrenergic system by chronic administration of *iso* is a classical and well characterised model of left ventricular hypertrophy (LVH) independent of blood pressure [12]. LVH is a major risk factor for ventricular dysfunction and heart failure. In its pathological manifestations, it is characterised by increased left ventricular mass and wall thickness, elevated cross-sectional area and dimensions of cardiomyocytes, as well as increased perivascular and interstitial myocardial fibrosis. LVH is further associated with the reactivation of a foetal transcription program as indicated by up-regulation of cardiac genes otherwise expressed in myocardium only during embryonic development [12]. Recent experimental data suggest that, in mice, *iso*-induced LVH is modulated by strain-specific factors. In particular, extensive phenotypic characterisation revealed that the strain A/J developed greater morphological changes than C57BL/6J when exposed to five consecutive daily injections of 100 mg *iso* per kg body weight [13]. Similarly, evidence obtained in FVB and C57BL6/SV129 mice challenged with single injections of 1 µg *iso* indicates that strain-specific genetic variants are also likely to modulate the chronotropic action of *iso* on heart rate [14].

Here, we report the characterisation of heart rate (HR), systolic blood pressure (SBP), electrocardiograms (ECG) and cardiac weight indices in age-matched males of 23 inbred mouse strains. Rigorous experimental standards were applied so as to minimise the impact of non-inherited factors on trait values and drug responses. In particular, physiological consequences of *β*-adrenergic perturbations were tested independently of pathological influences, measurements were repeated in an average of ten individuals per strain and per drug condition, and sensitive phenotypes such as HR and SBP were monitored across three consecutive days in each mouse, following a training period of one week. These traits were recorded either in baseline condition (*ctr*) or in response to sustained administration of *iso* at 1 (*iso1*) or 10 (*iso10*) mg/kg per day or *ate* at 10 mg/kg per day for two weeks. Mice were chosen in accordance with the recommendations of the Mouse Phenome Database (MPD) so as to cover as much genetic diversity as possible [15], the density of genotypic information available in each strain and their suitability with the experimental design. For these reasons, wild-derived inbred lines were excluded from the panel.

Our study provides a rich dataset of cardiovascular-related phenotypes upon *β*-adrenergic challenge in different genetic backgrounds. We first focus our analysis on the response of key measurements including HR, SBP, ventricular and atrial weight indices, body weight and weight gain, as well as ECG parameters. We then explore the complete dataset using unsupervised bi-clustering to elucidate and visualise the patterns of correlations between strains, treatments, and phenotypes. We compare strain similarity based on the phenotypes with genotypic relatedness computed from genomic SNP profiles. Information extracted from these analyses is discussed in terms of novelty and biological implications. Finally, we discuss the suitability of using our data to map underlying genetic loci by association studies.

## Results

**Figure 1:**
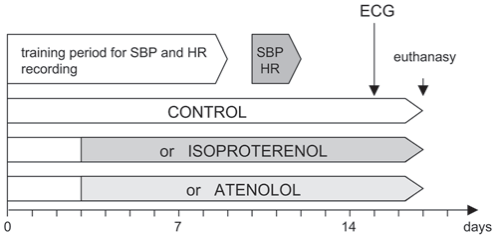
Phenotypic monitoring and timing in inbred mice. Animals were trained on the Visitech BP-2000 tail-cuff apparatus on a daily basis from days 1 to 5 and 8 to 9. SBP and HR were effectively recorded on days 10, 11, and 12. Osmotic mini-pumps loaded with the appropriate drugs were implanted sub-cutaneously on day 3 under anaesthesia. ECGs were recorded on day 15 under halothane anaesthesia. Mice were sacrificed by decapitation on day 17.

**Figure 2:**
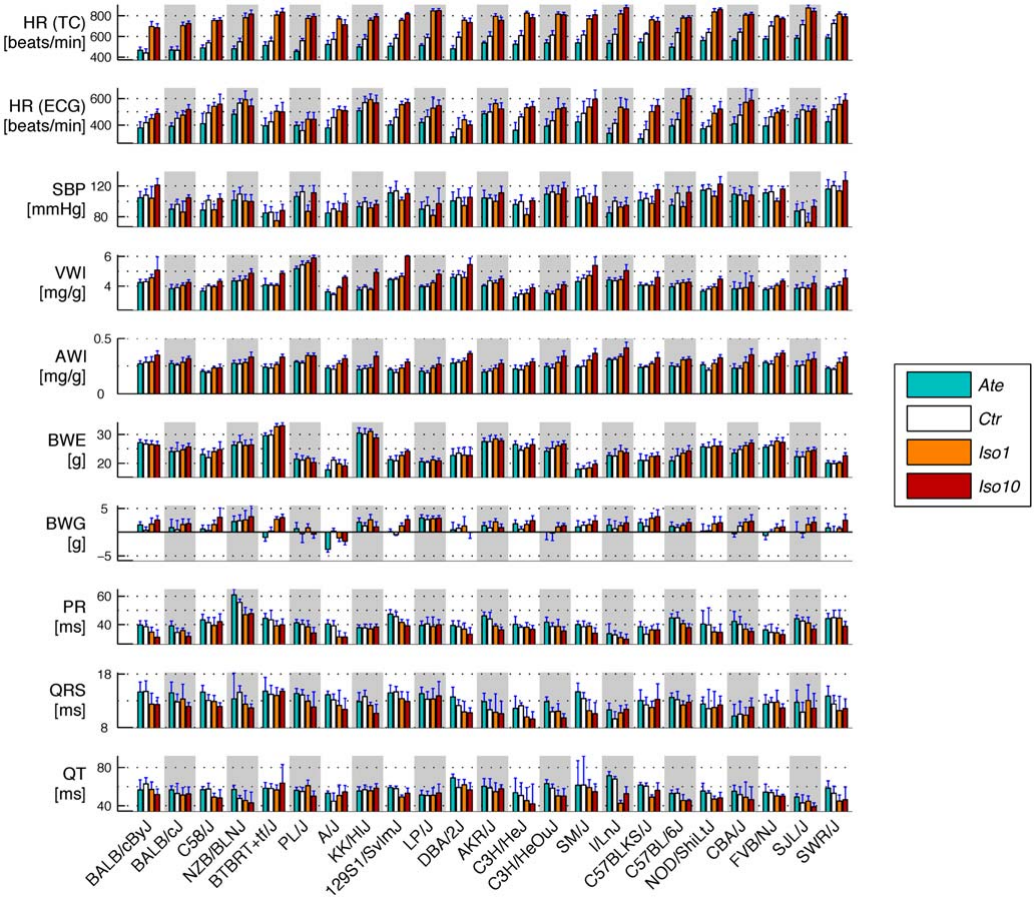
Means and standard deviations of ten selected phenotypes in 23 inbred mouse strains. White bars: ctr; blue bars: ate; orange bars: iso1; red bars: iso10. Strains are ranked by increasing HR (TC) means of ctr mice. See Table 1 for abbreviations.

In this study, hereafter abbreviated CV-PGX (for “cardio-vascular pharmaco-genomics”), we have characterised 23 inbred mouse strains for 27 cardiovascular and related phenotypes such as systolic blood pressure (SBP), heart rate (HR), electrocardiogram (ECG) parameters and cardiac weight indices, either under baseline condition (*ctr*) or in response to chronic administration of isoproterenol at 1 (*iso1*) or 10 (*iso10*) mg/kg per day or atenolol at 10 mg/kg per day (*ate*), as described in **Materials and Methods** and in **Figure 1**. Traits are listed in **Table 1** and mean values and standard deviations of ten selected phenotypes across all strains and drug conditions are presented as bar graphs in **Figure 2** (see **Supplement S1** for all traits). One-way Analyses Of Variance (ANOVA) showed high reproducibility for all phenotypes measured under *ctr* or any given treatment. In particular, variances were significantly smaller within than between strains, indicating that all phenotypic responses are highly heritable (typically 0.7<*H^2^*<1; **Supplement S1**).

**Table 1:** Phenotypes and abbreviations.

condition	abbreviation	phenotype
in conscious mice	HR (TC)	heart rate (tail-cuff; beats/min)
	SBP	systolic blood pressure (tail-cuff; mmHg)
	BWS	body weight at start (g)
	BWE	body weight at end (g)
	BWG	body weight gain (g)
under anaesthesia	HR (ECG)	heart rate (beats/min)
	Pamp	amplitude of p wave (mV)
	Parea	area of p wave (mV*ms)
	Pdur	duration of p wave (ms)
	PR	PR interval (ms)
	RR	RR interval (ms)
	Qamp	amplitude of Q wave (mV)
	QRS	QRS interval (ms)
	QRSarea	area of QRS complex (mV*ms)
	QT	QT interval (ms)
	QTc	corrected QT interval (ms)
	Ramp	amplitude of R wave (mV)
	Samp	amplitude of S wave (mV)
	ST	ST interval (ms)
after euthanasia	HW	heart weight at end (mg)
	VW	weight of cardiac ventricles (mg)
	VWI	ventricular weight index (ratio VW/BWE in mg/g)
	VW/BWS	ventricular weight index (ratio VW/BWS in mg/g)
	AW	weight of cardiac atria (mg)
	AWI	atrial weight index (ratio AW/BWE in mg/g)
	AW/BWS	atrial weight index (ratio AW/BWS in mg/g)
	VW/AW	ratio VW/AW

### Phenotypes measured in baseline condition

**Figure 3:**
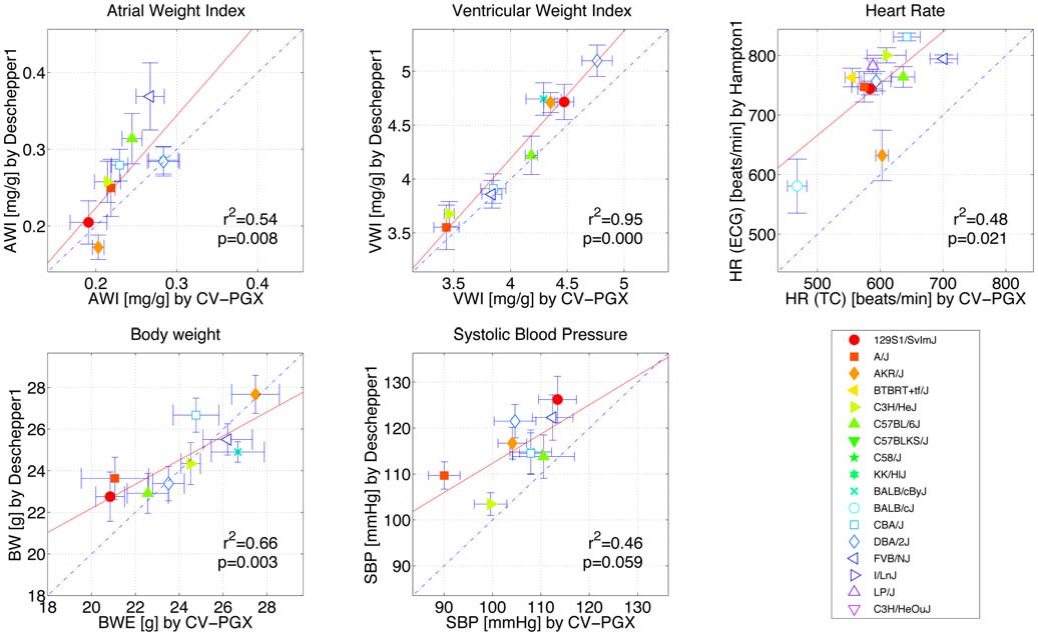
Conservation of baseline phenotypes across independent studies. Five comparisons of ctr CV-PGX strain means with means of selected MPD projects are illustrated. The dashed line is the diagonal of identity and the red line is the best fit of (x∶y) pairs. Data are presented as means±SD. See Table 1 for abbreviations. r2: squared coefficient of correlation (Pearson); p∶ p-value.

We first focused on data recorded in baseline condition. As some of these traits, in particular SBP and HR, are notoriously sensitive to slight perturbations of environmental or experimental conditions, we assessed the consistency of our data with information available from independent experiments and laboratories. To do so, we calculated Pearson's and Spearman's correlations between our mean *ctr* measurements and values from multistrain records of the MPD [15] across the common strains. These comparisons were restricted to MPD projects using inbred strains and experimental procedures similar with those of the present study. A subset of five such pair-wise comparisons is illustrated in **Figure 3**, while more comprehensive results are available in **Supplement S1**.

The most robust phenotypes were those related to body and heart weight, with Pearson's correlations *r^2^*>0.5 (*p*<0.01) in at least one third of the comparisons (**Supplement S1**). This strong conservation is illustrated by the significant correlations of BWE and VWI with the dataset of Deschepper [16], in which ten strains were common with the present study (**Figure 3**). Results were more divergent for the weight of cardiac atria, as average AWI of most strains was lower in the CV-PGX study than in Deschepper's data (**Figure 3**). This discordance is most likely related to the small size of the atria and may be a result of subtle differences in the dissection process rather than intrinsic strain variability.

The conservation of baseline HR and SBP strain means across independent studies was much weaker, as illustrated by the two examples in **Figure 3**. Yet, the total numbers of significantly correlated CV-PGX *vs* MPD datasets were higher than expected by chance for both traits (**Supplement S1**), suggesting that despite marked susceptibility to the environment, blood pressure and heart rate are also controlled in part by genetic determinants. Altogether, these comparisons indicate that *ctr* measurements are consistent with data from other studies, further supporting the validity of our experimental protocol.

Of note, mean HR values measured by ECG correlated poorly with those obtained (i) by tail-cuff in the same animals (Pearson *r* ^2^ = 0.02, *p = *0.52; **Supplement S1**) or (ii) by ECGs in independent studies (MPD). Considering that tail-cuff experiments were performed in conscious mice whereas ECGs were recorded under anaesthesia, these discrepancies may point towards strain-specific confounding effects of anaesthesia.

### Patterns of trait and strain correlations in baseline condition

Baseline phenotypes were further investigated for patterns of correlations across strains. To this end we normalised phenotypic data into *z*-scores and analysed them by hierarchical bi-clustering (**Supplement S1**). In these re-ordered tables (matrices) of *z*-scores, phenotypes (columns) were clustered according to the absolute values of the similarities across all strains while strains (rows) were clustered according to signed similarities across all phenotypes.

The vast majority of the *ctr* phenotypes were only mildly correlated across strains (correlations *r*<0.5, **Suppl.** **Figure 5A** in **Supplement S1**) but as expected, HW, AW and VW increased with BWE (correlations *r*>0.7, *p*<0.05). Interestingly, these traits also shared good similarity with Pdur (0.4<*r*<0.7, *p*<0.05). In contrast, no significant correlation could be identified between HR and SBP (*r* = 0.25, *p* = 0.26), between HR and VWI (*r* = −0.21, *p* = 0.34) or AWI (*r* = −0.12, *p* = 0.59), and between SBP and AWI (*r* = 0.04, *p* = 0.87) or VWI (*r* = 0.27, *p* = 0.22). In anaesthetised mice, ECG intervals were only mildly correlated to HR. Altogether these data suggest that the genetic components contributing to most baseline phenotypes are largely independent.

**Figure 4:**
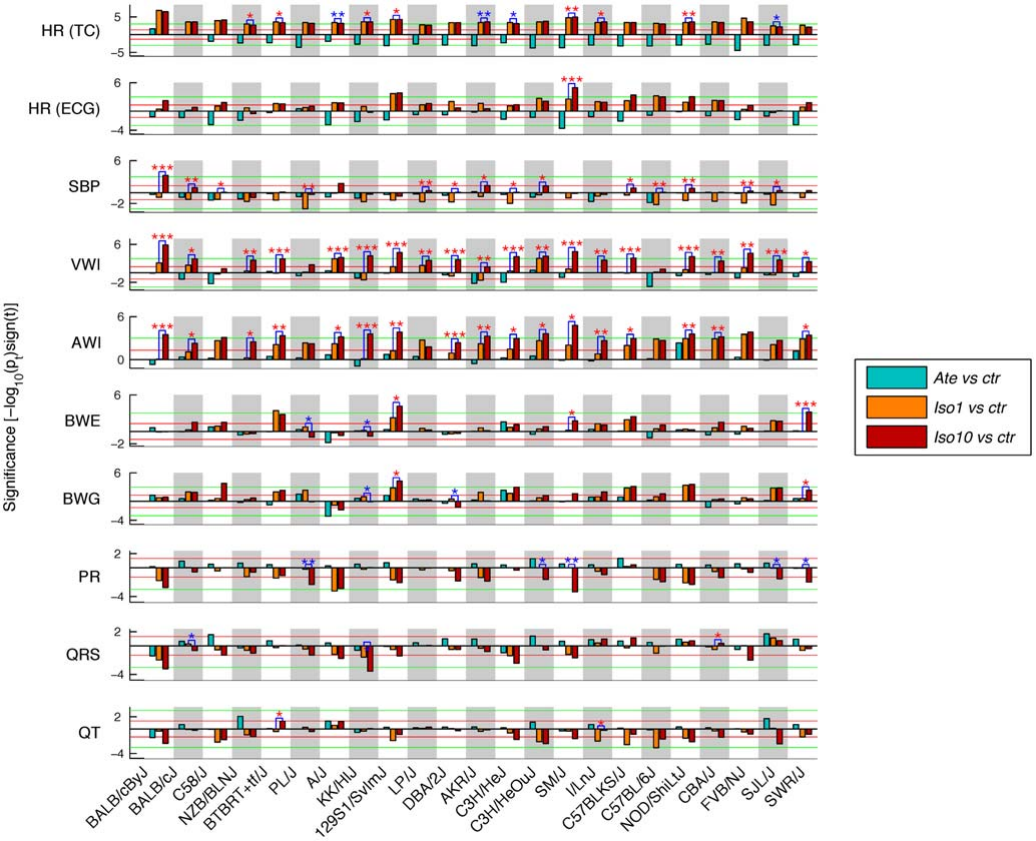
Intra-strain significance of drug treatments. Data are presented for the phenotypes selected in Figure 2. Intra-strain p-values of phenotypic data recorded in treated vs untreated groups (Wilcoxon ranksum statistics) are presented as bar graphs on a -log10 scale. The threshold of significance is indicated by red lines (p = 0.05) and -log p-values are signed according to the directionality of the effect induced by the drugs. When significant, p-values for testing responses under iso10 vs iso1 are indicated by coloured stars (i.e. *: p<0.05; **: p<0.01; ***: p<0.001; red star: phenotypic mean under iso10> phenotypic mean under iso1; blue star: phenotypic mean under iso10<phenotypic mean under iso1). Blue bars: ate vs ctr; orange bars: iso1 vs ctr; red bars: iso10 vs ctr. P-values smaller than 0.001 (bars extending beyond the green lines or ***) hold up against Bonferroni correction for multiple testing of either all strains for a given phenotype or all phenotypes for a given strain. Strains are ranked as in Figure 2. See Table 1 for abbreviations.

### Phenotypes measured under atenolol or isoproterenol treatment

We next focused on strain responses to chronic *β*-adrenergic blockade or activation. For each phenotype *p* and strain *s* a signed significance value
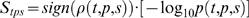
was attributed to the effect of each treatment *t = ate*, *iso1*, *iso10* with respect to the *ctr* group. We used the Wilcoxon ranksum test, with test statistics 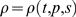 and associated *p*-value 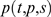. Signed significances are presented in **Figure 4** for the same parameters and strain order as in **Figure 2** (graphs for all phenotypes are available in **Supplement S1**). Altogether, the amplitudes of responses to drug treatments were strain-specific and high- or low-responders could be identified for each trait. Most phenotypes were affected by (at least the highest dose of) *iso* with a nominal significance level of *p*<0.05 in at least one strain, whereas the opposite effect of *ate* was usually milder. Below, we focus on the results of HR, SBP and cardiac weight indices. Information regarding the effect of *ate* and *iso* on ECG parameters and body weight are available in **Supplement S1**.

**Figure 5:**
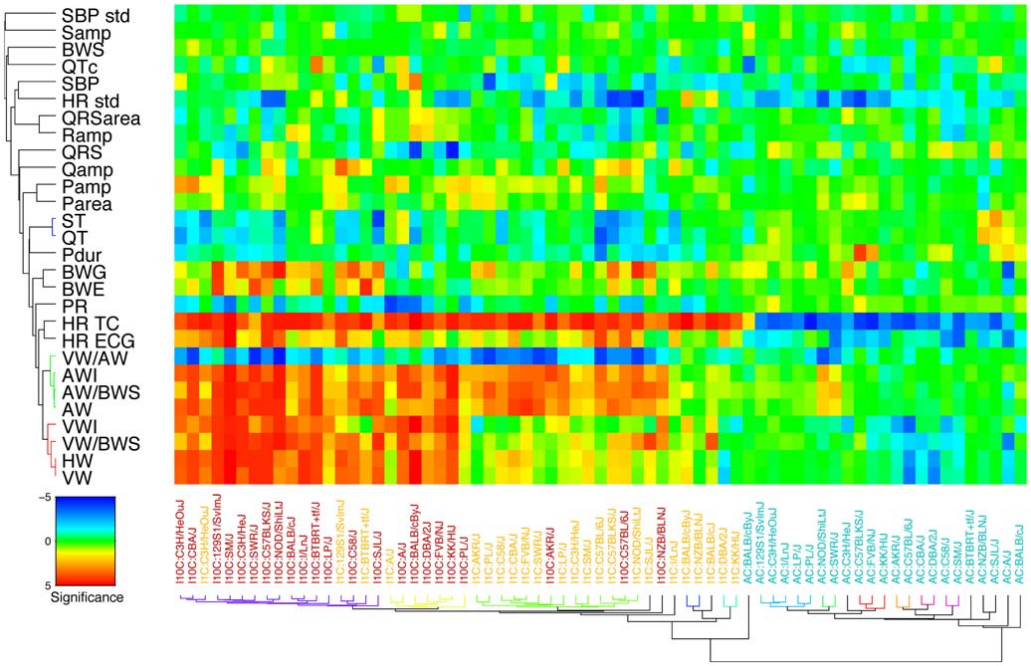
Patterns of phenotype and strain correlations under drug treatment. For each phenotype (rows) and combination (columns) of a strain and a treatment (ate, iso1, iso10) the significance (signed -log10 value of Wilcoxon ranksum test, as used in Figure 4) of the phenotypic response with respect to the ctr group is shown using a colour code. Rows and columns are clustered according to pattern similarity. The branches of the dendrograms illustrating the clusters are plotted with the same colour as long as the average linkage distance is less than 20% of the maximal distance. AC: ate vs ctr; I1C: iso1 vs ctr; I10C: iso10 vs ctr. See Table 1 for abbreviations. HR std: standard deviation of HR (TC) strain means; SBP std: standard deviation of SBP strain means.

We note that the significance levels in **Figure 4** (and **Suppl.** **Figure 4** of **Supplement S1**) have not been corrected for multiple testing. When asking whether a response is significant in the global context of our analysis, the significance threshold has to be adjusted. A simple procedure is the well-known Bonferroni correction, where the nominal significance threshold α = 0.05 is lowered to α' = α/*N* (*N* being the number of tests). For example, for a given phenotype (or strain) one corrects for considering simultaneously *N* = 23 (*N* = 27) tests, giving α'≈0.002. Thus significance values extending beyond the (green) threshold lines at±3 in **Figure 4** clearly hold up against testing all strains *or* all phenotypes. Yet, this correction is very stringent and over-conservative – in particular when correcting for testing all phenotypes *and* all strains. Thus we also applied the Benjamini-Hochberg (BH) step-up procedure [17], [18] to correct for multiple hypotheses testing. This procedure controls the false discovery rate yielding adequate, yet significantly milder corrections. We report globally BH-corrected *p*-values as well as those for any given phenotype or any given strain (correcting for all 23 strains or all 27 phenotypes, respectively) in **Supplement S1**.

### Heart rate in conscious mice

The most robust effect of the *β*-adrenergic drugs was the pronounced strain-specific positive cardiac chronotropy induced by both concentrations of *iso* in all 23 strains (**Figures 2** and **4**). Under *iso10* stimulation, mean HR was increased between 61±43 beats/min in strain SWR/J and 279±33 beats/min in strain BTBRT^+^ *tf*/J. The lower concentration of *iso* induced a similar or even slightly higher (*i.e.* see strains A/J, AKR/J, C3H/HeJ and SJL/J) acceleration in sixteen strains, while for the seven other lines HR was significantly higher under *iso10* than *iso1* (**Figures 2** and **4**). Conversely, the opposite properties of *ate* on HR were significant in all strains except both Balb/c lines, reaching a maximal reduction of 141±43 beats/min in strain SWR/J.

Overall, there was a marked tendency that stronger responders to *β*-stimulation had lower baseline HR, while stronger responders to *β*-blockade had higher *ctr* HR. In other words in most strains baseline values could be used to predict the magnitude of rate changes in response to pharmacological treatment. These data may reflect the existence of electrical and/or mechanical limits to the pacing capacity of the atrial sinus node. Reaching the upper limit of cardiac pacing would explain the relatively weak *iso*-mediated positive chronotropy in strains SJL/J and SWR/J, whose pulse was initially high. Accordingly, strain upper limits were attained in at least sixteen lines exposed to *iso* (*i*.*e*. those in which pulse recorded under *iso1* was higher or similar as under *iso10*), ranging from 697±45 beats/min in strain Balb/cByJ to 877±45 beats/min in strain I/LnJ. It is more difficult to draw conclusions regarding the lower limit of pacing automaticity, since a single dose of *β*-blocker was used. Yet, HR mean values measured in conscious *ate*-treated mice were never lower than the baseline pulse values of strain Balb/cByJ (*i*.*e*. 442±35 beats/min).

*r* ^2^ values of Pearson's correlations between pulse rate recorded in resting state or under drug treatment were 0.74 for *ate vs ctr* (*p* = 1.4×10^−7^), 0.54 for *iso1 vs ctr* (*p* = 7.3×10^−5^), and 0.22 for *iso10 vs ctr* (*p* = 2.5×10^−3^; **Suppl.** **Figure 3** of **Supplement S1**). This information is consistent with *iso10* producing the strongest perturbations on HR and indicates that the patterns of drug- and dose-dependent HR responses are strain-specific. Similarly, when assessing the impact of the treatments on the correlations with the datasets of the MPD (**Supplement S1**), Pearson's *r* ^2^ values decreased when comparing pulse of drug-treated animals instead of our *ctr* with that of Hampton1's records [19]. When considering the other MPD projects, correlations were the highest in (CV-PGX*_ate_*:Svenson), (CV-PGX*_iso10_*:Gavras), and (CV-PGX*_iso10_*:Jaxwest1_HR_) pairs (**Supplement S1**), probably reflecting differences in stress levels across projects.

#### Systolic blood pressure

Perturbations of the *β*-adrenergic system resulted in rather subtle effects on blood pressure. *Ate* tended to slightly decrease SBP, but the effect was below significance in all strains except C57BL/6J (**Figure 4**). *Iso1* also reduced SBP, on average by 10 mmHg, probably as a result of vasodilation. This decrease reached significance in five strains (C3H/HeJ, C57BL/6J, FVB/NJ, PL/J, and SJL/J). As for *iso10*, it tended to slightly increase SBP in the majority of the strains, perhaps as a consequence of increased cardiac inotropy, but the trend was significant in a single line (*i*.*e*. Balb/cByJ). Even though differences between *iso*-treated and *ctr* mice were globally minor, the opposite action of *iso1* and *iso10* on SBP was significant in fourteen strains (**Figure 4**). Modest hemodynamic changes in response to *iso* are consistent with previous data obtained in rats [20] and mice [13], confirming that cardiac hypertrophy induced by *iso* is essentially independent of SBP.

#### Heart weight and cardiac weight indices

Chronic activation of the *β*-adrenergic system is a classical trigger of left ventricular hypertrophy [12], [21]. In our study, *iso* induced strain-, dose- and compartment-specific perturbations of cardiac mass (**Figures 2**, **4** and **Supplement S1**). Under infusion of *iso10*, the relative increase of VWI reached significance (*p*<0.05) in nineteen strains, ranging from 1.7% in C57BL/6J mice to 33% in 129S1/SvImJ (*i*.*e*. C57BL6/J: 4.3±0.03 mg/g in *iso10 vs* 4.2±0.04 mg/g in *ctr*; 129S1/SvImJ: 6.0±0.26 mg/g in *iso10 vs* 4.9±0.05 mg/g in *ctr*). By comparison, only three lines differed from the controls in the *iso1*-treated group, reaching a maximal relative increase of 11% in strain A/J (**Figure 4**). These three strains were all characterised by a positive dose-response to *iso*. In contrast to the above, the resistance of strains AKR/J, C57BL/6J, and PL/J to *β*-stimulation was characterised by similar indexed and non-indexed ventricular weight in treated and untreated mice (**Figures 2** and **4**, and data not shown), whereas the small variation of VWI in C58/J mice was the consequence of parallel increases of VW and BW.

Cardiac atria responded to chronic *β*-stimulation with the same directionality as the ventricles but exhibited more pronounced sensitivities to *iso* (**Figures 2** and **4**): AWI was increased by *iso1* in fifteen strains, including those with poor responding ventricles, and by *iso10* in all lines (*p*<0.05). The magnitude of atrial hypertrophy was larger under *iso10*, extending from a relative increase of AWI of 22% in strain Balb/cByJ to 57% in CBA/J.

Consistent with the fact that *ate* was administered to healthy mice, *β*-blockade only modestly affected cardiac weight and indices in the majority of the strains. Three of the four lines resistant to *iso* responded by a significant reduction of VWI under *ate* (*i*.*e*. AKR/J: −6.4%; C58/J: −7.8%; and C57BL/6J: −5.6%, *p*<0.05), but a concomitant reduction of VW was significant only in C57BL/6J mice (data not shown). Similarly, the overall impact of *ate* on cardiac atria was negligible except in strain NOD/ShiLtJ that exhibited a relative increase of AW and AWI of 21% when compared to controls.

Of note, the weak *iso*-dependent increase of VWI in strain C57BL/6J is consistent with the data of Faulx *et al*. who investigated cardiovascular changes in 12–15 week-old A/J and C57BL/6J males challenged with five consecutive daily injections of 100 mg/kg *iso* [22]. In these conditions, the heart weight index (HWI) increased by 2.7% in strain C57BL/6J and by 23% in strain A/J. In contrast, HWI in 8-wk old C57BL/6J males infused with 40 mg/kg *iso* for seven days increased from 5.0 in *ctr* to 6.0 mg/g (*i*.*e*. a relative increase of 22%) in treated animals [23]. These apparent inconsistencies may relate to the fast biotransformation of *iso* by liver enzymes [9]. In the latter example, the dose of continuously-administered *iso* was indeed four-fold higher than the CV-PGX *iso10* concentration. While the 100 mg/kg used by Faulx *et al*. might seem even higher, one should keep in mind that in this case *iso* was administered in single daily shots. Thus, the total daily amount of drug is likely to be much lower than when delivered in a sustained fashion as most of it would have been rapidly metabolised and eliminated.

#### Correlations and clustering of phenotypes in the presence of β-adrenergic drugs

Each drug treatment was assessed for its impact on the patterns of correlations across mean trait values or strains (see **Suppl.** **Figures 5** **–** [Fig pone-0006610-g006] **7** in **Supplement S1**). The high correlations between HW, AW, VW and BWE were globally maintained also upon perturbations of the *β*-adrenergic system but were slightly reduced under *iso10* as compared to *ctr* mice (*i*.*e*. 0.4<*r*<0.8; compare **Suppl.** **Figures 6A and 6D** in **Supplement S1**). High similarity of HW and VW with Pdur was still detected under *β*-stimulation (*i*.*e*. 0.5<*r*<0.8) but it was significantly reduced under *ate* (*r* = 0.19, *p* = 0.38). Again and irrespective of the drug condition, no significant correlation could be identified between HR and SBP, between HR and VWI or AWI, and between SBP and AWI or VWI.

**Figure 6 pone-0006610-g006:**
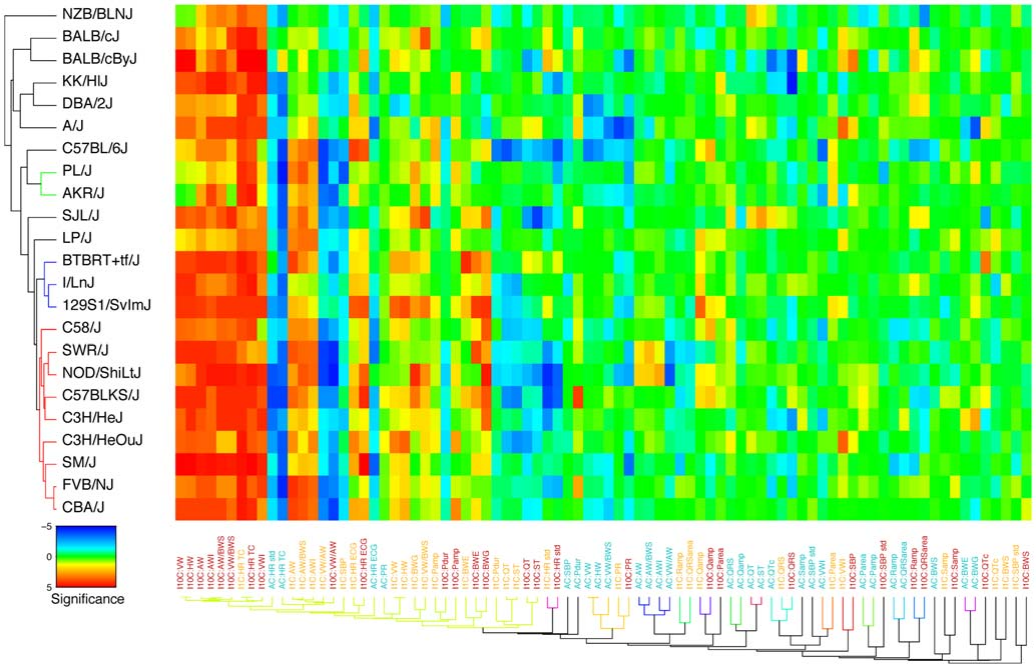
Patterns of phenotype and strain correlations under drug treatment. For each strain (rows) and combination (columns) of a phenotype and a treatment (ate, iso1, iso10) the significance (signed -log10 value of Wilcoxon ranksum test, as used in Figure 4) of the phenotypic response with respect to the ctr group is shown using a colour code. Rows and columns are clustered according to pattern similarity. The branches of the dendrograms illustrating the clusters are plotted with the same colour as long as the average linkage distance is less than 50% of the maximal distance. AC: ate vs ctr; I1C: iso1 vs ctr; I10C: iso10 vs ctr. See Table 1 for abbreviations. HR std: standard deviation of HR (TC) strain means; SBP std: standard deviation of SBP strain means.

**Figure 7:**
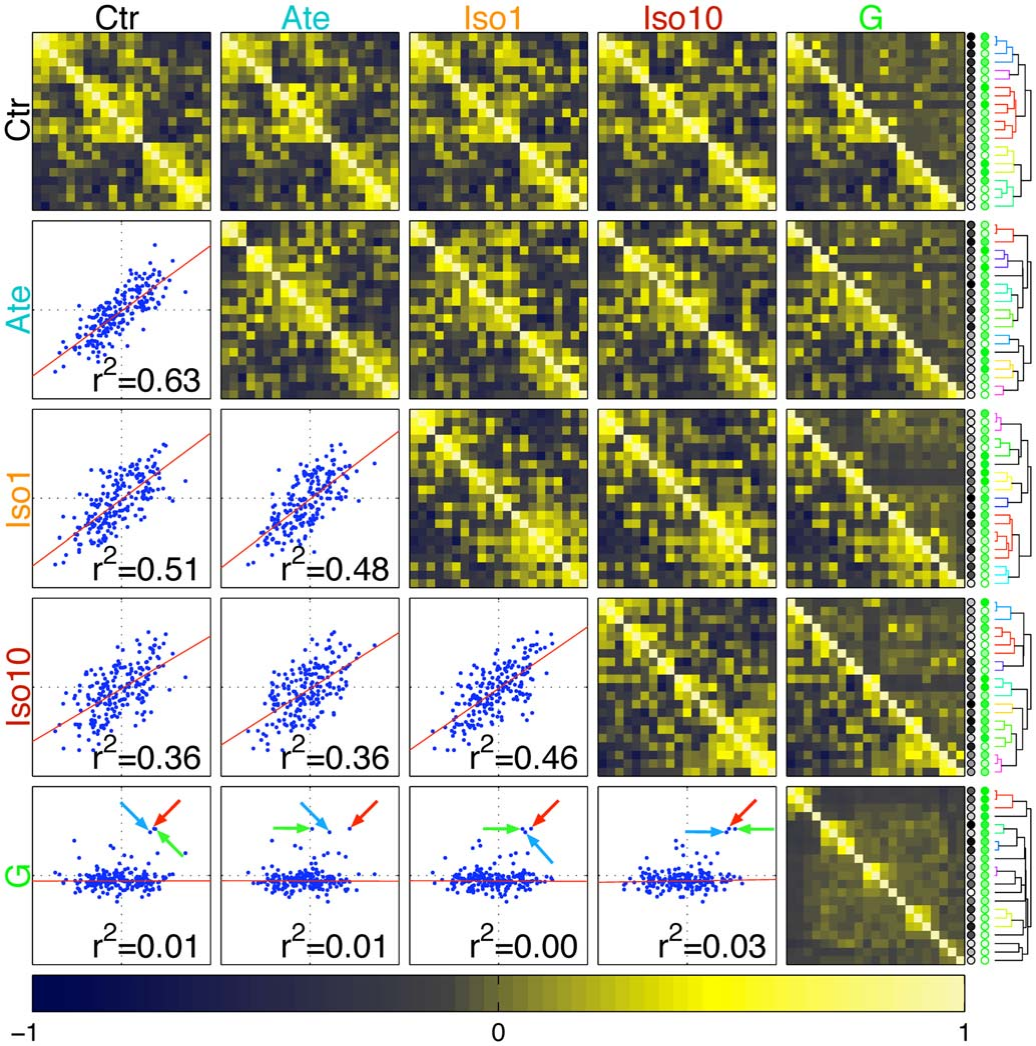
Comparison of strain relatedness based on phenotypes or genotypes. Along the main diagonal, five clustered correlation matrices 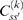 (where k = ctr, ate, iso1, iso10, or G) across all phenoypes or SNPs (k = G) are shown. Similar strains are placed close to each other and their order is indicated by the coloured circles and dendrograms displayed on the right as determined by standard hierarchical clustering. Above these five matrices ten composite matrices are shown whose lower left part consists of the correlation matrix shown on the main diagonal to the left, while their upper right contains the correlations from the dataset indicated on top, using the same order of strains (i.e. the rows of all adjacent correlation matrices correspond to a fixed strain). The degrees of correlations are indicated by a colour code. In the scatter plots below the diagonal, each pair of strains (s,s') is represented by a dot whose coordinates are given by the correlations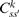 and 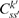 where k and k' are indicated on the left and top, respectively. The correlation r2 between these dots is indicated and a red line shows the best linear fit. Blue arrow: C3H/HeJ vs CBA/J, red arrow: 129S1/SvImJ vs LP/J, green arrow: C57BL76J vs C57BLKS/J.

### Structure of the response to *β*-adrenergic drugs

In the previous paragraphs, we focused on the differential behaviour of a selection of traits across strains and treatments, considering one trait and/or one drug condition at a time. Yet, such serial screening is not well suited to fully exploit the rather large set of phenotypic data we generated (*i*.*e*. 27 phenotypes measured in 23 strains and under 4 treatment conditions). In order to explore the relationships between the various parameters in a more comprehensive manner and to provide a more global picture on the structure of these data, we performed unsupervised bi-clustering analyses. Specifically, we transformed the three dimensional table 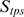 of the signed *p*-values attributed to the effect of each treatment (*t = ate*, *iso1*, *iso10*) with respect to the *ctr* group into two two-dimensional tables (matrices). The first matrix *S_pi_* (**Figure 5**) has one row for each phenotype *p*, while each column *i* refers to a combination (*t,s*) of a treatment *t* in strain *s*. Conversely, the second matrix *S_sj_* (**Figure 6**) has one row for each strain *s*, while each column *j* refers to a combination (*t,p*) of a treatment under which the phenotype *p* was observed (we chose not to consider a matrix that has one row for each treatment). Hierarchical bi-clustering was applied to determine clusters in these matrices (see **Materials and Methods** for details). Strains were clustered according to (signed) values of similarity (*i*.*e*. Pearson correlations between significance profiles across all phenotypes). In contrast, we used absolute correlations as similarity measure between phenotypes (such that inversely related observables, like HR and ECG intervals are clustered together). The reordered matrices together with the similarity dendrograms are shown in colour codes, providing a convenient, unbiased and condensed means to compare subsets (clusters) of related phenotypes or strains based on their response to *β*-adrenergic drugs.

In **Figure 5**, strain responses to pharmacological challenge (*i.e*. “treated strains”) clustered mainly according to the type of drug, as the first branching in the tree clearly discriminates *ate*- from *iso*-treated lines (with the exception of strain Balb/cByJ under *ate*). This segregation reflects the opposite response of HR to the two antagonist drugs, indicating that HR reaction to treatments was the most discriminative phenotype of the whole dataset (according to principle component analysis, data not shown). Sub-clustering in *iso*-mediated responses was essentially driven by the strain-specific and dose-dependent responses of cardiac ventricles and atria to *β*-stimulation. This resulted in an almost perfect segregation between the *iso1*- and *iso10*-treated groups of strains. Five strains were poor responders to *iso1* at the level of cardiac atria (when compared to controls) and segregated in three separate clusters (*i*.*e*. strains KK/HlJ, DBA/2J, Balb/cJ, NZB/BlNJ, and Balb/cByJ). Of all strains exhibiting increased AW and AWI under *iso*, a subset of twelve lines exhibited modest changes of HW, VW and VWI (*i*.*e*. see green cluster of treated strains in **Figure 5**). This effect was present under *iso1* in ten strains (*i*.*e*. strains NOD/ShiLtJ, C57BLKS/J, SM/J, C3H/HeJ, LP/J, SWR/J, FVB/NJ, CBA/J, C58/J, and PL/J) and under both concentrations of *iso* in lines AKR/J and C57BL/6J. The main trends in the remaining strains were essentially (but not exclusively) observed under *iso10*. They were dominated by concomitant increases of AW, AWI, HW, VW, and VWI. These strong responding strains clustered into two distinct subsets, depending on the effect of sustained *β*-stimulation on BW and BWG. Thus, both values remained unchanged in six lines (*i*.*e*. PL/J, KK/HlJ, FVB/NJ, DBA/2J, Balb/cByJ, and A/J), while they tended to increase in the majority of the fourteen others (*i.e.* see purple cluster of treated strains in **Figure 5**). While strains of the green sub-cluster tended to exhibit reduced blood pressure under treatment, this trait was either stable or slightly increased in the yellow and purple groups.

Looking at the categorisation under *β*-blockade, we first note that both Balb/c strains were relatively insensitive to *ate*, in particular for HR. As such, they did not co-segregate with any of the other lines. The next most informative sub-category correlates best with HR patterns of the ECG data, mirroring differential pulse responses under anaesthesia.

From the same figure, it is also possible to address the segregation of phenotypic modifications across all treatments. More specifically, the first two clusters (*i.e.* red and green clusters of phenotypic changes, **Figure 5**) include responses of traits related to cardiac weight and indices (correlations across trait responses: 0.5<|*r*|<1, **Suppl. Figure 8A**, **Supplement S1**). The structure of these clusters was essentially determined by the differential (and dose-dependent) effects of *iso* on cardiac ventricles and atria. These responses are in relatively close proximity with those of HR (absolute correlations: 0.5<|*r*|<0.9), PR (absolute correlations: 0.6<|*r*|<0.8), BWE (correlations: 0.4<*r*<0.8) and BWG (correlations: 0.4<*r*<0.8), whereas the patterns of SBP variations are more discordant (absolute correlations: |*r*|<0.4). Interestingly, despite the effect of anaesthesia on ECG parameters, significance scores for the two measurements of HR responses are highly correlated across all treated strains (*r* = 0.79, *p* = 3×10^−7^, **Suppl. Figure 8A**, **Supplement S1**), even though the respective baseline values were only poorly correlated. This is reflected by HR and HR (ECG) appearing co-clustered, while the patterns of PR intervals modifications are slightly more distinct (correlation with HR: *r* = −0.67, *p* = 7×10^−5^). Control phenotypes such as body weight before treatment (BWS) and SBP standard deviation (SBP std) that did not notably vary across strains and drug conditions did not cluster with the other variables. Apart from Pamp, amplitudes and areas of ECG waves, which were poorly affected by the drugs, are also relatively distant from the other traits.

**Figure 6** summarises the clustering of inbred strains as determined by their responses to *ate* and *iso*. As expected, strains were all positively correlated (*i.e*. correlations: 0.4<*r*<1, **Suppl.** **Figure 9A**, **Supplement S1**), indicating that they usually differed only in the relative amplitude but not the directionality of the responses. NZB/BlNJ mice were on average the least affected by the treatments and as such the most distant from the others. Three sub-groups seem to emerge from this analysis. The first one comprises fourteen strains organised in two clusters and two separate lines (*i*.*e*. red cluster: strains CBA/J, FVB/NJ, SM/J, C3H/HeOuJ, C3H/HeJ, C57BLKS/J, NOD/ShiLtJ, SWR/J, and C58/J; blue cluster: strains 129S1/SvImJ, I/LnJ, and BTBRT^+^ *tf*/J; as well as strains LP/J and SJL/J, **Figure 6**; correlations: 0.7<*r*<1, **Suppl. Figure 9A**, **Supplement S1**) while the second category includes five strains (*i*.*e*. A/J, DBA/2J, KK/HlJ, BALB/cByJ, and BALB/cJ; correlations: 0.6<*r*<0.9). Three additional strains (*i*.*e*. AKR/J, PL/J and C57BL/6J) are in mild proximity with the first sub-group (*i*.*e*. correlations: 0.6<*r*<0.9) and more distant from the second one (*i*.*e*. correlations: 0.4<*r*<0.8). Trends specific to the first sub-group are marked increases of (i) HR (ECG) under *iso*, (ii) AW, AWI, VW, VWI, and HW under *iso10*, and (iii) BWG under *iso10*. The second cluster is characterised by very mild changes of (i) HR under anaesthesia, (ii) AW and AWI under *iso1* and (iii) BWG under *iso10*, whereas HW, VW, and VWI are significantly increased by *iso10*. The three strains standing between these two groups correlate with very mild variations of VW, HW, and VWI under both concentrations of *iso* as well as decreased SBP under *iso1*. It is tempting to speculate that these phenotypic patterns segregate due to specific genetic determinants but further analyses will be required to establish our observations and link them to genetic variation.

On the other axis of **Figure 6**, phenotype treatment combinations exhibit no clear clustering hierarchy (except for small groups of trivially related phenotypes). Rather weakly affected traits in response to *β*-adrenergic perturbations gradually agglomerate towards the right, the more strongly affected traits appearing on the left.

### Strain relatedness based on phenotypic or genotypic data

Of the 23 strains characterised above, 21 have been genotyped at a genome-wide density of over 100′000 SNPs [24]. To compare strain proximity based either on the phenotypes observed in each of the four experimental conditions (*ctr*, *ate*, *iso1* and *iso10*; **Suppl.** **Figures 5** **and** **7** in **Supplement S1**) or on these genotypes (*G*; **Suppl. Figure 10** in **Supplement S1**), we computed the five corresponding correlations matrices 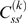 (where *k* = *ctr*, *ate*, *iso1*, *iso10*, or *G*, see **Materials and Methods** for details). In **Figure 7**, these (symmetric) matrices are drawn along the main diagonal of the figure (*i.e.* from top left to bottom right) with similar strains placed close to each other (*i.e.* the highest inter-strain correlations tend to appear close to the diagonal of each matrix). Strain order was determined by standard hierarchical clustering and is indicated by the circles and dendrograms displayed on the right of the figure. Specifically, the order based on phenotypic (*ctr*) relatedness is symbolised by shades inside the circles graded from black to white, whereas the order based on genetic proximity is represented by shades graded from green to white (*i*.*e*. each circle represents a single strain and strains with similar shades tend to be closely related). Dendrograms associated to trait-based relatedness are identical to those of **Suppl.** **Figures 5** and **7** (**Supplement S1**), with the exception that strains Balb/cJ and C3H/HeOuJ, for which genetic information was missing [24], were not taken into account. Above these five matrices we show ten composite matrices. In each of the latter, the lower left part is identical to the correlation matrix shown on the main diagonal on the left, while the upper right contains the correlations within the datasets indicated on top (while keeping the same strain order as in the lower left part). In other words, the matrices in the top row of the figure allow for the successive comparison of the strain proximity as determined by *ctr* phenotypes with those obtained from traits recorded under *ate*, *iso1* and *iso10* or from the genotypes. Accordingly, the second row allows for comparison across treatments or genotypes using the strain order as determined by *ate* phenotypes, and so on. Below the main diagonal we show scatter plots, where for each pair of strains (*s,s*') we draw a dot whose coordinates are given by the correlations 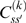 and 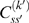 where *k* and *k*' are indicated on the left and top, respectively. The correlation *r^2^* between these dots is indicated and a red line shows the best linear fit of the data.

Pair-wise comparisons indicate that the patterns of strain relatedness based on any of the four sets of phenotypic data are positively correlated. The closest profiles are those obtained from traits measured in *ctr* and *ate* mice (*i.e*. correlation *r* ^2^ = 0.63, *p* = 7×10^−7^), while those resulting from the phenotypes measured in *ctr* and *iso10* or *ate* and *iso10* are significantly more divergent (*i.e*. *r* ^2^ = 0.36, *p* = 0.001 and *r* ^2^ = 0.36, *p* = 0.001, respectively, **Figure 7**). These differences reflect the strain-specificity of the trait modifications induced by the *β*-adrenergic drugs, in particular *iso10*.

Nominal values of inter-strain correlations based on whole-genome genetic profiles are detailed in **Suppl. Figure 10** (**Supplement S1**). In this analysis, the vast majority of the correlations are below 0.2. Four non-overlapping clusters of strains are characterised by correlations above this value, the closest pairs being 129S1/SvImJ *vs* LP/J, C3H/HeJ *vs* CBA/J, and C57BL/6J *vs* C57BLKS/J (*i*.*e*. all with correlations *r*>0.4). This hierarchy agrees well with the known ancestry of laboratory mouse strains [25], [26]. Irrespective of the drug condition, we observe little concordance when comparing overall strain phylogeny based on phenotypes with relatedness computed from the genomic SNP-profiles (**Figure 7**). Yet, it is interesting to note that the three genetically closest pairs of strains tend to segregate in the same or adjacent trait-based clusters (see arrows in **Figure 7** and related clusters in **Suppl.** **Figure 7** of **Supplement S1**). This is true for strains 129S1/SvImJ and LP/J in all conditions and for strains CBA/J and C3H/HeJ as well as C57BL/6J and C57BLKS/J in *ctr*, *iso1*, and *iso10* conditions. Conversely, we have already highlighted divergence between strains C57BL/6J and A/J or C57BL/6J and 129S1/SvImJ for traits such as VWI under *β*-stimulation. Accordingly, when considering all phenotypes, trait-based relatedness is consistently relatively low between these latter pairs of strains (**Suppl.** **Figure 7** in **Supplement S1**). Nevertheless, globally the correlation between relatedness based on our cardiovascular phenotypes and whole-genome SNP profiles is low. This suggests that these phenotypes are likely to be governed by smaller, local differences in the genomes of the various strains.

## Discussion

Inter-individual differences in response to medication are well-established clinical issues, the study of which is a large field of research in itself. Yet, pharmacogenetic studies in humans are hampered by several important concerns such as compliance issues, environmental confounders and limitations to non-invasive measurements. In this context laboratory mice, which have already played a key role for dissecting the genetic basis of disease susceptibility and providing key support for many current concepts of disease pathogenesis and treatment strategies, are also likely to become instrumental for complementing and furthering human pharmacogenetics [27]. Indeed, several recent studies already addressed the genetic basis of strain-specific susceptibility to carcinogens [28], [29] and analgesics [30], [31] or the metabolism of warfarin, testosterone and irinotecan [32], [33] in inbred mice.

Here we undertook a similar approach focusing on the cardiovascular system. Our large survey assessed the suitability of using a large panel of inbred mouse strains to investigate physiological responses to *β*-adrenergic treatments. To the best of our knowledge, it provides the first large-scale standardised characterisation of heart rate, systolic blood pressure, ECG and cardiac weight indices in response to sustained infusion of atenolol and isoproterenol in such a panel.

The robustness of our experimental standards is supported by several essential validation steps: first, mean strain values measured in *ctr* condition correlated well with independent datasets of the Mouse Phenome Database. In agreement with the notion that morphological parameters are highly heritable and least affected by environment, traits related to body and cardiac weight were the most consistent with independent studies. Even though conservation across independent datasets was much reduced for SBP and HR, the number of significant between-projects correlations was higher than expected by chance for both traits. As discussed by others [16], [34], [35], this confirms that despite marked susceptibility to environmental factors, a significant portion of HR and SBP variance observed in inbred mouse strains is genetically determined. Second, phenotypic variances were generally smaller within than between strains, indicative of elevated trait heritabilities (*i.e.* typically *H^2^*>0.7). *H^2^* values were indeed significantly higher than in human populations, in particular for SBP, HR and ECG intervals (see for instance [36], [37] and [38]), where *H^2^* estimates typically vary around 0.6 in twin studies and around 0.25 in nuclear families. This further emphasises the potential of using our mouse model instead of traditional human cohorts for downstream genome-wide association scans. Third and regardless of the treatment conditions, strain means for all phenotypes had uni-modal distributions, consistent with the idea that they are complex traits under the control of multiple genetic loci.

We found that cardiovascular responses to drug exposure were trait-, drug-, and dose-specific. In order to obtain a comprehensive and unbiased overview of the structure of the phenotypic data with respect to the relationships across strains, treatments, and phenotypes, we performed a comprehensive comparative investigation. Our unsupervised bi-clustering analyses facilitated processing the large phenotypic data in order to extract key information regarding strain proximity and drug-dependent phenotypic perturbations. For instance our clustered matrices of significance score made immediately apparent that phenotypic changes were usually more significant under *β*-stimulation than *β*-blockade (consistent with *iso* and *ate* being administered to healthy animals housed in a reduced stress environment). When considering all experimental conditions, HR was the most, and SBP and QTc the least affected phenotypes in response to both pharmacological compounds. Strain responses of phenotypes related to body and heart weight (*i*.*e*. HW, AW, AWI, VW, VWI, BWE, BWG) were well correlated with drug-induced changes of heart rate and ECG intervals (in particular PR, Pdur, QT and ST), but not with the responses of systolic blood pressure and ECG wave amplitudes or areas. Trait-specific patterns of sensitivity to *iso* ranged from maximal response under treatment with *iso1* for HR to negligible effects under either concentrations of *iso* for SBP. We detected compartmental and strain-specific cardiac sensitivity to *iso*, with atria responding at lower concentrations than ventricles in the majority of the strains. At this stage, the biological mechanisms underlying differential sensitivities to chronic *β*-stimulation are not known but might reflect distinct and strain-specific distributions of atrial and ventricular *β*-adrenergic receptors and/or differential downstream signalling pathways. Altogether, these data suggest that responses to *β*-adrenergic drugs by themselves are determined by complex genetic architectures. Beyond the specific context of the present study, it is worth mentioning that the unsupervised bi-clustering analyses we employed in this study could also be useful for dissecting other large datasets of complex phenotypic traits, which are likely to become more and more frequent.

Due to their broad use to generate transgenic or knock-out models, the lines C57BL/6J and 129S1/SvImJ are of particular interest. Our comprehensive analyses as well as other reports [16], [34], [35] have clearly highlighted their differential behaviours for baseline phenotypes such as SBP, atrial and ventricular size, heart rate, heart rate variability and cardiac metabolism. Recently, Barrick *et al*. further emphasised on the specificity of 129S1/SvImJ and C57BL/6J responses to prolonged trans-aortic constriction (TAC), another model of left ventricular hypertrophy associated with increased hemodynamic load and sustained *β*-adrenergic stimulation [39]. Upon aortic banding, C57BL/6J mice had an earlier onset and more pronounced impairment in contractile function, with corresponding left and right ventricular dilatation, fibrosis, change in expression of hypertrophy markers, and increased liver weights at five weeks post-constriction. In contrast, 129S1/SvImJ mice had delayed transition to decompensated heart failure, with relatively mild alterations in histology and markers of hypertrophy at five weeks post-TAC and preserved systolic function until eight weeks post-TAC, suggesting that 129S1/SvImJ genetic modifiers might protect against the earlier and more severe pathological changes seen in C57BL/6J mice [39]. In contrast, in our study, *iso10*-mediated increase of VWI was marked in strain 129S1/SvImJ while C57BL/6J mice were significantly more resistant. This indicates that different signalling pathways may lead to cardiac hypertrophy, depending on the type of upstream trigger (*i.e.* pressure overload *vs* sustained *β-*adrenergic activation). These observations put an additional emphasis on the need for detailed characterising of these and other strains, both in terms of baseline cardiovascular phenotypes and in response to pressure overload or *β*-adrenergic challenge.

Future work will aim at associating the rich phenotypic data of this study with genetic markers. Indeed the vast majority of the strains of our CV-PGX panel has been extensively genotyped [24]. Early studies already suggested that the genome of laboratory mice is a mosaic of regions of distinct but limited sub-specific origins [40]. More recent resequencing efforts have refined this picture by cataloguing over 8 million SNP alleles across 16 inbred strains [41] and genetic maps of similar densities were further imputed in 49 strains [42]. These invaluable resources allow for inferring the ancestry of most of the genome with good confidence and provide mapping resolution usually higher than in other model organisms. Thus, it is now becoming feasible to map trait variation in inbred mouse strains by testing association to loci of inferred ancestry [24], [43], [44]. Here we already used genomic SNP profiles to show that overall genetic similarity of the strains exhibits little concordance with their phenotypic relatedness in terms of the collection of cardio-vascular traits we measured. This indicates that cardiovascular phenotypes are unlikely to segregate according to global phylogeny, but rather be governed by smaller, local differences in the genomes of the various strains. Thus, given the significant heritability of many of these traits, we are confident that association studies using our phenotypic resource have good chances to reveal new candidate loci related to differential cardiovascular responses under treatments.

## Materials and Methods

### Ethics Statement

All animal procedures have been approved by the “Service vétérinaire cantonal vaudois” (authorisation n° 1649) and were performed in accordance with the National Institutes of Health (NIH) guidelines for the care and use of laboratory animals.

### Mice

Inbred mouse strains were selected from the priority strains list of the Mouse Phenome Database (MPD: http://www.jax.org/phenome [15]), based on their genealogy and the density of SNPs characterised in each strain. Mice were purchased in two to six lots per strain, either from Charles River, France (strains Balb/cByJ, C3H/HeOuJ, CBA/J, and DBA/2J) or from the Jackson Laboratory at Bar Harbor, Maine, USA (strains 129S1/SvImJ, A/J, AKR/J, BALB/cJ, BTBRT^+^ *tf*/J, C3H/HeJ, C57BL6/J, C57BLKS/J, C58/J, FVB/NJ, I/LnJ, KK/HlJ, LP/J, NOD/ShiLtJ, NZB/BlNJ, PL/J, SJL/J, SM/J, and SWR/J). Only males, aged ten to twelve weeks on average, were included in the experimental protocol. Mice were housed at our local animal research facility under conditions of 14 hours light, 10 hours darkness, ambient temperature of 25°C, and relative humidity of 30–60%.

### Pharmacological agents and treatments

*Ate* and *iso* (Sigma-Aldrich) were administered to mice chronically for two weeks (**Figure 1**). Osmotic mini-pumps (Alzet, model 2002, Charles River Laboratories) were implanted sub-cutaneously under anaesthesia and set to deliver *ate* at 10 mg/kg per day and *iso* at 1 (*iso1*) and 10 (*iso10*) mg/kg per day. Based on the results of a pilot comparison of four C57BL/6J mice implanted with minipumps loaded with 0.9% NaCl *vs* six non-implanted isogenic animals, *ctr* mice were not implanted with minipumps.

### Phenotypic characterisation

A general outline of the phenotyping process is presented in **Figure 1**. In brief, heart rate (HR) and systolic blood pressure (SBP) were monitored in conscious animals by the tail-cuff (TC) method, using a Visitech BP-2000 blood pressure analysis system. All animals were trained for one week prior to recording effective values on days 10, 11, and 12 (**Figure 1**) and all measurements were taken at a similar time of the day (2–5 pm). Electrocardiograms (ECG) were recorded in halothane-anaesthetised mice using the IOX 1.7.0 and ECG-Auto 1.5.7 softwares (EMKA Technologies). On day 16 or 17, mice were weighted and sacrificed by decapitation. Hearts were rapidly excised from the animals, rinsed in ice-cold phosphate buffered saline (PBS) solution, and blotted dry. Cardiac atria and ventricles were dissected and weighted separately. Cardiac tissues were frozen in liquid nitrogen and stored at −70°C. Phenotypes recorded in the present project are listed in **Table 1**. Individual phenotypes were assessed in multiple series of independent experiments. In each strain, an average of ten individuals per experimental condition (*ctr*, *ate*, *iso1* and *iso10*) were monitored across all phenotypes.

### Data management and statistics

#### Heritability

The heritability *H^2^* of each phenotype was calculated separately for each treatment over all strains. It is defined as:
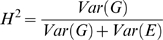
where *Var(G)* is the genetic (*i*.*e*. the inter-strain) variance, and *Var(E)* is the environmental (*i*.*e*. the intra-strain) variance of the considered phenotype.

#### Strain and phenotype clustering

Bi-Clustering was performed using the Matlab® standard clustering routine. Similarity between phenotype *p* and *p'* was computed as 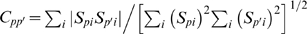. Similarity between strains *s* and *s*' was computed as 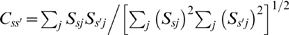. See main text for the definitions of 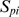 and 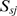. Note that both expressions are invariant under an inverse transformation of any phenotype.

#### Phenotypic versus genotypic relatedness

We use Pearson correlations 
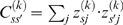
 both for phenotypic (*k* = *ctr*, *ate*, *iso1*, *iso10*) and genotypic (*k = G*) similarity. For the latter we first normalised the genotype of each SNP by taking the *z-*score across all strains. Here 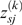 refers to the *z*-scores (the deviation from the mean value in units of standard deviation) for phenotypic (*j* runs across all phenotypes) or genotypic (*j* runs across all SNPs) profiles, respectively.

## Supporting Information

Supplement S1 Supplementary Results.
(1.57 MB PDF)
