# Supplementary material for: Cardiovascular Response to Beta-Adrenergic Blockade or Activation in 23 Inbred Mouse Strains
Source: PLoS One. 2009 Aug 12;4(8):e6610. doi: 10.1371/journal.pone.0006610 (PMC2722085; doi:10.1371/journal.pone.0006610)

## Supplementary Results

### Phenotypes measured in baseline condition and conservation across independent studies

Some of the traits measured in the present study, in particular SBP and HR, are notoriously sensitive to slight perturbations of environmental or experimental conditions. Therefore we assessed the consistency of our data with information available from independent experiments or laboratories. To do so, we calculated Pearson's and Spearman's correlations between our mean measurements in *ctr* condition and values from multistrain records of the MPD [1] across the common strains. These comparisons were restricted to MPD projects using inbred strains and experimental procedures similar with those of the present study. In the following we summarise our results:

*Body weight*

Average body weights of *ctr* animals ranged from 17±0.2 g in strain SM/J to 30±0.3 g in strain BTBRT$^{+}$*tf*/J at the start of the study (BWS), and from 18±0.5 g in strain SM/J to 30±0.8 g in strain KK/HlJ at the end of the procedure (BWE; **Figure 2**). The number of inbred lines available for comparisons with MPD datasets varied from 7 to 21, depending on the projects (**Suppl. Figure 3**). Pearson's $r^2$ values ranged from 0.52 to 0.93 ($p$<0.01) in 7 of the 9 (CV-PGX:MPD) pairs and from 0.39 to 0.95 ($p$<0.01) in 16 of the 36 (MPD:MPD) pairs examined (**Figure 3** and **Suppl. Figure 3**). Accordingly, Spearman's correlations were significant for most of the comparisons, indicating that both strain means and rankings are well conserved across independent projects (data not shown). Altogether, these results show that body weight is a very robust phenotype and that CV-PGX values are well consistent with other data.

*Heart weight and cardiac weight indices*

Heart weight (HW) of *ctr* mice ranged from 77±2.8 mg in strain A/J to 129±3.5 mg in strain NZB/BlNJ. Mean HW as well as atrial weight (AW), atrial weight index (AWI), ventricular weight (VW) and ventricular weight index (VWI) had uni-modal distributions across strains, without distinct sub-groups. Two projects of the MPD (Deschepper1 [2] and Jaxpheno2) contain multistrain datasets for HW and related cardiac weight indices (see correlation matrices for VW, VWI, AW, AWI, and HW in **Suppl. Figure 3**, and VWI and AWI panels in **Figure 3**). Close proximity of our measurements with the data of Deschepper was essentially reflected by strong correlations of VWI means and rankings (Pearson $r^2$=0.94, Spearman $\rho^2$=0.93, $p$<$10^{-20}$; **Figure 3**). In contrast, measurements of HW and BW in the

Jaxpheno2 study correlates poorly with CV-PGX data. Yet, since this study was also discordant with other MPD records, including those of Deschepper, we believe that HW and BW, and in particular its ratio (i.e. VWI) are nevertheless likely to be well reproducible phenotypes.

Results were more divergent for cardiac atria, as average AWI of most strains was lower in the CV-PGX study than in Deschepper's (Pearson $r^2$=0.21, Spearman $\rho^2$=0.36, $p$=0.07; **Figure 3**). This is most likely due to the small size of the atria and may be a result of subtle differences in the dissection process rather than intrinsic strain variability.

*Systolic blood pressure and heart rate*

In conscious *ctr* mice, average SBP varied from 86±8 mmHg in strain BTBRT$^+$*tf*/J to 119±6 mmHg in strain SWR/J, whereas average HR extended from 442±35 beats/min in strain Balb/cByJ to 713±44 beats/min in strain SJL/J (**Figure 2**). Both variables were approximately uniformly distributed across strains but the patterns of distribution were not correlated (*i.e*. correlation $r$=0.25, $p$=0.26; **Suppl. Figure 6A**). HR means of closely related strains (*i.e*. strains C3H/HeJ *vs* C3H/HeOuJ and Balb/cJ *vs* Balb/cByJ) were very similar, while SBP values were slightly more divergent. Uni-modal distribution of baseline HR suggests that strains with high pulse have higher baseline sympathetic and lower parasympathetic activity than strains with low pulse. This is consistent with the idea that HR is an indicator of the strain-specific homeostatic state of autonomous nervous system activity [3].

The MPD contains four, respectively five multistrain datasets of mean SBP and HR measured by tail-cuff, and two sets of mean HR determined by ECG monitoring of conscious animals. The sensitivity of both traits to non-genetic perturbations is reflected by the relatively low correlations of these records, even though the number of strains included in the comparisons is relatively small (**Suppl. Figure 3**). Thus, only one of the nine possible pair-wise comparisons between distinct SBP datasets (*i.e*. Deschepper1 *vs* Svenson1, 7 strains in common, Pearson's $r^2$=0.76, $p$<0.05; **Suppl. Figure 3**) and two of the thirteen possible combinations between HR records are significantly correlated (*i.e*. Jaxwest1 pulse and Hampton1, 7 strains in common, Pearson's $r^2$=0.81, $p$<0.01; Sugiyama1 and Hampton1, 13 strains in common, $r^2$=0.37, $p$<0.05). CV-PGX pressure and pulse values were significantly correlated with the data of Sugiyama1 for SBP (14 strains in common with the CV-PGX dataset, $r^2$=0.63, $p$<0.01 [4]) and with those of Jaxwest1 and Hampton1 projects for HR (Jaxwest1: 7 strains in common with the CV-PGX dataset, $r^2$=0.74, $p$<0.05; Hampton1: 16 strains in common with the CV-PGX dataset, $r^2$=0.40, $p$<0.01 [5]), while the other (CV-PGX:MPD) pairs were more discordant

(**Suppl. Figure 3**). The numbers of positively correlated (CV-PGX:MPD) datasets corroborate those of the (MPD:MPD) comparisons. Altogether, they are higher than expected by chance (*i.e.* 2/14 have a *p*-value<0.05 for SBP, and 4/19 have *p*<0.05 for HR), suggesting that despite marked susceptibility to the environment, both blood pressure and heart rate are also controlled in part by genetic determinants.

**Details on phenotypes measured under atenolol or isoproterenol treatment**

*Heart rate under anaesthesia*

The opposite action of the drugs on pulse rate was clearly detected under anaesthesia (see HR (ECG) panel in **Figures 2** and **4**), but means, amplitudes and significance of responses were generally lower than in conscious mice. Thus, maximal and minimal values were attained at 618±25 beats/min in strain C57BL/6J treated with *iso10* and at 297±27 beats/min in strain C57BLKS/J infused with *ate*. The chronotropic effect of *iso10* reached significance (*p*<0.05) in nine strains only, the positive dose-effect of *iso* was observed in a single strain (SM/J), and the blocking effect of *ate* was close to negligible in sixteen strains (**Figure 4**). In agreement with results obtained in baseline condition, correlations between pulse measured in conscious animals or in anaesthetised mice were weak, irrespective of the drug treatment (**Suppl. Figures 3** and **6**). This latter information further emphasises the strain-specific confounding action of anaesthesia on pacing responses.

*ECG intervals under anaesthesia*

The overall modifications induced by both drugs on PR, QRS, and QT intervals of ECG waves followed expected trends in the majority of the strains, even though these measurements suffered from the confounding effects of anaesthesia. Treatment with *iso10* induced a significant decrease of PR in ten strains, of QRS in four strains, and of QT in four strains (**Figures 2** and **4**). The same trends applied to the majority of the other strains without reaching nominal significance (*p*<0.05), whereas the opposite effect of *ate* was milder. Interestingly, the chronotropic action of *iso* was often not distributed equivalently across all ECG intervals. In strain KK/HlJ for instance, QRS interval was dose-dependently reduced under *β*-stimulation. This decrease was matched by a slight increase of ST (data not shown), whereas HR, PR, and QT were barely affected. In contrast, QRS shortening was paralleled by increased HR and decreased PR and/or QT intervals in strains Balb/cByJ, C3H/HeJ and to a lesser extent FVB/NJ. Similarly, the reduction of QT intervals under *β*-stimulation was matched by

a higher pulse rate in seven strains, whereas HR was hardly affected in SJL/J mice. Also, the increase of HR under *iso* was not systematically paralleled by significantly shorter QT. In A/J mice, QT was even slightly increased, whereas the most stringent positive chronotropy affected PR intervals. Altogether, these interval-specific patterns argue in favour of differential and strain-specific genetic modulators of cardiac electrical activity.

*Body weight*

As mentioned in the main text body, stimulation with *iso10* was paralleled by an elevated gain of body mass in strain C58/J when compared to untreated individuals. Thus, *ctr* mice gained 0.5±0.27 g over the two weeks of the experiment (*i.e*. a relative increase of 2.2% compared to BWS), whereas animals exposed to *iso10* increased their weight by 2.9±0.42 g (+13.6%). The same trend was significant in nine additional strains placed under *iso10* stimulation ($p$<0.05), whereas little change was seen in the others (**Figures 2** and **4**). The maximum relative difference of BWG between *ctr* and *iso10*-treated individuals reached 14.5% of BWS in strain 129S1/SvImJ (*i.e*. *ctr*: -2.4%; *iso10*: +12.1%). These data are consistent with observations made previously in CD-1 mice, in which chronic administration of 30 mg/kg *iso* per day for 13 days not only led to increased HW, but also to higher BWG (*i.e*. relative increases of 22% in *iso*-treated *vs* 8% in *ctr* mice, when compared to BWS [6]. Further experimental evidence relating elevated BWG with augmented food consumption, muscle mass, and brown fat, as observed in CD-1 mice, is currently lacking. The range of extra body weight gained under *iso10* was strain-specific (**Figures 2** and **4**). Concomitantly, we observed slightly weaker correlations of (CV-$PGX_{iso10}$:MPD) than (CV-$PGX_{ctr}$:MPD) pair-wise comparisons (**Suppl. Figure 3**).

The impact of *β*-blockade on body mass was negligible in most strains. Strain A/J however behaved in a peculiar manner as these mice tended to loose weight under all treatments, while BW of *ctr* animals remained steady. BW loss was significant under *ate* and *iso10*, reaching an average of 3.4±0.6 g under *ate*, (*i.e*. -15.9% of BWS), 1.2±0.4 g under *iso1* (*i.e*. -5.8% of BWS) and 1.4±0.4 g under *iso10* (*i.e*. 6.9% of BWS). It is unlikely that these losses were caused by an undetected diseased-state as both treated and untreated animals were housed together. Moreover a similar trend, although statistically not significant, was recently reported in 12-15 wk-old A/J males challenged with five consecutive daily doses of 100 mg/kg *iso* [7,8].

*Correcting for multiple testing*

The *p*-values reported in **Figure 4** and **Suppl. Figure 4** are obtained from the Wilcoxon ranksum test. They reflect the probability of the observed data for a particular phenotype *p* and strain *s*, given that the distributions for two different treatments (*ctr vs ate*, *iso1*, *iso10*, or *iso1 vs iso10*) have the same median value (the null hypothesis). Small *p*-values indicate strong evidence for rejecting the null hypothesis, *i.e*. suggesting that those phenotypes have different medians. We reshuffled 10'000 times the data amongst each two treatment groups and computed *p*-values from these permutations (corresponding to the null hypothesis) as controls. The fraction of controls with a ranksum statistic larger than the one observed for non-permuted data gives an estimate of the *p*-value that is independent of the phenotypic distribution. We find that the ranksum *p*-values (as reported in **Figure 4**) are well-calibrated with respect to the permutation *p*-values (in contrast to those from *t*-tests, see **Suppl. Figure 11**).

When judging the significance of a statistical test within the context of many tests, the significance threshold has to be adjusted. In the well known Bonferroni correction the nominal significance threshold $\alpha$=0.05 is lowered to $\alpha'=\alpha/N$ (*N* being the number of tests). For example, focusing on one phenotype but looking simultaneously at *N*=23 strains gives $\alpha'\approx0.002$. Similarly, focusing on one strain but considering all *N*=27 phenotypic response variables also gives $\alpha'\approx0.002$.

The Bonferroni correction procedure is very conservative because it controls the family-wise error rate, keeping low the number of false positives (FP), but also true positives (TP). A more appropriate alternative is to control the false discovery rate (FDR), i.e. FP/(FP+TP). We used the Benjamini-Hochberg (BH) step-up procedure to control the FDR [9], but - to account for the dependencies of the tests - obtained the null distribution of *p*-values from 10'000 random permutations of the phenotypes as was done in [10]. BH-corrected *p*-values for each phenotype (considered over all strains) are shown in **Suppl. Figure 12**. Conversely, BH-corrected *p*-values for each strain (considered over all phenotypes) are shown in **Suppl. Figure 13**. Moreover, in **Suppl. Figure 14** we indicate globally adjusted significance values, correcting for both multiple strains and phenotypes. In **Suppl. Figure 15** we plotted BH-corrected *p*-values against the uncorrected *p*-values. This figure highlights the increase in power if one is willing to control the FDR, rather than the family-wise error rate. Indeed most BH-corrections are significantly milder than those obtained by the Bonferroni correction (which would result in all points being on the black lines). This is also evidenced in **Suppl. Figure 16** and **Suppl.**

**Figure 17** which compare the number of significant observations with BH- and Bonferroni-corrections, respectively, against those without any corrections. Indeed, while more than half of the nominally significant observations (without any corrections) involving an *iso1* and/or *iso10*-treated group of animals remain significant with BH-correction, less than 5% would survive the Bonferroni-correction. For *ate vs ctr* (which gave only 107 nominally significant observations to start with), a single observation (HR of C57BLKS) passes the Bonferroni-correction, while almost a third remains significant after BH-correction.

## Supplementary Materials and Methods

### Data management and statistics

*z-scores and correlations within drug conditions*

For each phenotype *p*, strain *s* and treatment, a *z*-score was calculated as:

$$z_{ps} = \frac{x_{ps} - \mu_p}{\sigma_p}$$

where $x_{ps}$ is the mean of phenotype *p* under a given treatment (*t=ctr, ate, iso1, iso10*) in strain *s*, and $\mu_p$ and $\sigma_p$ are the mean and the standard deviation, respectively, of the average measurement for phenotype *p* across all strains. To address the structure of the *z*-scores, we built four matrices (one per drug condition), each containing one row for each strain *s* and one column for each phenotype *p*. Hierarchical bi-clustering was applied to determine the patterns of strain and phenotype similarities in the matrices. Phenotypes were clustered according to absolute similarity while strains were clustered according to signed similarity. The branches of the dendrograms illustrating the clusters were plotted with the same colour as long as the average linkage distance was less than 20% of the maximal distance.

**Supplementary Figure legends**

**Supplementary Figure 1**

**Phenotypic strain means.** Bar graphs representing the full dataset of phenotypic strain means and standard deviations across the four drug conditions. White bars: *ctr*; blue bars: *ate*; orange bars: *iso1*; red bars: *iso10*. Strains are ranked by increasing HR (TC) means of *ctr* mice. See **Table 1** for abbreviations. HR std: standard deviation of HR (TC) strain means; SBP std: standard deviation of SBP strain means.

**Supplementary Figure 2**

**Heritabilities.** Heritabilities ($H^2$) were calculated separately for each phenotype and each treatment over all strains and are presented in a colour-code using the colour scale shown on the right. Every column represents a separate treatment, while every row denotes a phenotype. Phenotypes are highly heritable within the set of CV-PGX mouse strains, with $H^2$ ranging from 0.53 to 0.98.

**Supplementary Figure 3**

**Comparison of CV-PGX and MPD strain means across fourteen phenotypes.** Each matrix summarises the data of all pair-wise Pearson's comparisons between and across CV-PGX and MPD projects for a single phenotype. Pearson $r^2$ values are indicated in the top triangle of the matrix, the significance of the correlations is shown by a colour scale, and the number of strains considered for each pair-wise comparison is indicated in the bottom triangle of the matrix. Underlined $r^2$ values denote negative correlations. Abbreviations are given in **Table 1** for phenotypes and in the boxed legend for multistrain MPD datasets. Detailed experimental protocols and links to original publications of the MPD projects are available at http://www.jax.org/phenome [1]. Of note, Hampton1 and Jaxwest1 HR means were recorded by ECG in conscious mice, whereas all other HR means, including Jaxwest1 pulse, were obtained by a tail-cuff system. SBP was measured by tail-cuff in all projects. In complement to **Figure 3**, this figure also shows the comparisons of CV-PGX means recorded in *ate*- and *iso*-treated mice with CV-PGX and MPD data measured in untreated animals.

**Supplementary Figure 4**

**Intra-strain significance of drug treatments.** Data are presented for the phenotypes shown in **Suppl. Figure 1** as in **Figure 4** (main text). Intra-strain *p*-values of phenotypic data recorded in treated

*vs* untreated groups (Wilcoxon ranksum statistics) are presented as bar graphs on a $-\log_{10}$ scale. The threshold of significance is indicated by red lines ($p$=0.05) and -log $p$-values are signed according to the directionality of the effect induced by the drugs. When significant, $p$-values for testing responses under *iso10 vs iso1* are indicated by coloured stars (*i.e.* *: $p<0.05$; **: $p<0.01$; ***: $p<0.001$; red star: phenotypic mean under *iso10* > phenotypic mean under *iso1*; blue star: phenotypic mean under *iso10* < phenotypic mean under *iso1*). Blue bars: *ate vs ctr*; orange bars: *iso1 vs ctr*; red bars: *iso10 vs ctr*. *P*-values smaller than 0.001 (bars extending beyond the green lines or ***) hold up against Bonferroni correction for multiple testing of either all strains for a given phenotype or all phenotypes for a given strain. Strains are ranked as in **Figure 2** and **Suppl. Figure 1**. See **Table 1** for abbreviations.

**Supplementary Figure 5**

***z*-scores across strains and phenotypes in each of the four treatment conditions.** *z*-scores, calculated as indicated in **Suppl. Materials and Methods**, are presented in a colour scale. **A**: *ctr*. **B**: *ate*. **C**: *iso1*. **D**: *iso10*. Rows and columns are clustered according to pattern similarity. The branches of the dendrograms illustrating the clusters are shown in the same colour as long as the average linkage distance is less than 20% of the maximal distance.

**Supplementary Figure 6**

**Symmetric pair-wise correlations of the strain patterns of *z*-scores across all phenotypes in each of the four treatment conditions.** **A**: *ctr*. **B**: *ate*. **C**: *iso1*. **D**: *iso10*. Signed *z*-scores are presented in **Suppl. Figure 5**. Rows and columns are clustered according to pattern similarity. The branches of the dendrograms illustrating the clusters are shown in the same colour as long as the average linkage distance is less than 20% of the maximal distance.

**Supplementary Figure 7**

**Symmetric pair-wise correlations of the trait patterns of *z*-scores across all strains in each of the four treatment conditions.** **A**: *ctr*. **B**: *ate*. **C**: *iso1*. **D**: *iso10*. Signed *z*-scores are presented in **Suppl. Figure 5**. Strains are clustered according to pattern similarity. The branches of the dendrograms illustrating the clusters are shown in the same colour as long as the average linkage distance is less than 20% of the maximal distance.

**Supplementary Figure 8**

**A.** Symmetric pair-wise correlations of the trait patterns of signed *p*-values across all treated-strains. Signed *p*-values are presented in **Figure 5**. Traits are clustered according to pattern similarity. **B.** Symmetric pair-wise correlations of the treated-strain patterns of signed *p*-values across all phenotypes. Signed *p*-values are presented in **Figure 5**. Treated-strains are clustered according to pattern similarity. The branches of the dendrograms illustrating the clusters are shown in the same colour as long as the average linkage distance is less than 20% of the maximal distance.

**Supplementary Figure 9**

**A.** Symmetric pair-wise correlations of the strain patterns of signed *p*-values across all treated-phenotypes. Signed *p*-values are presented in **Figure 6**. Strains are clustered according to pattern similarity. **B.** Symmetric pair-wise correlations of the treated-phenotype patterns of signed *p*-values across all strains. Signed *p*-values are presented in **Figure 6**. Treated phenotypes are clustered according to pattern similarity. The branches of the dendrograms illustrating the clusters are shown in the same colour as long as the average linkage distance is less than 50% of the maximal distance.

**Supplementary Figure 10**

**Symmetric pair-wise correlations of the patterns of SNPs across 21 strains of the CV-PGX project.** SNP configurations were obtained from the Broad1 SNP dataset [11] available through the MPD [1]. Strains are clustered according to pattern similarity. The branches of the dendrograms illustrating the clusters are shown in the same colour as long as the average linkage distance is less than 20% of the maximal distance.

**Supplementary Figure 11**

**Comparison of *p*-values from ranksum test to those obtained from 10’000 controls.** For the controls, the data were reshuffled between the two treatment groups and *p*-values were estimated by the fraction of controls with a ranksum statistic larger than the one observed for non-permuted data. This gives an estimate of the *p*-value that is independent of the phenotypic distribution. We find that the ranksum *p*-values (left) correspond well to the permutation *p*-values, except the smallest ones which cannot be estimated accurately with 10’000 permutations. In contrast, the *p*-values from *t*-tests (right) appear to be inflated when compared to those obtained from permutations.

**Supplementary Figure 12**

**Intra-strain significance of drug treatments corrected for testing multiple strains by the Benjamini-Hochberg procedure.** These *p*-values are adjusted such that FDR<0.05 when judging the significance of each given phenotype for all strains. See **Figure 4** in the main text and **Suppl. Results** section for details.

**Supplementary Figure 13**

**Intra-strain significance of drug treatments corrected for testing multiple phenotypes by the Benjamini-Hochberg procedure.** These *p*-values are adjusted such that FDR<0.05 when judging the significance of each given strain for all phenotypes. See **Figure 4** in the main text and **Suppl. Results** section for details.

**Supplementary Figure 14**

**Intra-strain significance of drug treatments corrected for testing multiple strains and phenotypes by the Benjamini-Hochberg procedure.** These *p*-values are adjusted such that FDR<0.05 when judging the significance of a given strain and phenotype in the context of all strains and phenotypes. See **Figure 4** in the main text and **Suppl. Results** section for details.

**Supplementary Figure 15**

**Scatter-plots of Benjamini-Hochberg-corrected *p*-values against the uncorrected *p*-values.** For each treatment comparison, *p*-values corrected by Benjamini-Hochberg step-up procedure (*y*-axis) are plotted against the uncorrected values (*x*-axis). **A.** Global corrections (strains and phenotypes). **B.** Correcting for testing multiple strains. **C.** Correcting for testing multiple phenotypes. The colour shades in panels B and C show the different phenotypes and strains, respectively. The green line represents equality between BH-corrected and nominal *p*-values. The black line represents the theoretical Bonferroni correction.

**Supplementary Figure 16**

**Comparison of the number of significant observations with Benjamini-Hochberg-corrected *p*-values against those with uncorrected *p*-values. A.** Global corrections (strains and phenotypes). **B.** Correcting for testing multiple strains. **C.** Correcting for testing multiple phenotypes. The ratio between

the number of significant observations with Benjamini-Hochberg-corrected *p*-values over that with uncorrected *p*-values was used to set the background colour of each field (see colour bar).

**Supplementary Figure 17**

**Comparison of the number of significant observations with Bonferroni-corrected *p*-values against those with uncorrected *p*-values. A.** Global corrections (strains and phenotypes). **B.** Correcting for testing multiple strains. **C.** Correcting for testing multiple phenotypes. The ratio between the number of significant observations with Bonferroni-corrected *p*-values over those with uncorrected *p*-values was used to set the background colour of each field (see colour bar).

Reference List


1. Grubb SC, Maddatu TP, Bult CJ, Bogue MA (2009) Mouse phenome database. Nucleic Acids Res 37: D720-D730. 10.1093/nar/gkn778.

2. Deschepper CF, Olson JL, Otis M, Gallo-Payet N (2004) Characterization of blood pressure and morphological traits in cardiovascular-related organs in 13 different inbred mouse strains. J Appl Physiol 97: 369-376.

3. Shusterman V, Usiene I, Harrigal C, Lee JS, Kubota T et al. (2002) Strain-specific patterns of autonomic nervous system activity and heart failure susceptibility in mice. Am J Physiol Heart Circ Physiol 282: H2076-H2083.

4. Tsukahara C, Sugiyama F, Paigen B, Kunita S, Yagami K (2004) Blood pressure in 15 inbred mouse strains and its lack of relation with obesity and insulin resistance in the progeny of an NZO/HILtJ x C3H/HeJ intercross. Mamm Genome 15: 943-950.

5. Chu V, Otero JM, Lopez O, Morgan JP, Amende I et al. (2001) Method for non-invasively recording electrocardiograms in conscious mice. BMC Physiol 1: 6.

6. Kudej RK, Iwase M, Uechi M, Vatner DE, Oka N et al. (1997) Effects of chronic beta-adrenergic receptor stimulation in mice. J Mol Cell Cardiol 29: 2735-2746.

7. Faulx MD, Ernsberger P, Vatner D, Hoffman RD, Lewis W et al. (2005) Strain-dependent beta-adrenergic receptor function influences myocardial responses to isoproterenol stimulation in mice. Am J Physiol Heart Circ Physiol 289: H30-H36.

8. Faulx MD, Chandler MP, Zawaneh MS, Stanley WC, Hoit BD (2007) Mouse strain-specific differences in cardiac metabolic enzyme activities observed in a model of isoproterenol-induced cardiac hypertrophy. Clin Exp Pharmacol Physiol 34: 77-80. 10.1111/j.1440-1681.2007.04531.x.

9. Benjamini Y, Hochberg Y (1995) Controlling the false discovery rate - A practical and powerful approach to multiple testing. Journal of the Royal Statistical Society Series B (Methodological) 57: 289-300.

10. Yekutieli D, Benjamini Y (1999) Resampling-based false discovery rate controlling multiple test procedures for correlated test statistics. Journal of Statistical Planning and Inference 82: 171-196.

11. Wade CM, Daly MJ (2005) Genetic variation in laboratory mice. Nat Genet 37: 1175-1180.

Suppl. Figure 1 part 1

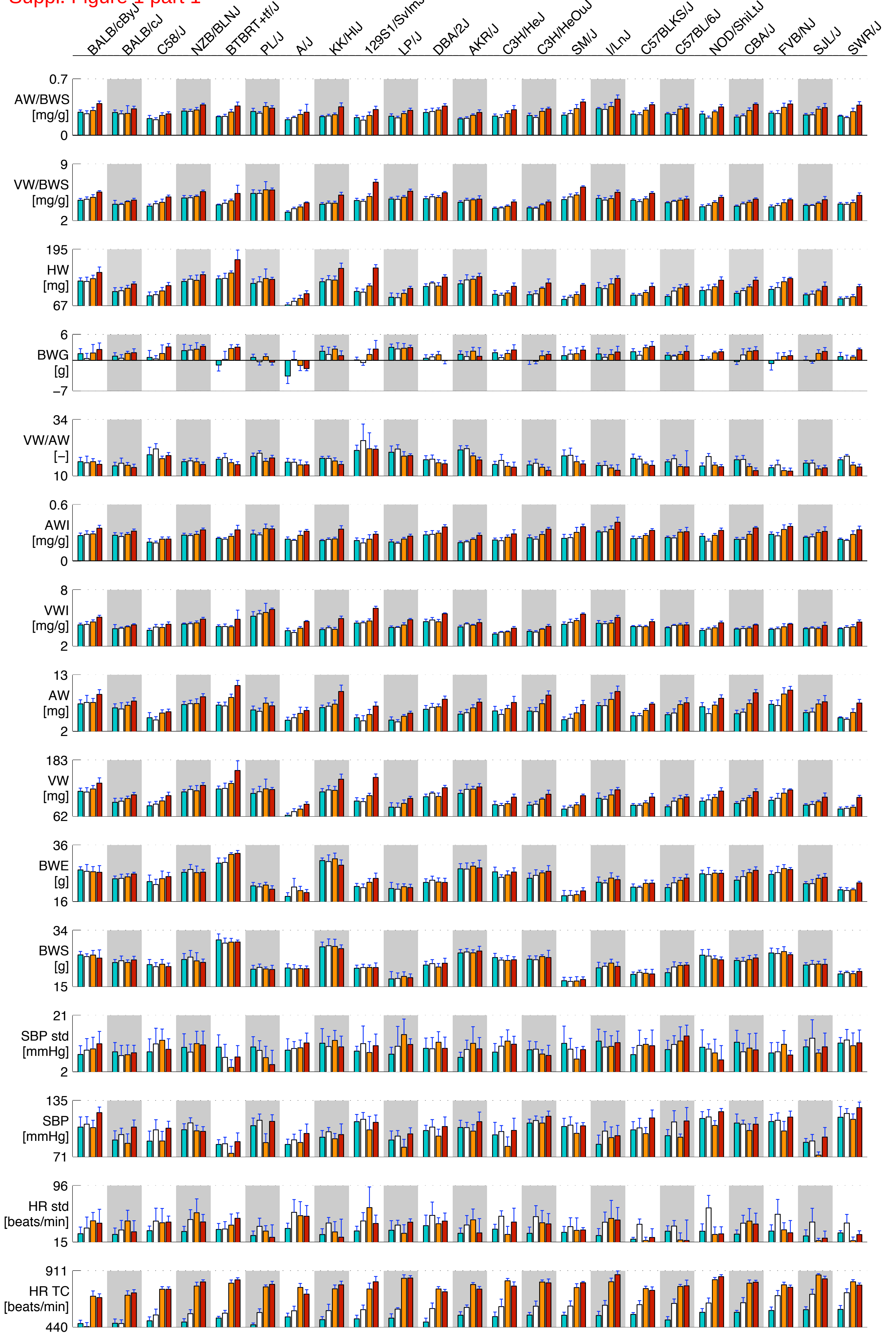

Suppl. Figure 1 part 2

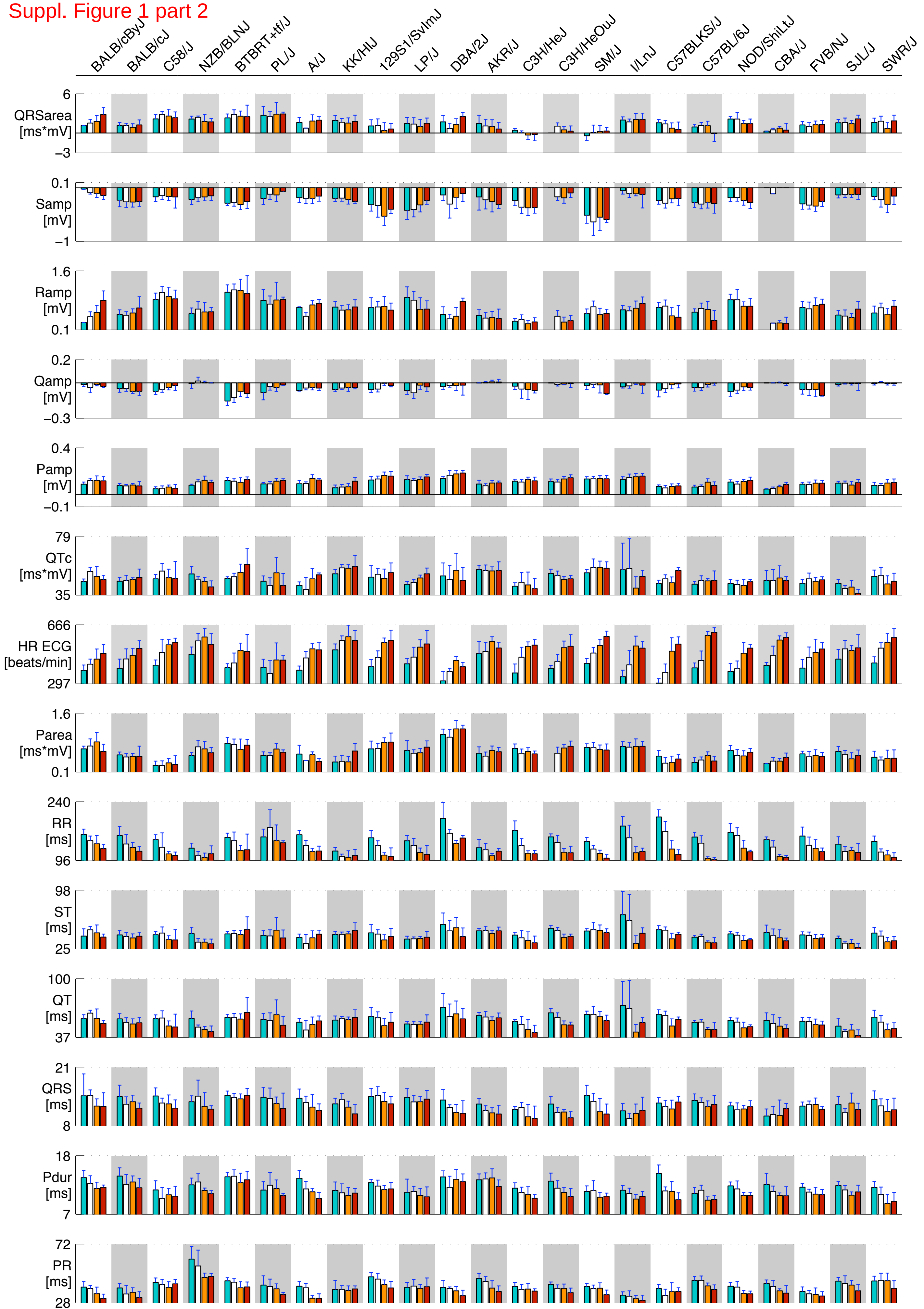

Suppl. Figure 2

Heritability

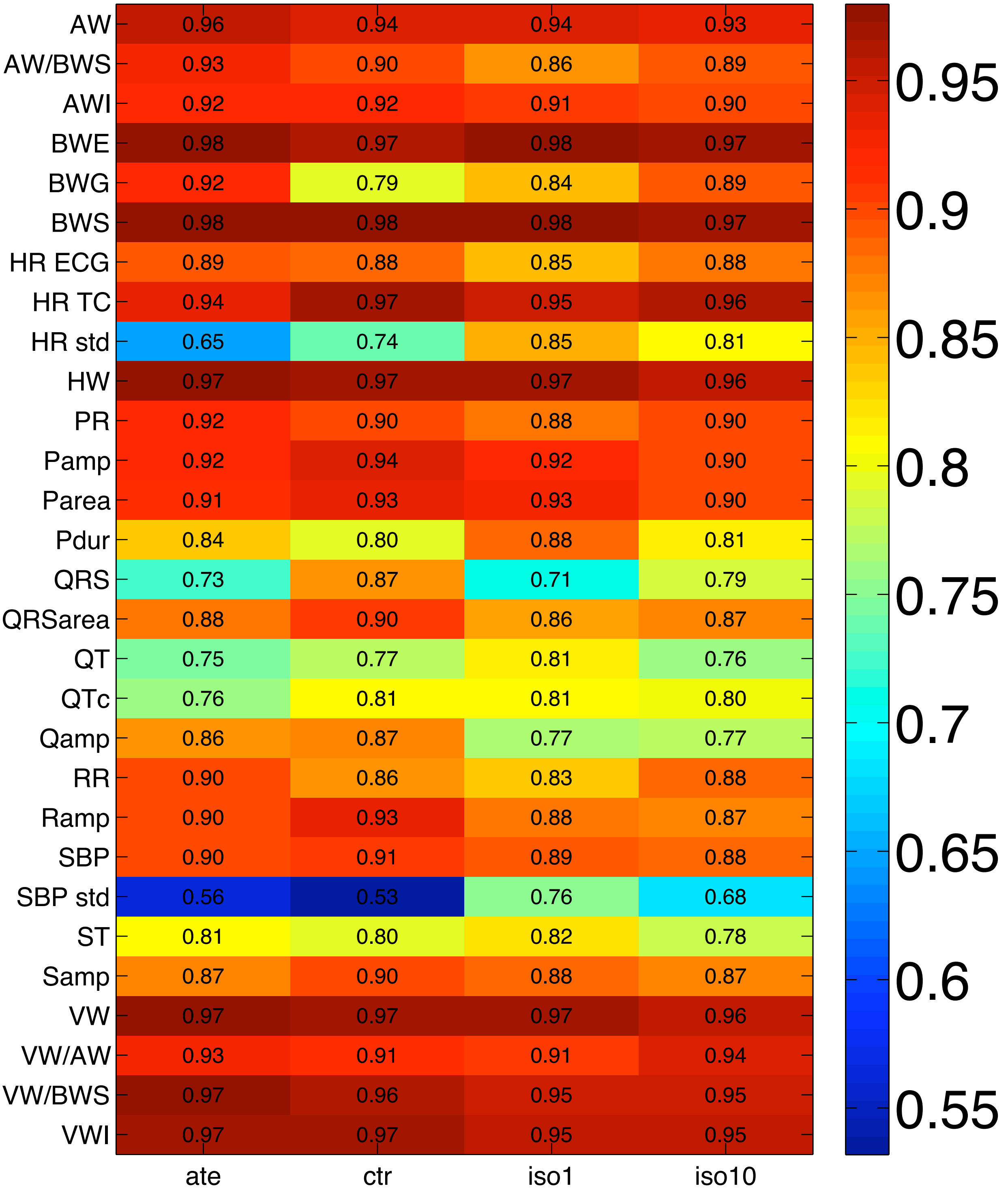

# Supplementary Figure 3

underlined : r is negative

Upper triangle: Pearson $r^2$; lower triangle: nb of strains in common

| Colour | Significance |
|---|---|
| red | p<0.001 |
| orange | p<0.01 |
| yellow | p<0.05 |
| white | ns |

**MULTISTRAIN DATASETS**

| | | | |
|---|---|---|---|
| control | present study | JaxW1 | Jaxwest 1 |
| atenolol | present study | Pai1 | Paigen 1 |
| iso 1 | present study | Seb1 | Seburn 1 |
| iso 10 | present study | Sug1 | Sugiyama 1 |
| Des1 | Deschepper 1 | Sve1 | Svenson 1 |
| Gav1 | Gavras 1 | Tor2 | Tordoff 2 |
| Ham1 | Hampton 1 | Tor3 | Tordoff 3 |
| JaxP_8 | Jaxpheno 2 / 8 wks | Wil1 | Willott 1 |
| JaxP_16 | Jaxpheno 2 / 16 wks | | |

**BWE**

| BWE | con | atenolol | iso 1 | iso 10 | BWS | Tor2 | Wil1 | Seb1 | Des1 | Pai1 | JaxP_8 | JaxP_16 | Tor3 | JaxW1 |
|---|---|---|---|---|---|---|---|---|---|---|---|---|---|---|
| con | | 0.905 | 0.916 | 0.796 | 0.92 | 0.842 | 0.521 | 0.774 | 0.641 | 0.701 | 0.067 | 0.023 | 0.86 | 0.929 |
| atenolol | 23 | | 0.9 | 0.81 | 0.846 | 0.71 | 0.453 | 0.677 | 0.378 | 0.65 | 0.103 | 0.324 | 0.756 | 0.575 |
| iso 1 | 23 | 23 | | 0.915 | 0.924 | 0.809 | 0.635 | 0.731 | 0.501 | 0.64 | 0.203 | 0.002 | 0.78 | 0.423 |
| iso 10 | 23 | 23 | 23 | | 0.839 | 0.681 | 0.63 | 0.655 | 0.37 | 0.561 | 0.31 | 0.003 | 0.652 | 0.237 |
| BWS | 23 | 23 | 23 | 23 | | 0.818 | 0.628 | 0.637 | 0.617 | 0.554 | 0.107 | 0.051 | 0.747 | 0.666 |
| Tor2 | 17 | 17 | 17 | 17 | 17 | | 0.686 | 0.889 | 0.846 | 0.763 | 0.085 | 0.113 | 0.844 | 0.646 |
| Wil1 | 21 | 21 | 21 | 21 | 21 | 23 | | 0.774 | 0.869 | 0.39 | 0.466 | 0.077 | 0.608 | 0.119 |
| Seb1 | 15 | 15 | 15 | 15 | 15 | 13 | 16 | | 0.772 | 0.837 | 0.282 | 0.239 | 0.889 | 0.729 |
| Des1 | 10 | 10 | 10 | 10 | 10 | 11 | 12 | 9 | | 0.872 | 0.227 | 0.082 | 0.946 | 0.12 |
| Pai1 | 21 | 21 | 21 | 21 | 21 | 24 | 30 | 16 | 11 | | 0.254 | 0.038 | 0.848 | 0.527 |
| JaxP_8 | 8 | 8 | 8 | 8 | 8 | 7 | 7 | 6 | 6 | 7 | | 0.243 | 0.035 | 0.438 |
| JaxP_16 | 7 | 7 | 7 | 7 | 7 | 7 | 6 | 5 | 6 | 6 | 9 | | 0.09 | 0.255 |
| Tor3 | 21 | 21 | 21 | 21 | 21 | 24 | 39 | 15 | 13 | 29 | 7 | 7 | | 0.905 |
| JaxW1 | 7 | 7 | 7 | 7 | 7 | 5 | 7 | 6 | 5 | 7 | 4 | 3 | 6 | |

**VW**

| VW | con | atenolol | iso 1 | iso 10 | Des1 |
|---|---|---|---|---|---|
| con | | 0.941 | 0.903 | 0.649 | 0.873 |
| atenolol | 23 | | 0.913 | 0.727 | 0.76 |
| iso 1 | 23 | 23 | | 0.742 | 0.718 |
| iso 10 | 23 | 23 | 23 | | 0.552 |
| Des1 | 10 | 10 | 10 | 10 | |

**AW**

| AW | con | atenolol | iso 1 | iso 10 | Des1 |
|---|---|---|---|---|---|
| con | | 0.859 | 0.748 | 0.664 | 0.276 |
| atenolol | 23 | | 0.754 | 0.648 | 0.283 |
| iso 1 | 23 | 23 | | 0.743 | 0.379 |
| iso 10 | 23 | 23 | 23 | | 0.428 |
| Des1 | 10 | 10 | 10 | 10 | |

**VWI**

| VWI | con | atenolol | iso 1 | iso 10 | Des1 |
|---|---|---|---|---|---|
| con | | 0.916 | 0.877 | 0.648 | 0.944 |
| atenolol | 23 | | 0.905 | 0.736 | 0.841 |
| iso 1 | 23 | 23 | | 0.711 | 0.777 |
| iso 10 | 23 | 23 | 23 | | 0.534 |
| Des1 | 10 | 10 | 10 | 10 | |

**AWI**

| AWI | con | atenolol | iso 1 | iso 10 | Des1 |
|---|---|---|---|---|---|
| con | | 0.817 | 0.685 | 0.64 | 0.214 |
| atenolol | 23 | | 0.755 | 0.597 | 0.381 |
| iso 1 | 23 | 23 | | 0.58 | 0.373 |
| iso 10 | 23 | 23 | 23 | | 0.447 |
| Des1 | 10 | 10 | 10 | 10 | |

**HW**

| HW | con | atenolol | iso 1 | iso 10 | JaxP_8 | JaxP_16 | Des1 |
|---|---|---|---|---|---|---|---|
| con | | 0.941 | 0.898 | 0.651 | 0.301 | 0.057 | 0.859 |
| atenolol | 23 | | 0.905 | 0.727 | 0.049 | 0.024 | 0.731 |
| iso 1 | 23 | 23 | | 0.74 | 0.053 | 0.003 | 0.702 |
| iso 10 | 23 | 23 | 23 | | 0.091 | 0.011 | 0.551 |
| JaxP_8 | 8 | 8 | 8 | 8 | | 0.562 | 0.273 |
| JaxP_16 | 7 | 7 | 7 | 7 | 7 | | 0.058 |
| Des1 | 10 | 10 | 10 | 10 | 6 | 6 | |

**HR**

| HR | con | atenolol | iso 1 | iso 10 | ECG_HR | Gav1 | Ham1 | JaxW1 HR | JaxW1 pulse | Sve1 | Sug1 |
|---|---|---|---|---|---|---|---|---|---|---|---|
| con | | 0.741 | 0.535 | 0.217 | 0.02 | 0.078 | 0.4 | 0.399 | 0.743 | 0.211 | 0.121 |
| atenolol | 23 | | 0.462 | 0.155 | 5E-04 | 0.168 | 0.245 | 0.213 | 0.656 | 0.582 | 0.135 |
| iso 1 | 23 | 23 | | 0.572 | 0.075 | 0.055 | 0.386 | 0.309 | 0.794 | 0.296 | 0.004 |
| iso 10 | 23 | 23 | 23 | | 0.093 | 0.443 | 0.281 | 0.725 | 0.435 | 0.236 | 0.003 |
| ECG_HR | 23 | 23 | 23 | 23 | | 0.101 | 0.048 | 0.024 | 0.017 | 0.583 | 0.011 |
| Gav1 | 6 | 6 | 6 | 6 | 6 | | 0.004 | na | na | 0.526 | 0.065 |
| Ham1 | 16 | 16 | 16 | 16 | 16 | 6 | | 0.459 | 0.806 | 0.001 | 0.369 |
| JaxW1 HR | 7 | 7 | 7 | 7 | 7 | 2 | 7 | | 0.428 | 0.044 | 0.011 |
| JaxW1 pulse | 7 | 7 | 7 | 7 | 7 | 2 | 7 | 7 | | 0.778 | 3E-04 |
| Sve1 | 7 | 7 | 7 | 7 | 7 | 4 | 6 | 3 | 3 | | 7E-06 |
| Sug1 | 14 | 14 | 14 | 14 | 14 | 8 | 13 | 5 | 5 | 8 | |

**SBP**

| SBP | con | atenolol | iso 1 | iso 10 | Des1 | Sve1 | Gav1 | Sug1 | JaxW1 |
|---|---|---|---|---|---|---|---|---|---|
| con | | 0.802 | 0.742 | 0.722 | 0.239 | 0.047 | 0.401 | 0.629 | 0.388 |
| atenolol | 23 | | 0.685 | 0.712 | 0.311 | 0.1 | 0.737 | 0.553 | 0.292 |
| iso 1 | 23 | 23 | | 0.68 | 0.191 | 0.133 | 0.482 | 0.634 | 0.293 |
| iso 10 | 23 | 23 | 23 | | 0.116 | 0.252 | 0.461 | 0.767 | 0.164 |
| Des1 | 10 | 10 | 10 | 10 | | 0.761 | 0.549 | 0.121 | 0.59 |
| Sve1 | 7 | 7 | 7 | 7 | 7 | | 0.024 | 0.035 | 0.01 |
| Gav1 | 7 | 7 | 7 | 7 | 4 | 5 | | 0.264 | na |
| Sug1 | 14 | 14 | 14 | 14 | 8 | 8 | 9 | | 0.097 |
| JaxW1 | 7 | 7 | 7 | 7 | 5 | 3 | 2 | 5 | |

**ECG_PR**

| ECG_PR | con | atenolol | iso 1 | iso 10 | Ham1 | JaxW1 |
|---|---|---|---|---|---|---|
| con | | 0.903 | 0.685 | 0.614 | 0.041 | 0.011 |
| atenolol | 23 | | 0.662 | 0.612 | 0.054 | 4E-04 |
| iso 1 | 23 | 23 | | 0.689 | 1E-03 | 0.117 |
| iso 10 | 23 | 23 | 23 | | 0.052 | 0.06 |
| Ham1 | 16 | 16 | 16 | 16 | | 0.4 |
| JaxW1 | 7 | 7 | 7 | 7 | 7 | |

**ECG_QRS**

| ECG_QRS | con | atenolol | iso 1 | iso 10 | Ham1 | JaxW1 |
|---|---|---|---|---|---|---|
| con | | 0.564 | 0.382 | 0.152 | 0.078 | 0.246 |
| atenolol | 23 | | 0.422 | 0.145 | 0.38 | 0.464 |
| iso 1 | 23 | 23 | | 0.484 | 0.165 | 0.072 |
| iso 10 | 23 | 23 | 23 | | 0.01 | 0.017 |
| Ham1 | 16 | 16 | 16 | 16 | | 0.314 |
| JaxW1 | 7 | 7 | 7 | 7 | 7 | |

**ECG_QT**

| ECG_QT | con | atenolol | iso 1 | iso 10 | JaxW1 |
|---|---|---|---|---|---|
| con | | 0.611 | 0.112 | 0.306 | 0.19 |
| atenolol | 23 | | 0.041 | 0.19 | 0.167 |
| iso 1 | 23 | 23 | | 0.405 | 0.159 |
| iso 10 | 23 | 23 | 23 | | 0.743 |
| JaxW1 | 7 | 7 | 7 | 7 | |

**ECG_RR**

| ECG_RR | con | atenolol | iso 1 | iso 10 | Ham1 | JaxW1 |
|---|---|---|---|---|---|---|
| con | | 0.627 | 0.55 | 0.363 | 0.052 | 0.027 |
| atenolol | 23 | | 0.28 | 0.187 | 0.007 | 0.206 |
| iso 1 | 23 | 23 | | 0.636 | 0.106 | 0.24 |
| iso 10 | 23 | 23 | 23 | | 0.154 | 0.275 |
| Ham1 | 16 | 16 | 16 | 16 | | 0.488 |
| JaxW1 | 7 | 7 | 7 | 7 | 7 | |

**ECG_ST**

| ECG_ST | con | atenolol | iso 1 | iso 10 | JaxW1 |
|---|---|---|---|---|---|
| con | | 0.648 | 0.072 | 0.305 | 0.039 |
| atenolol | 23 | | 0.004 | 0.128 | 0.2 |
| iso 1 | 23 | 23 | | 0.296 | 0.461 |
| iso 10 | 23 | 23 | 23 | | 0.907 |
| JaxW1 | 7 | 7 | 7 | 7 | |

**ECG_QTc**

| ECG_QTc | con | atenolol | iso 1 | iso 10 | JaxW1 |
|---|---|---|---|---|---|
| con | | 0.556 | 0.161 | 0.319 | 0.133 |
| atenolol | 23 | | 0.141 | 0.126 | 0.025 |
| iso 1 | 23 | 23 | | 0.339 | 0.383 |
| iso 10 | 23 | 23 | 23 | | 0.672 |
| JaxW1 | 7 | 7 | 7 | 7 | |

Suppl. Figure 4 part 1

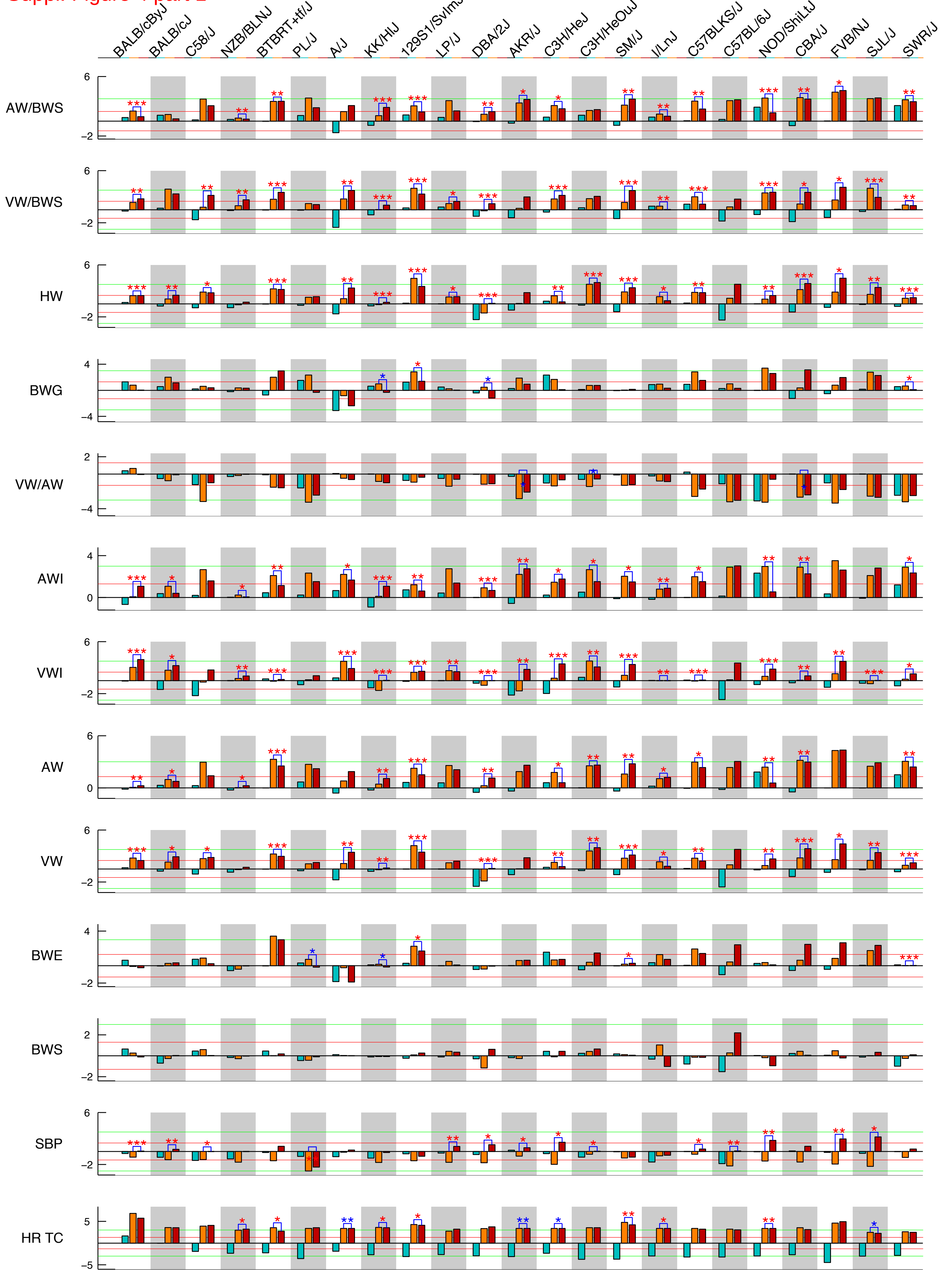

Suppl. Figure 4 part 2

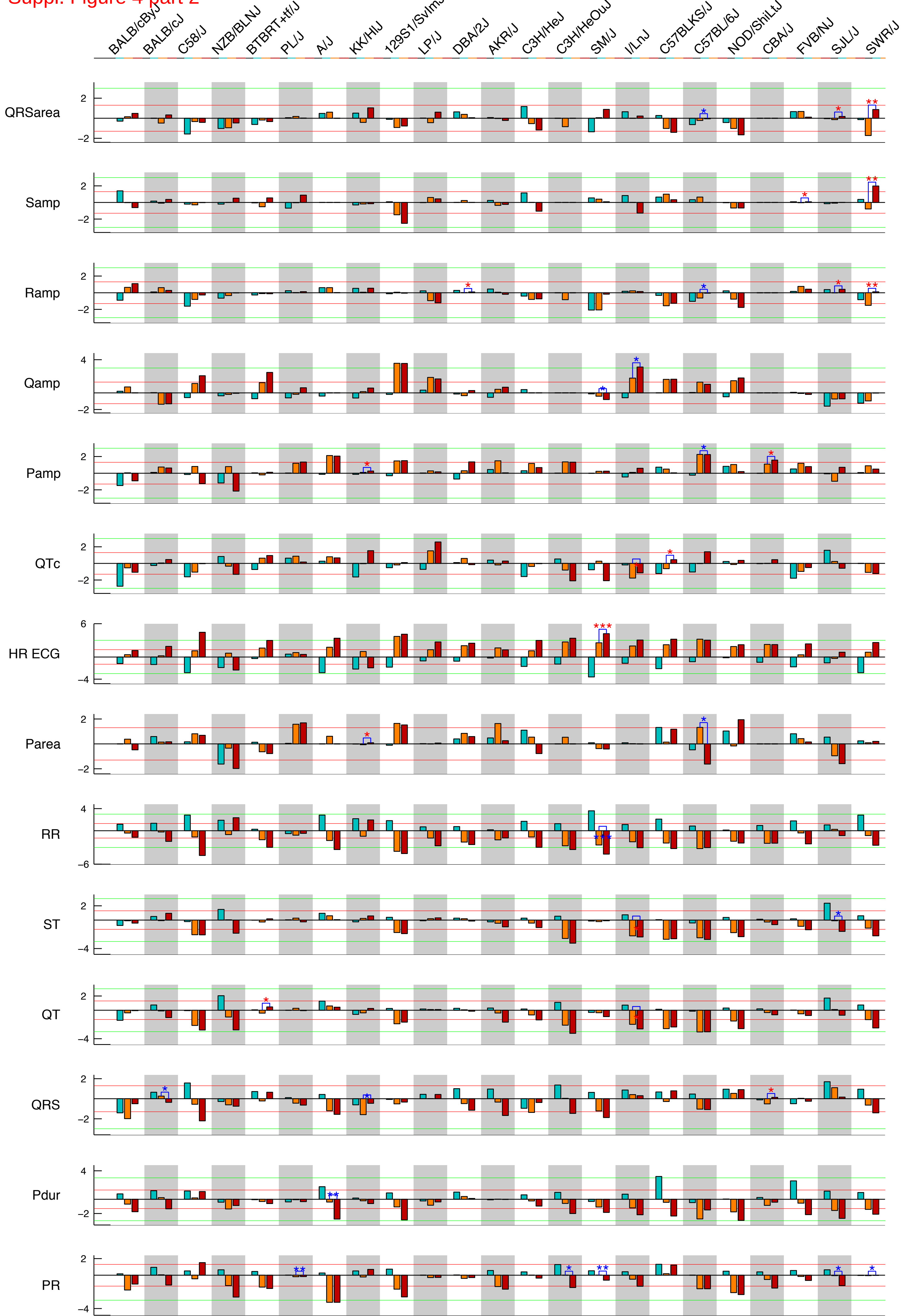

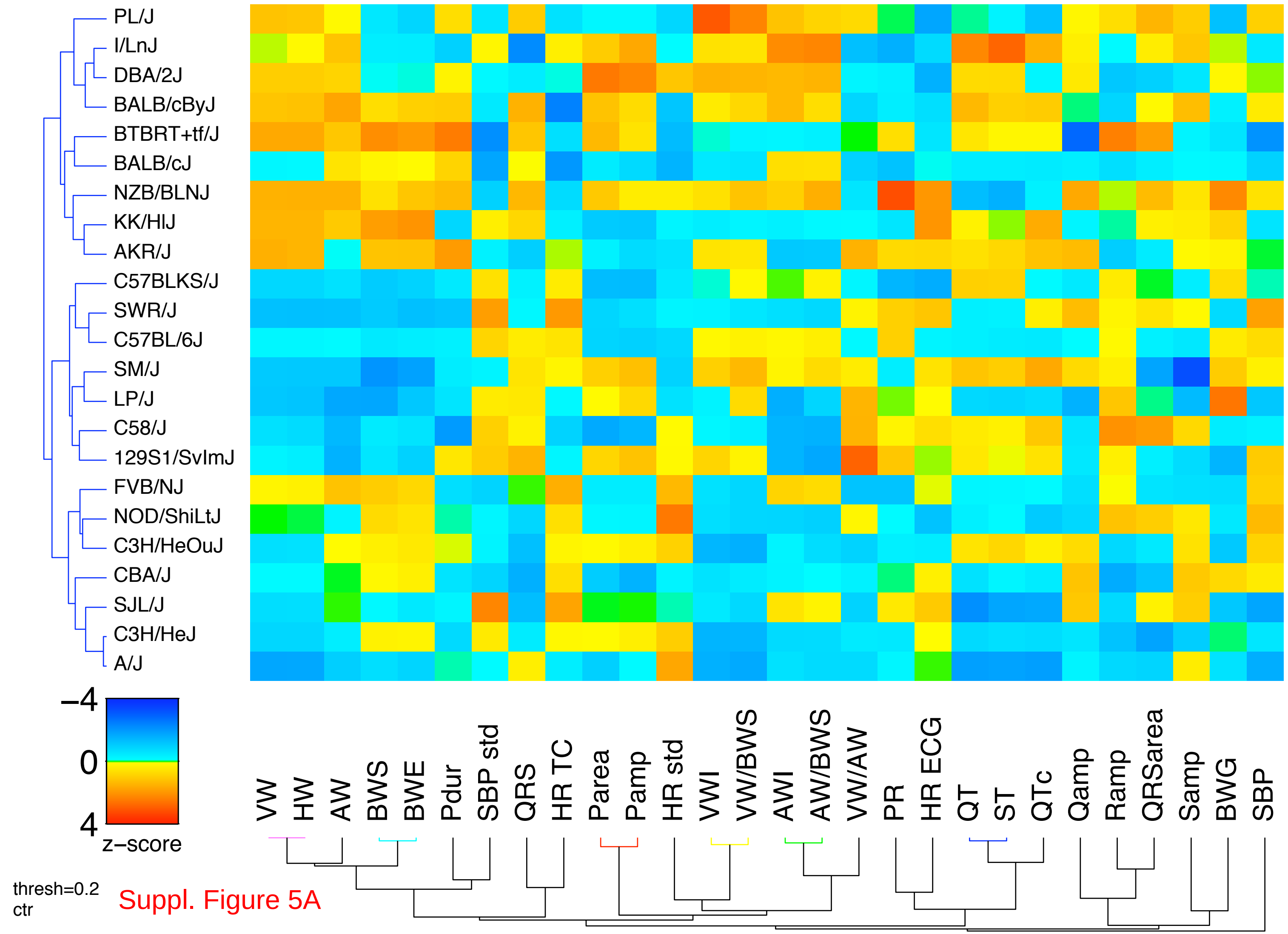


Suppl. Figure 5A

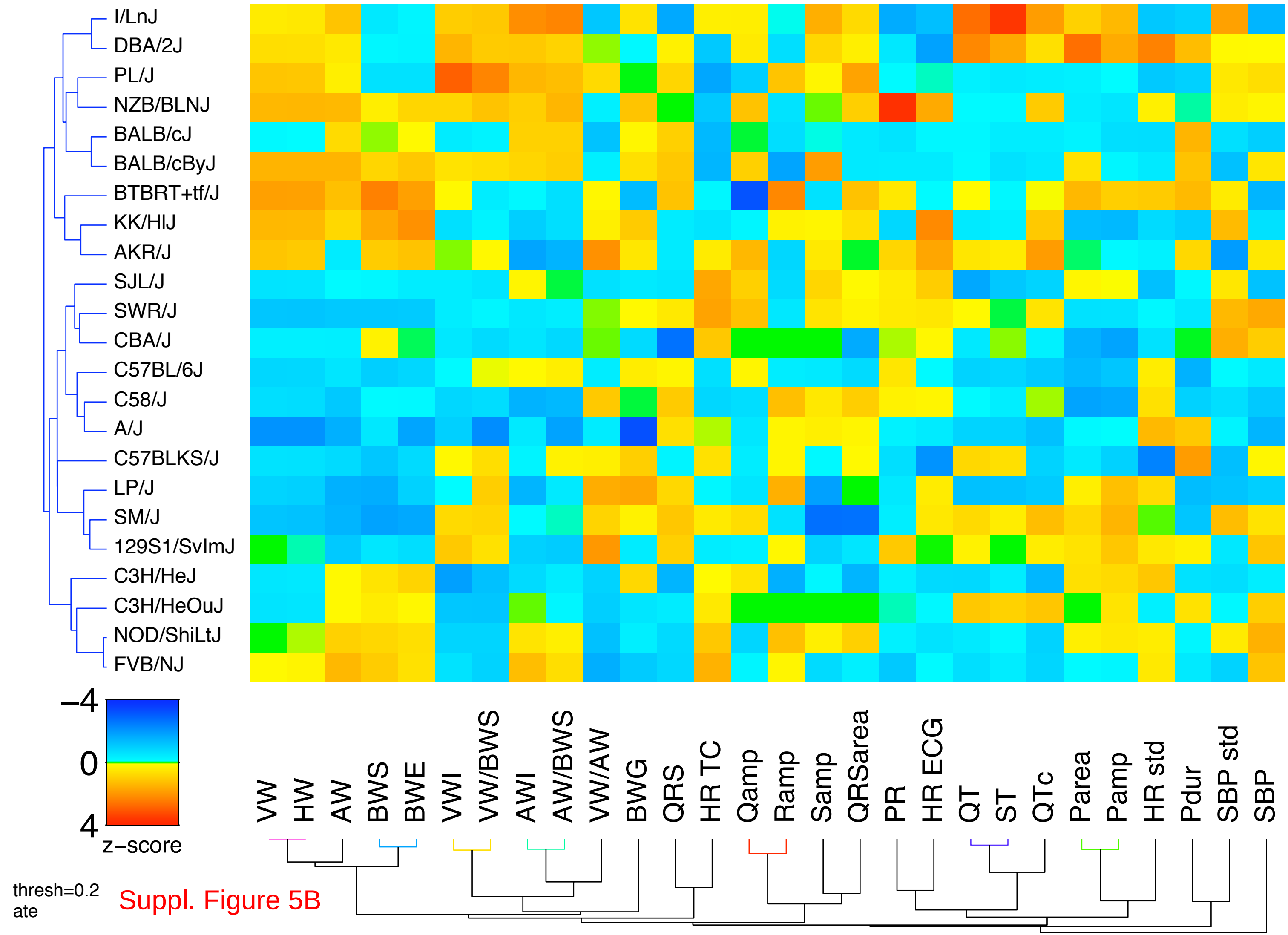


thresh=0.2
ate

Suppl. Figure 5B

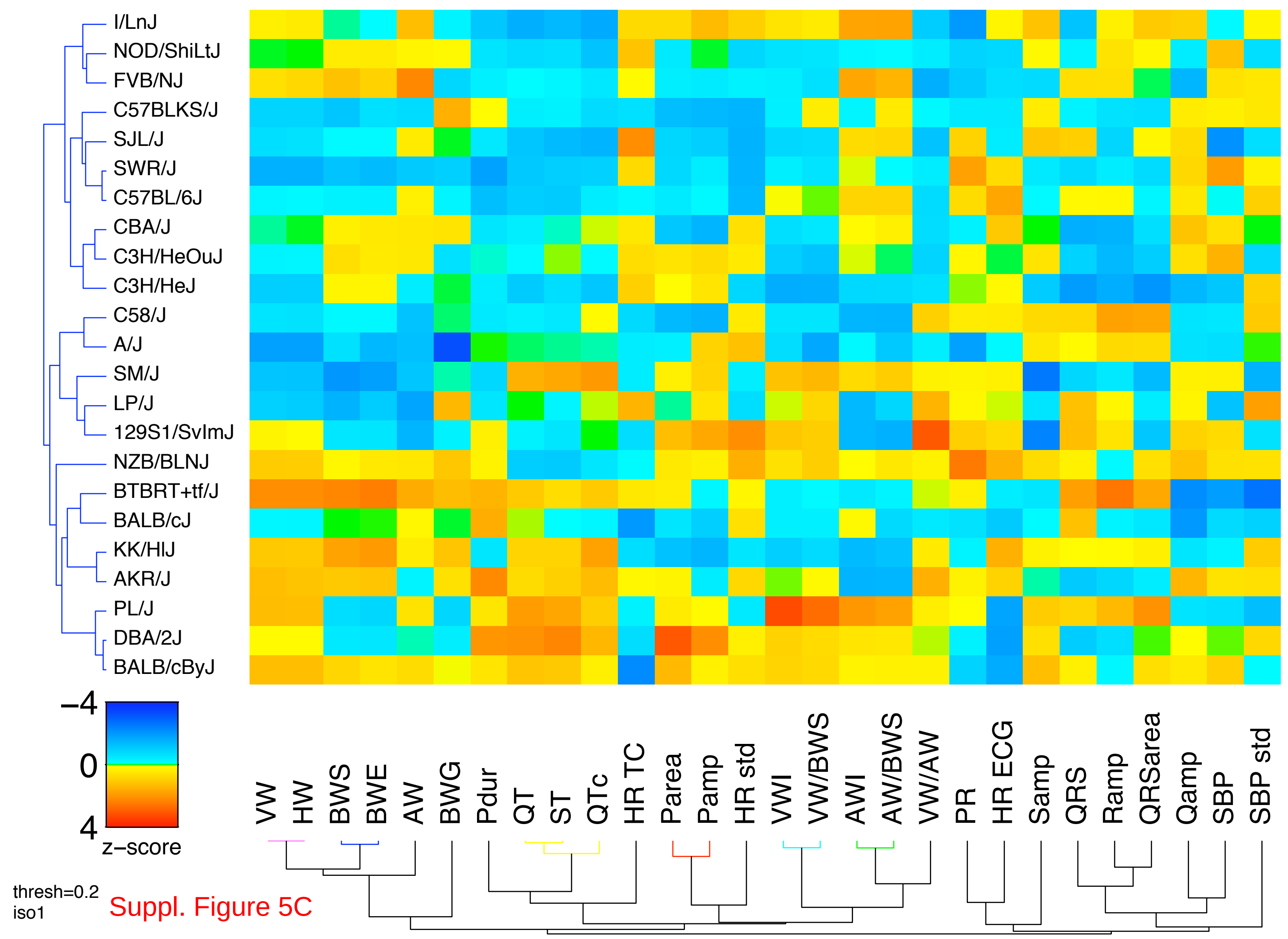

Suppl. Figure 5C

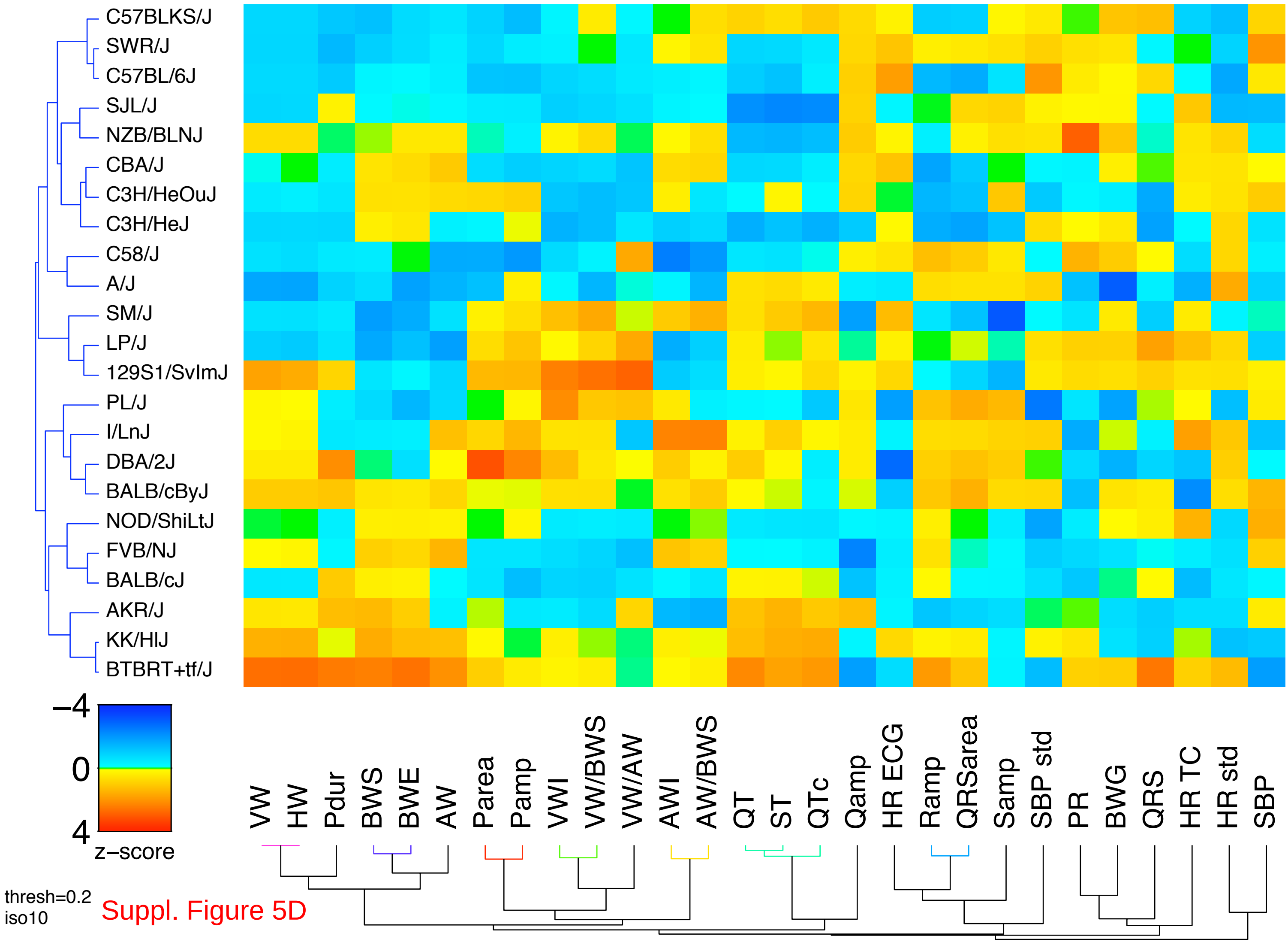

Suppl. Figure 5D

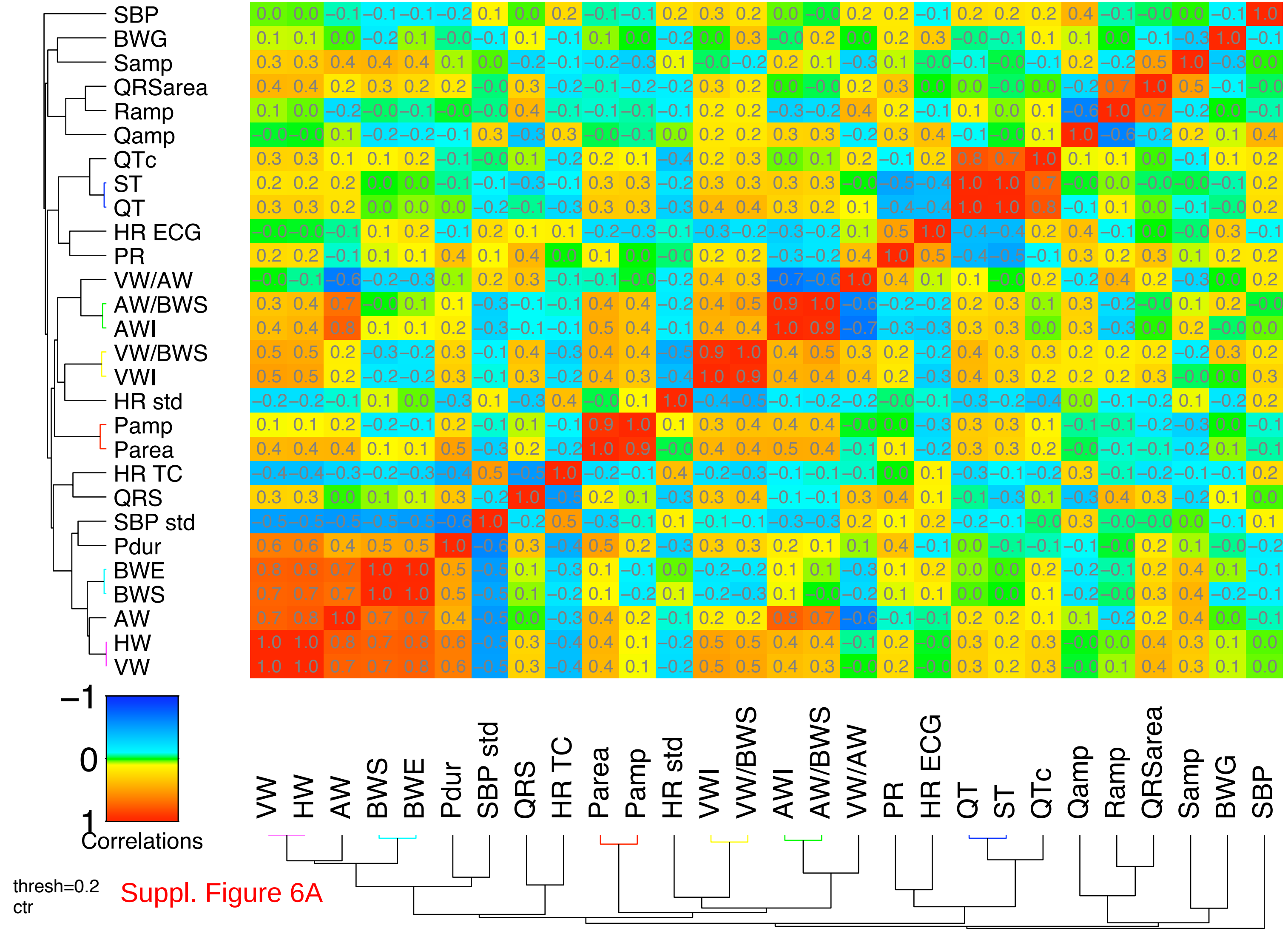


Suppl. Figure 6A

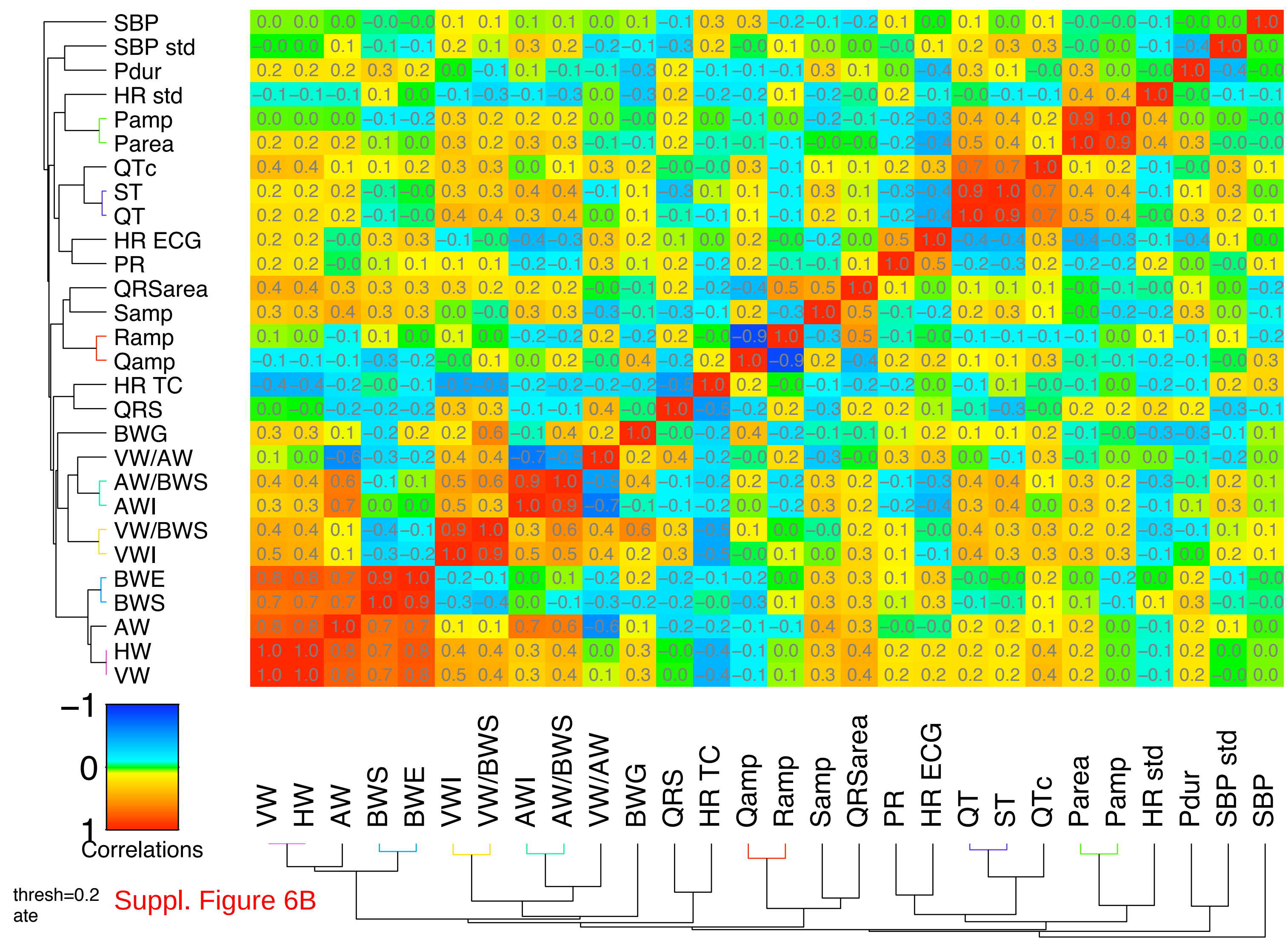


Suppl. Figure 6B

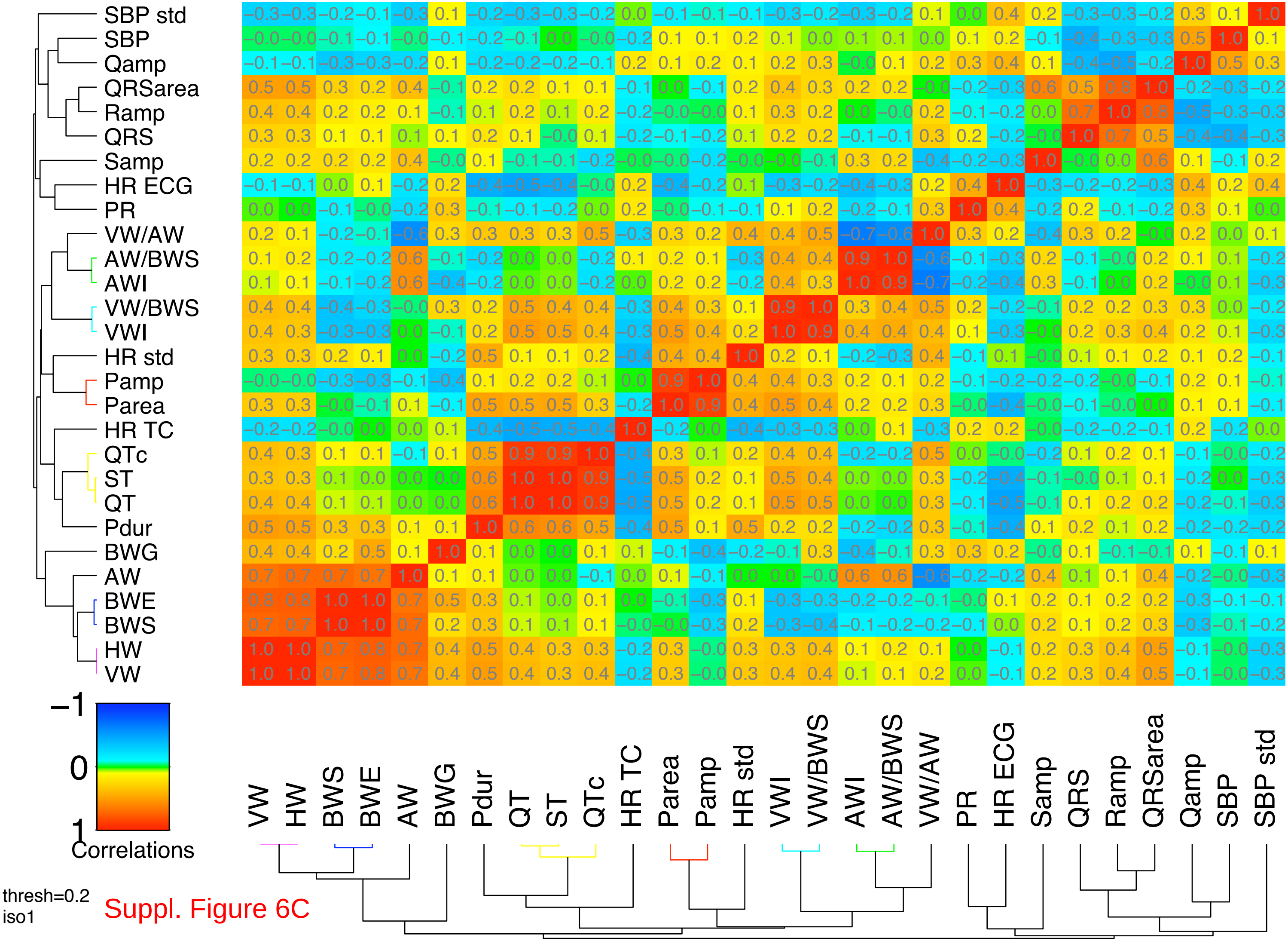


Suppl. Figure 6C

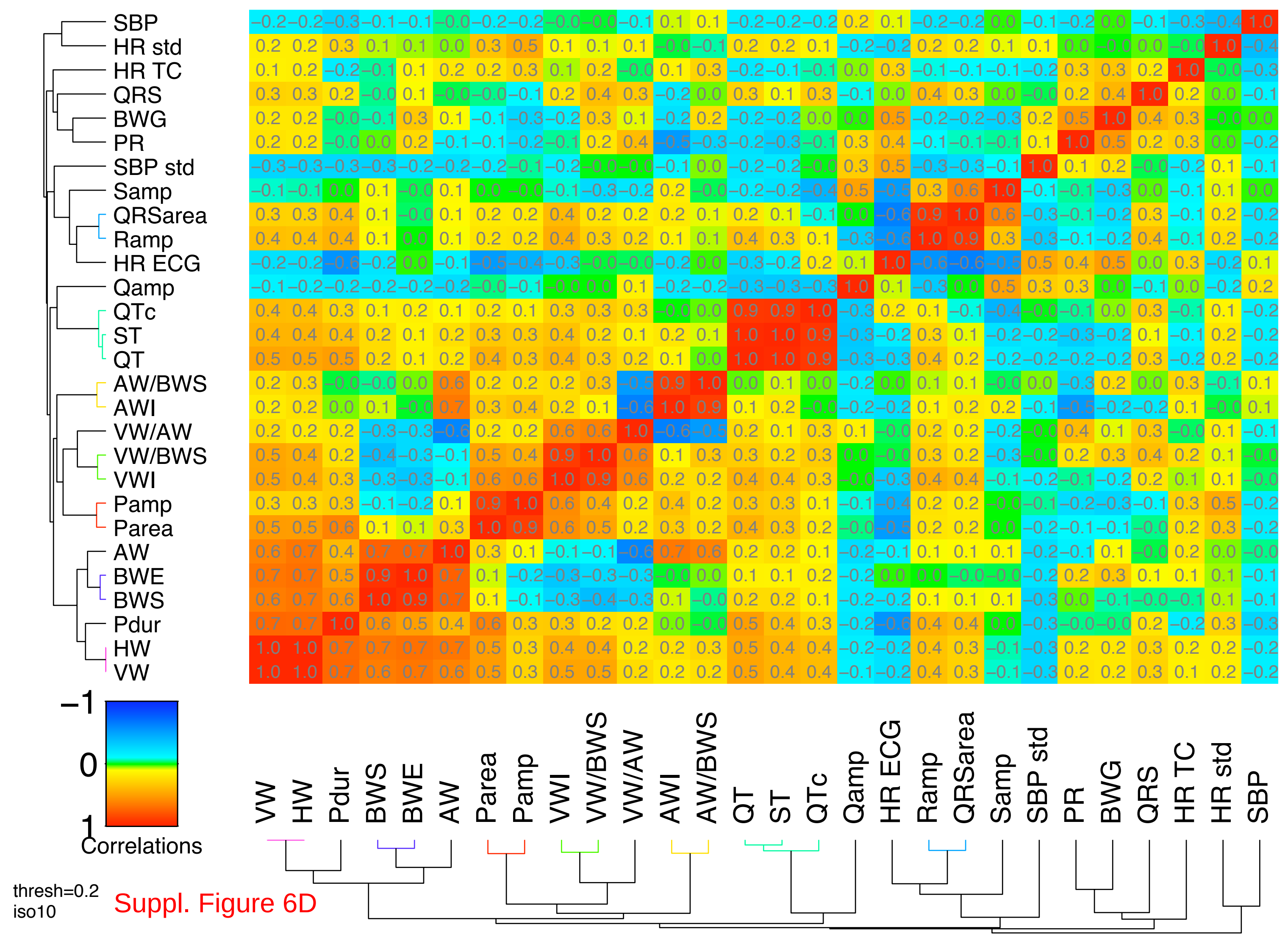


Suppl. Figure 6D

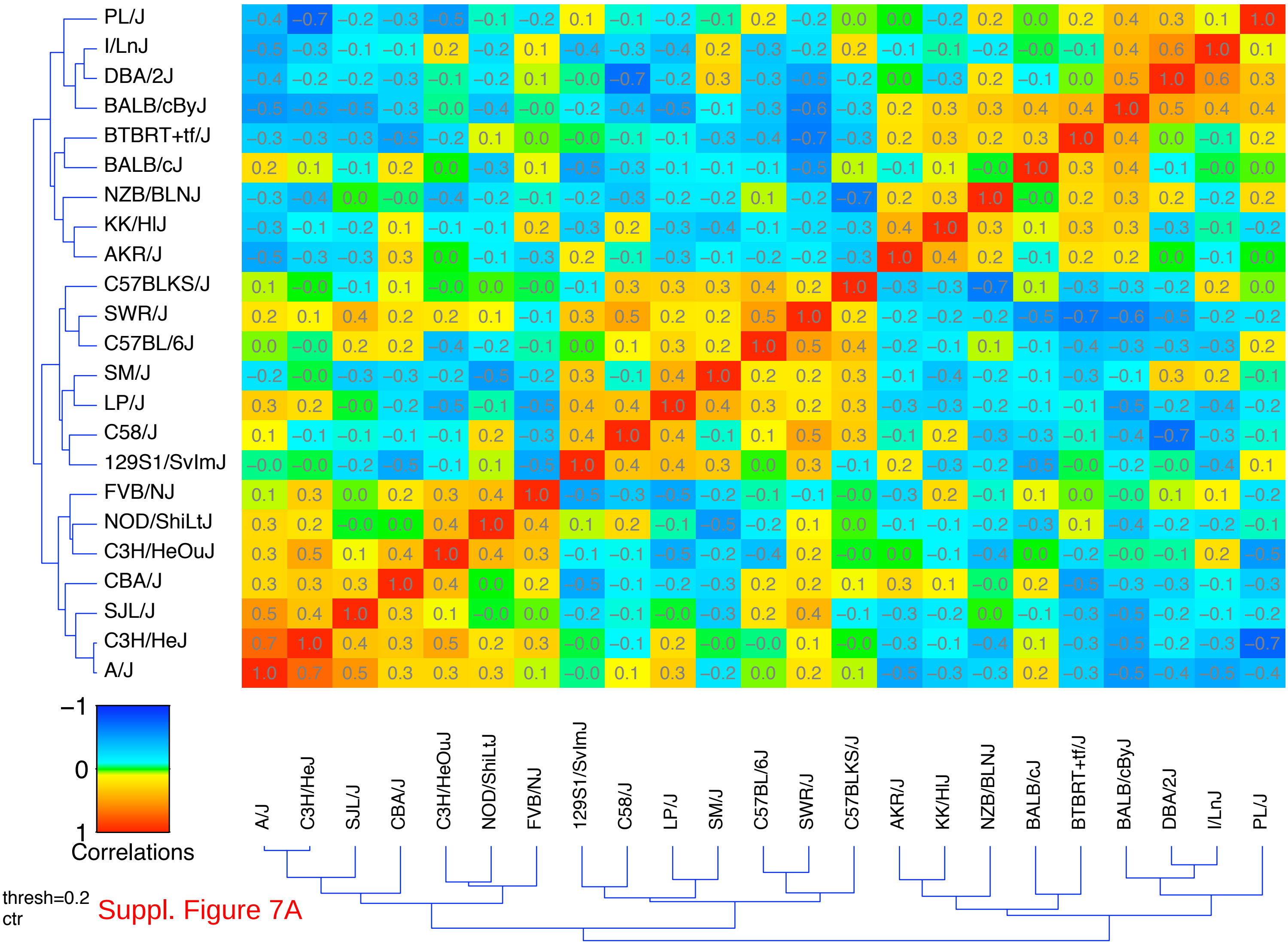


| | A/J | C3H/HeJ | SJL/J | CBA/J | C3H/HeOuJ | NOD/ShiLtJ | FVB/NJ | 129S1/SvImJ | C58/J | LP/J | SM/J | C57BL/6J | SWR/J | C57BLKS/J | AKR/J | KK/HlJ | NZB/BLNJ | BALB/cJ | BTBRT+tf/J | BALB/cByJ | DBA/2J | I/LnJ | PL/J |
|---|---|---|---|---|---|---|---|---|---|---|---|---|---|---|---|---|---|---|---|---|---|---|---|
| PL/J | −0.4 | −0.7 | −0.2 | −0.3 | −0.5 | −0.1 | −0.2 | 0.1 | −0.1 | −0.2 | −0.1 | 0.2 | −0.2 | 0.0 | 0.0 | −0.2 | 0.2 | 0.0 | 0.2 | 0.4 | 0.3 | 0.1 | 1.0 |
| I/LnJ | −0.5 | −0.3 | −0.1 | −0.1 | 0.2 | −0.2 | 0.1 | −0.4 | −0.3 | −0.4 | 0.2 | −0.3 | −0.2 | 0.2 | −0.1 | −0.1 | −0.2 | −0.0 | −0.1 | 0.4 | 0.6 | 1.0 | 0.1 |
| DBA/2J | −0.4 | −0.2 | −0.2 | −0.3 | −0.1 | −0.2 | 0.1 | −0.0 | −0.7 | −0.2 | 0.3 | −0.3 | −0.5 | −0.2 | 0.0 | −0.3 | 0.2 | −0.1 | 0.0 | 0.5 | 1.0 | 0.6 | 0.3 |
| BALB/cByJ | −0.5 | −0.5 | −0.5 | −0.3 | −0.0 | −0.4 | −0.0 | −0.2 | −0.4 | −0.5 | −0.1 | −0.3 | −0.6 | −0.3 | 0.2 | 0.3 | 0.3 | 0.4 | 0.4 | 1.0 | 0.5 | 0.4 | 0.4 |
| BTBRT+tf/J | −0.3 | −0.3 | −0.3 | −0.5 | −0.2 | 0.1 | 0.0 | −0.0 | −0.1 | −0.1 | −0.3 | −0.4 | −0.7 | −0.3 | 0.2 | 0.3 | 0.2 | 0.3 | 1.0 | 0.4 | 0.0 | −0.1 | 0.2 |
| BALB/cJ | 0.2 | 0.1 | −0.1 | 0.2 | 0.0 | −0.3 | 0.1 | −0.5 | −0.3 | −0.1 | −0.1 | −0.1 | −0.5 | 0.1 | −0.1 | 0.1 | −0.0 | 1.0 | 0.3 | 0.4 | −0.1 | −0.0 | 0.0 |
| NZB/BLNJ | −0.3 | −0.4 | 0.0 | −0.0 | −0.4 | −0.2 | −0.1 | −0.2 | −0.3 | −0.2 | −0.2 | 0.1 | −0.2 | −0.7 | 0.2 | 0.3 | 1.0 | −0.0 | 0.2 | 0.3 | 0.2 | −0.2 | 0.2 |
| KK/HlJ | −0.3 | −0.1 | −0.2 | 0.1 | −0.1 | −0.1 | 0.2 | −0.3 | 0.2 | −0.3 | −0.4 | −0.1 | −0.2 | −0.3 | 0.4 | 1.0 | 0.3 | 0.1 | 0.3 | 0.3 | −0.3 | −0.1 | −0.2 |
| AKR/J | −0.5 | −0.3 | −0.3 | 0.3 | 0.0 | −0.1 | −0.3 | 0.2 | −0.1 | −0.3 | −0.1 | −0.2 | −0.2 | −0.3 | 1.0 | 0.4 | 0.2 | −0.1 | 0.2 | 0.2 | 0.0 | −0.1 | 0.0 |
| C57BLKS/J | 0.1 | −0.0 | −0.1 | 0.1 | −0.0 | 0.0 | −0.0 | −0.1 | 0.3 | 0.3 | 0.3 | 0.4 | 0.2 | 1.0 | −0.3 | −0.3 | −0.7 | 0.1 | −0.3 | −0.3 | −0.2 | 0.2 | 0.0 |
| SWR/J | 0.2 | 0.1 | 0.4 | 0.2 | 0.2 | 0.1 | −0.1 | 0.3 | 0.5 | 0.2 | 0.2 | 0.5 | 1.0 | 0.2 | −0.2 | −0.2 | −0.2 | −0.5 | −0.7 | −0.6 | −0.5 | −0.2 | −0.2 |
| C57BL/6J | 0.0 | −0.0 | 0.2 | 0.2 | −0.4 | −0.2 | −0.1 | 0.0 | 0.1 | 0.3 | 0.2 | 1.0 | 0.5 | 0.4 | −0.2 | −0.1 | 0.1 | −0.1 | −0.4 | −0.3 | −0.3 | −0.3 | 0.2 |
| SM/J | −0.2 | −0.0 | −0.3 | −0.3 | −0.2 | −0.5 | −0.2 | 0.3 | −0.1 | 0.4 | 1.0 | 0.2 | 0.2 | 0.3 | −0.1 | −0.4 | −0.2 | −0.1 | −0.3 | −0.1 | 0.3 | 0.2 | −0.1 |
| LP/J | 0.3 | 0.2 | −0.0 | −0.2 | −0.5 | −0.1 | −0.5 | 0.4 | 0.4 | 1.0 | 0.4 | 0.3 | 0.2 | 0.3 | −0.3 | −0.3 | −0.2 | −0.1 | −0.1 | −0.5 | −0.2 | −0.4 | −0.2 |
| C58/J | 0.1 | −0.1 | −0.1 | −0.1 | −0.1 | 0.2 | −0.3 | 0.4 | 1.0 | 0.4 | −0.1 | 0.1 | 0.5 | 0.3 | −0.1 | 0.2 | −0.3 | −0.3 | −0.1 | −0.4 | −0.7 | −0.3 | −0.1 |
| 129S1/SvImJ | −0.0 | −0.0 | −0.2 | −0.5 | −0.1 | 0.1 | −0.5 | 1.0 | 0.4 | 0.4 | 0.3 | 0.0 | 0.3 | −0.1 | 0.2 | −0.3 | −0.2 | −0.5 | −0.0 | −0.2 | −0.0 | −0.4 | 0.1 |
| FVB/NJ | 0.1 | 0.3 | 0.0 | 0.2 | 0.3 | 0.4 | 1.0 | −0.5 | −0.3 | −0.5 | −0.2 | −0.1 | −0.1 | −0.0 | −0.3 | 0.2 | −0.1 | 0.1 | 0.0 | −0.0 | 0.1 | 0.1 | −0.2 |
| NOD/ShiLtJ | 0.3 | 0.2 | −0.0 | 0.0 | 0.4 | 1.0 | 0.4 | 0.1 | 0.2 | −0.1 | −0.5 | −0.2 | 0.1 | 0.0 | −0.1 | −0.1 | −0.2 | −0.3 | 0.1 | −0.4 | −0.2 | −0.2 | −0.1 |
| C3H/HeOuJ | 0.3 | 0.5 | 0.1 | 0.4 | 1.0 | 0.4 | 0.3 | −0.1 | −0.1 | −0.5 | −0.2 | −0.4 | 0.2 | −0.0 | 0.0 | −0.1 | −0.4 | 0.0 | −0.2 | −0.0 | −0.1 | 0.2 | −0.5 |
| CBA/J | 0.3 | 0.3 | 0.3 | 1.0 | 0.4 | 0.0 | 0.2 | −0.5 | −0.1 | −0.2 | −0.3 | 0.2 | 0.2 | 0.1 | 0.3 | 0.1 | −0.0 | 0.2 | −0.5 | −0.3 | −0.3 | −0.1 | −0.3 |
| SJL/J | 0.5 | 0.4 | 1.0 | 0.3 | 0.1 | −0.0 | 0.0 | −0.2 | −0.1 | −0.0 | −0.3 | 0.2 | 0.4 | −0.1 | −0.3 | −0.2 | 0.0 | −0.1 | −0.3 | −0.5 | −0.2 | −0.1 | −0.2 |
| C3H/HeJ | 0.7 | 1.0 | 0.4 | 0.3 | 0.5 | 0.2 | 0.3 | −0.0 | −0.1 | 0.2 | −0.0 | −0.0 | 0.1 | −0.0 | −0.3 | −0.1 | −0.4 | 0.1 | −0.3 | −0.5 | −0.2 | −0.3 | −0.7 |
| A/J | 1.0 | 0.7 | 0.5 | 0.3 | 0.3 | 0.3 | 0.1 | −0.0 | 0.1 | 0.3 | −0.2 | 0.0 | 0.2 | 0.1 | −0.5 | −0.3 | −0.3 | 0.2 | −0.3 | −0.5 | −0.4 | −0.5 | −0.4 |

Suppl. Figure 7A

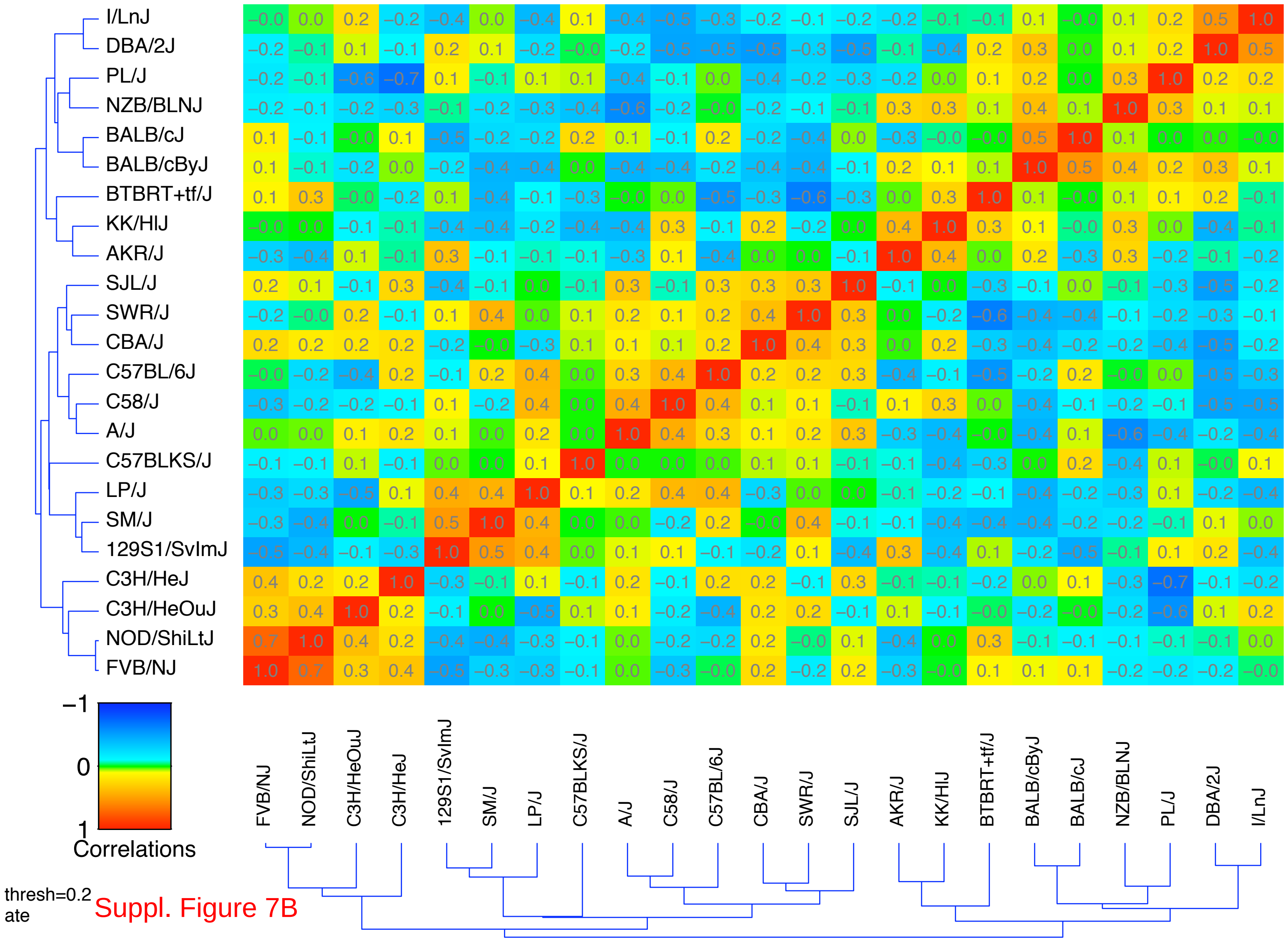


Suppl. Figure 7B

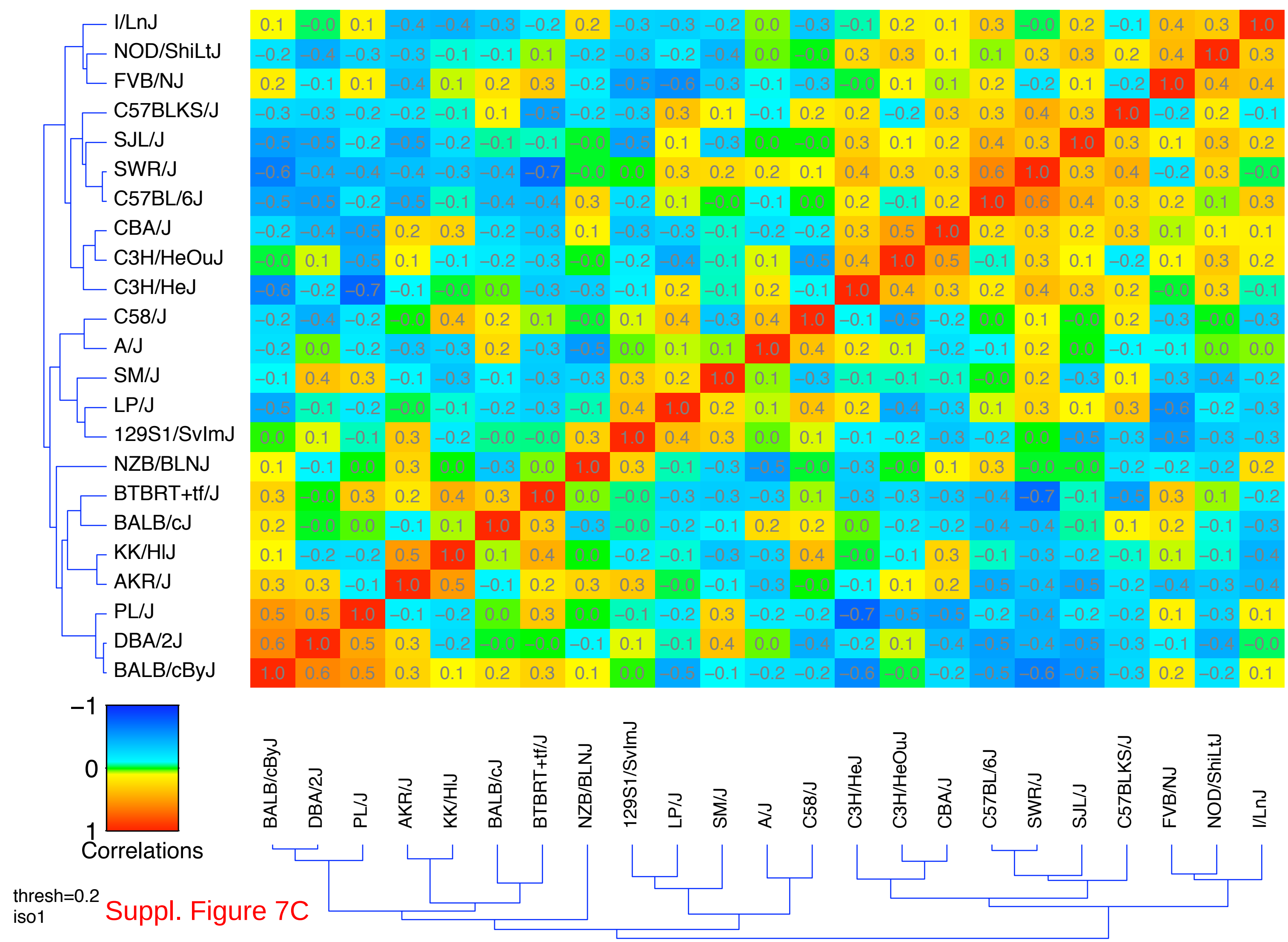


Suppl. Figure 7C

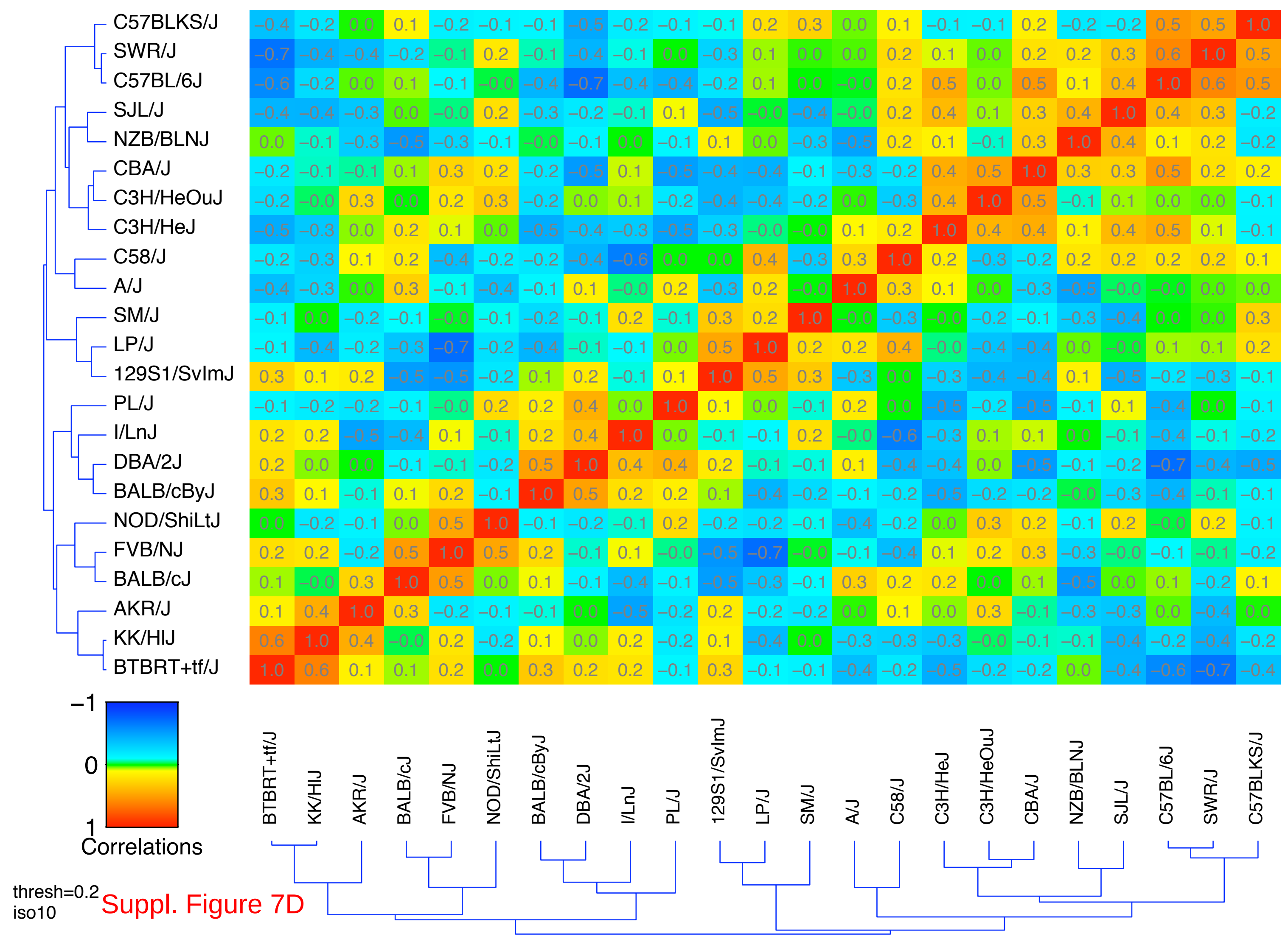

Suppl. Figure 7D

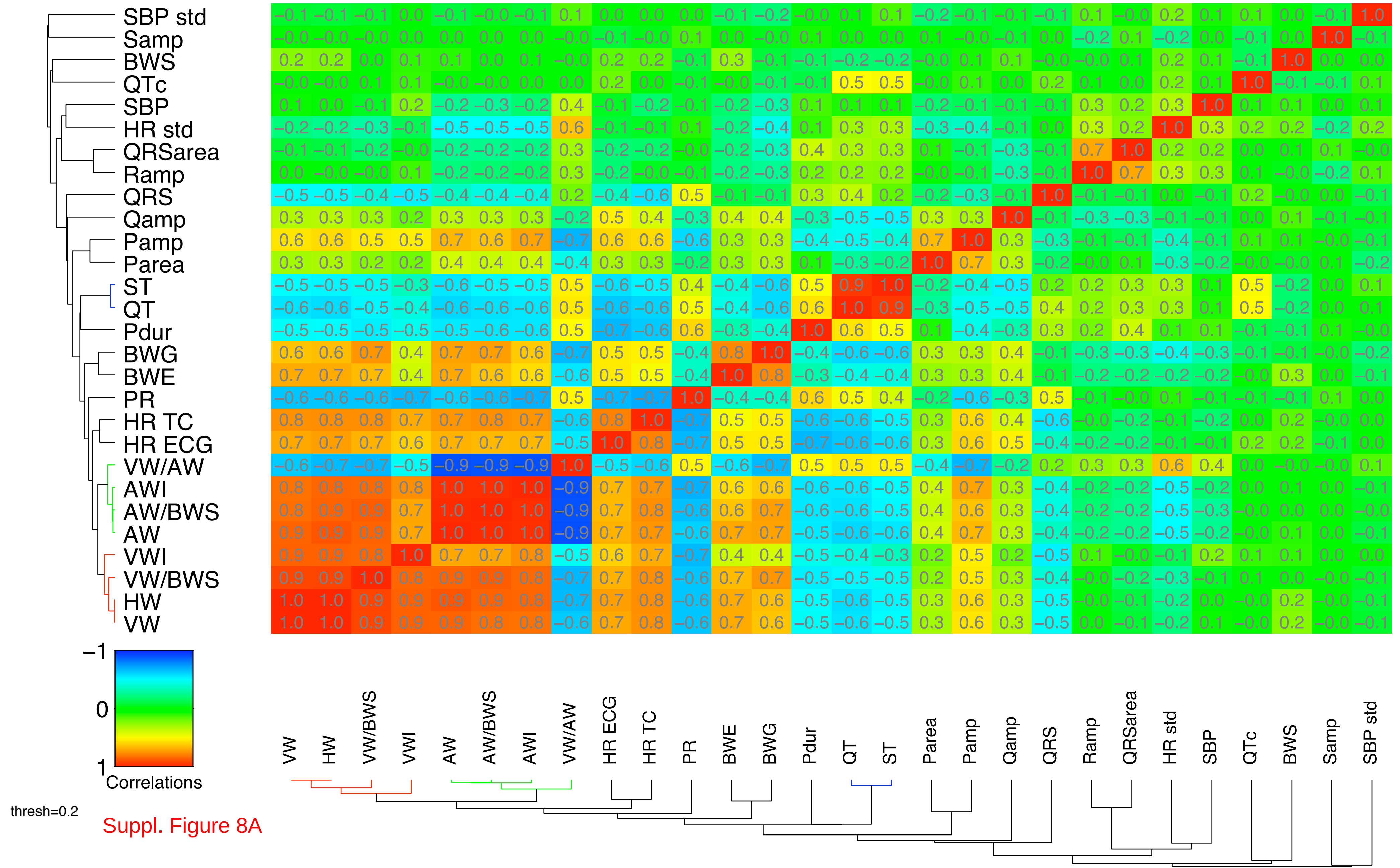


Suppl. Figure 8A

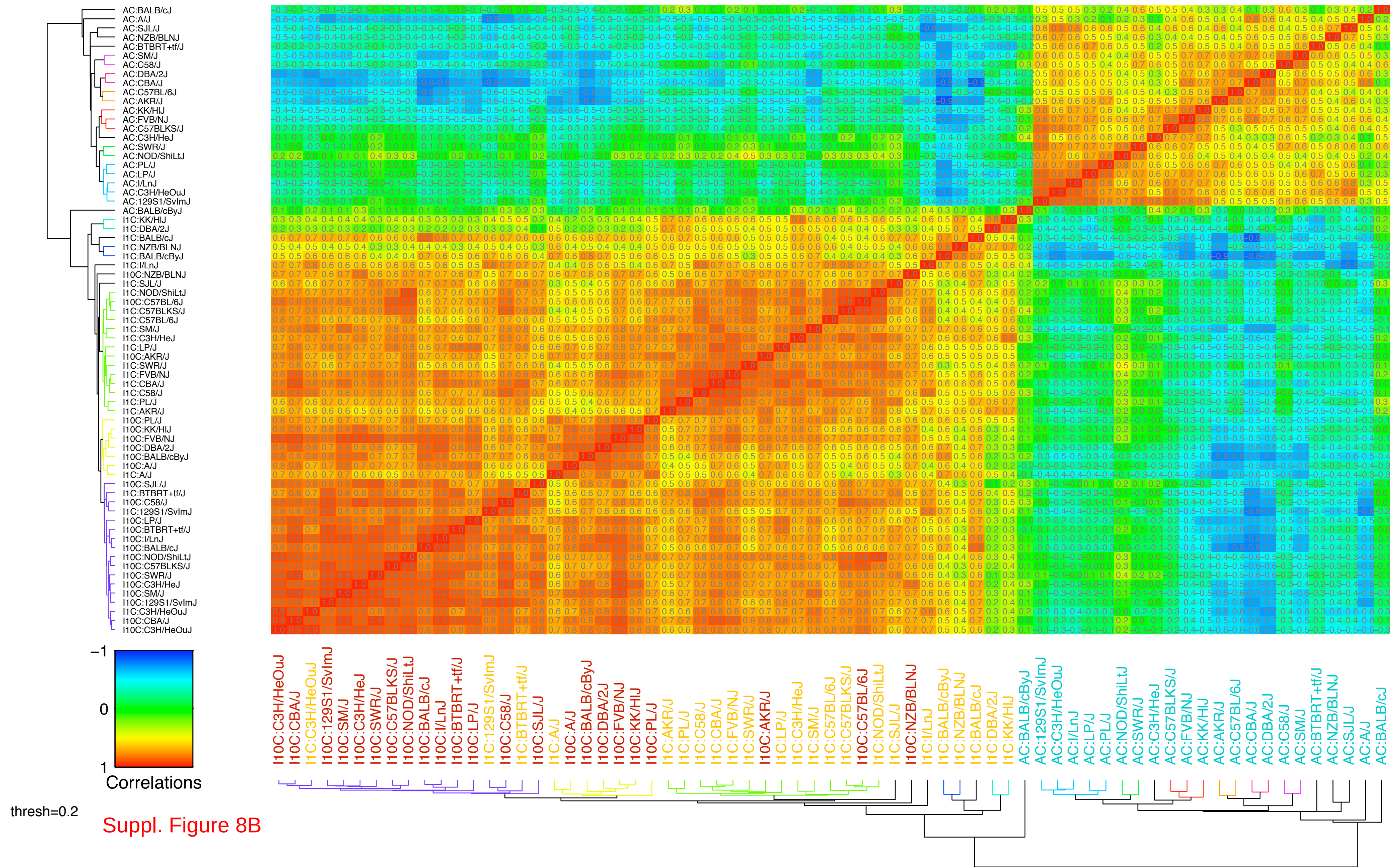


Suppl. Figure 8B

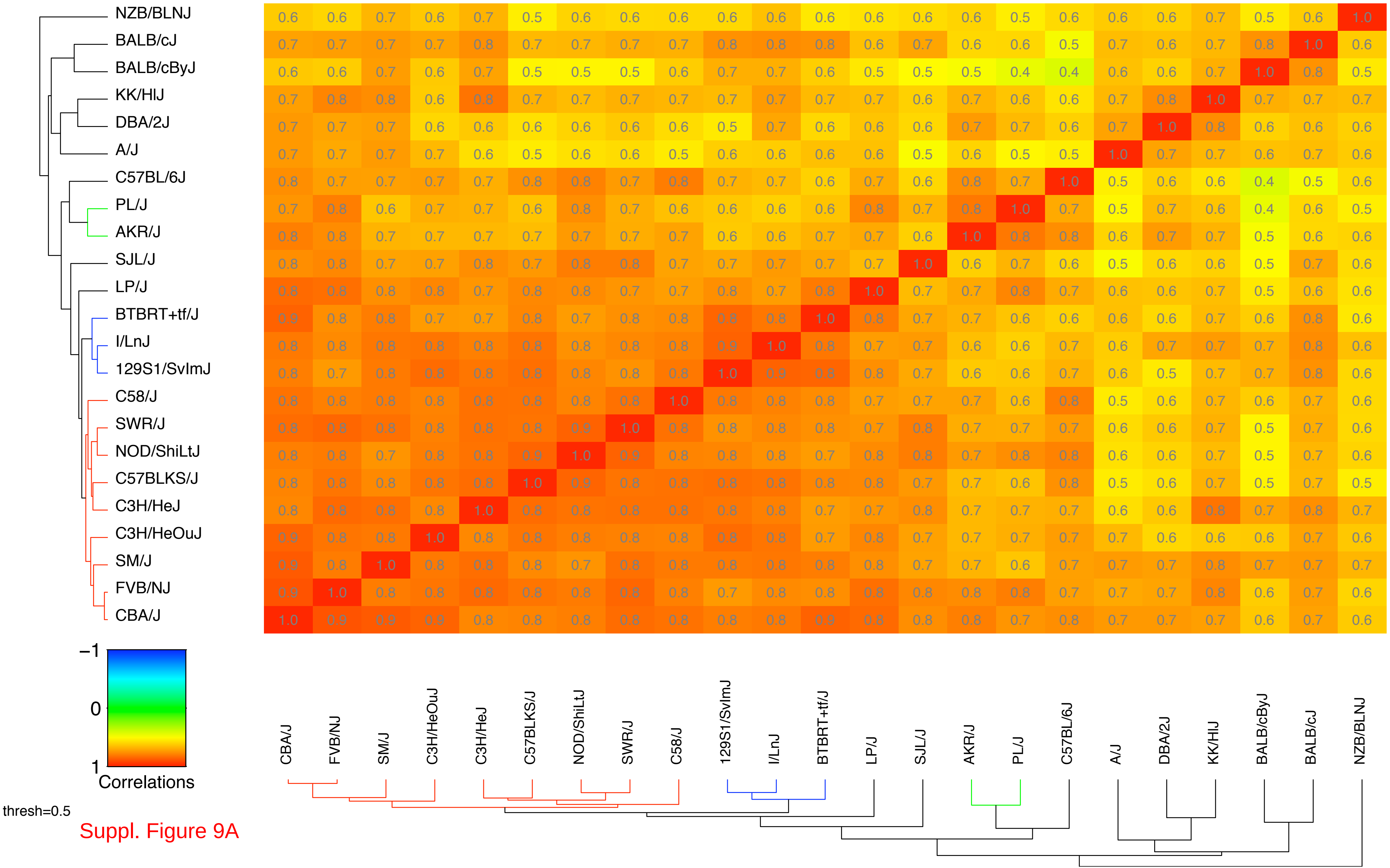


Suppl. Figure 9A

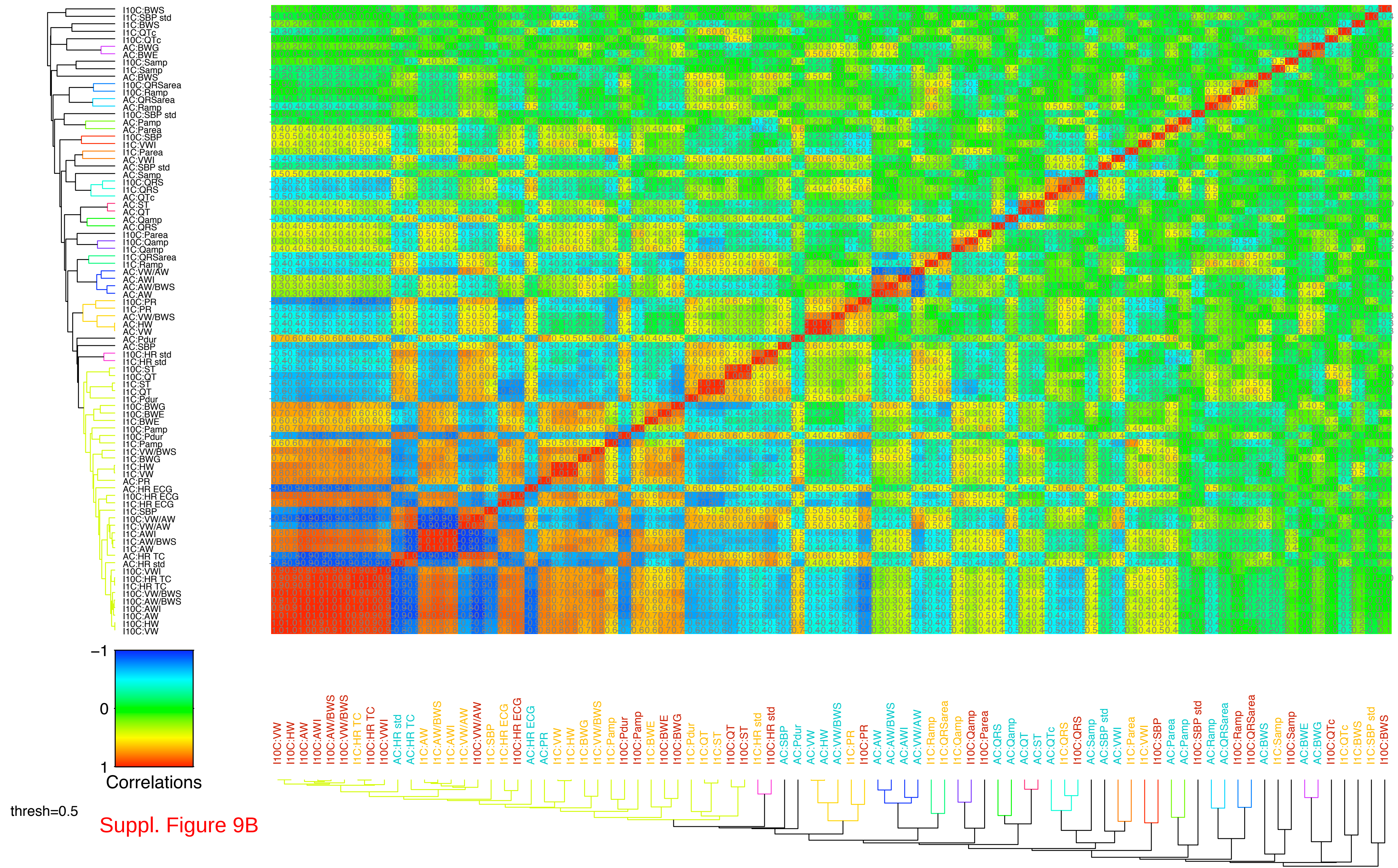


Suppl. Figure 9B

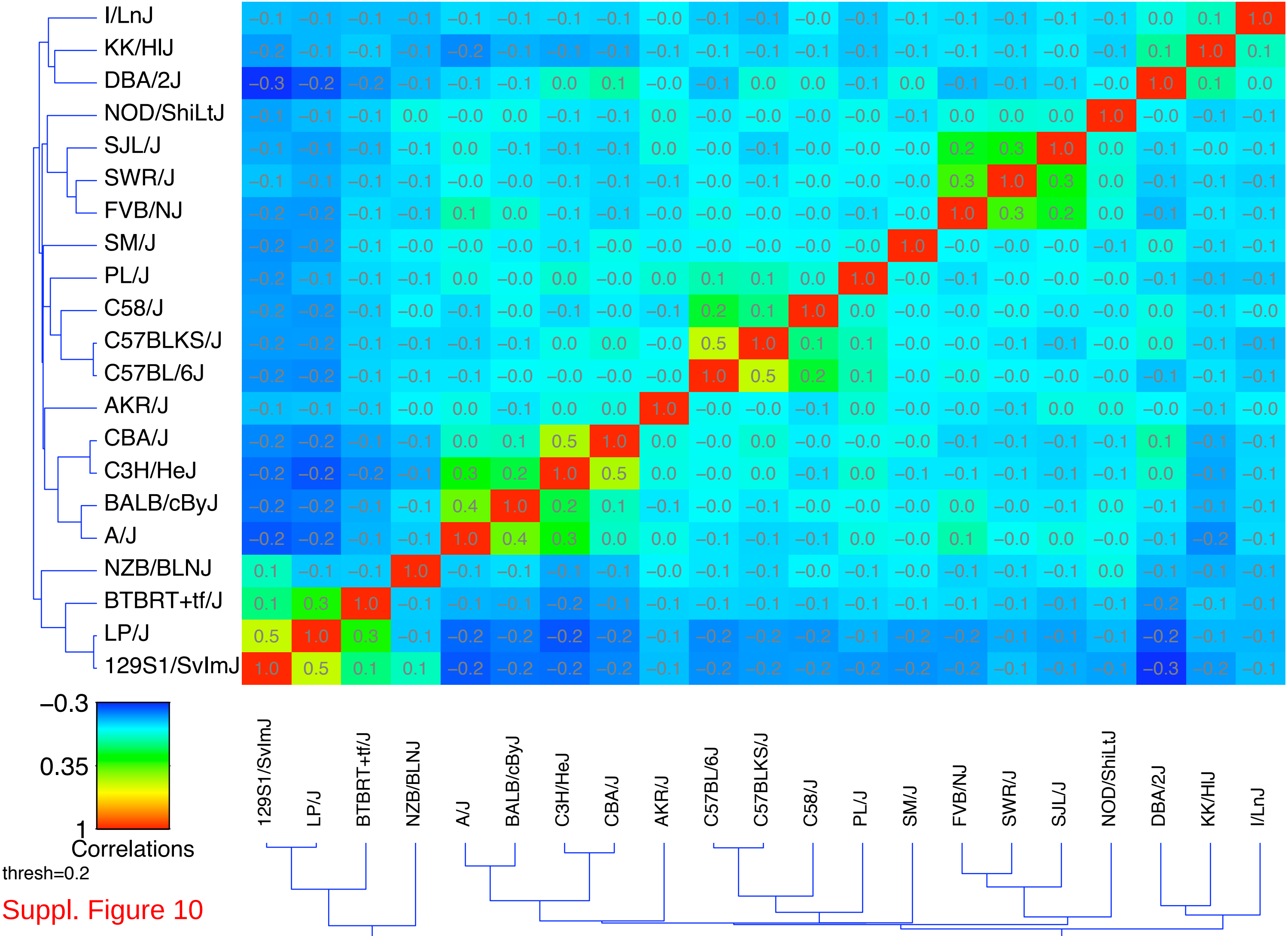


Suppl. Figure 10

Suppl. Figure 11

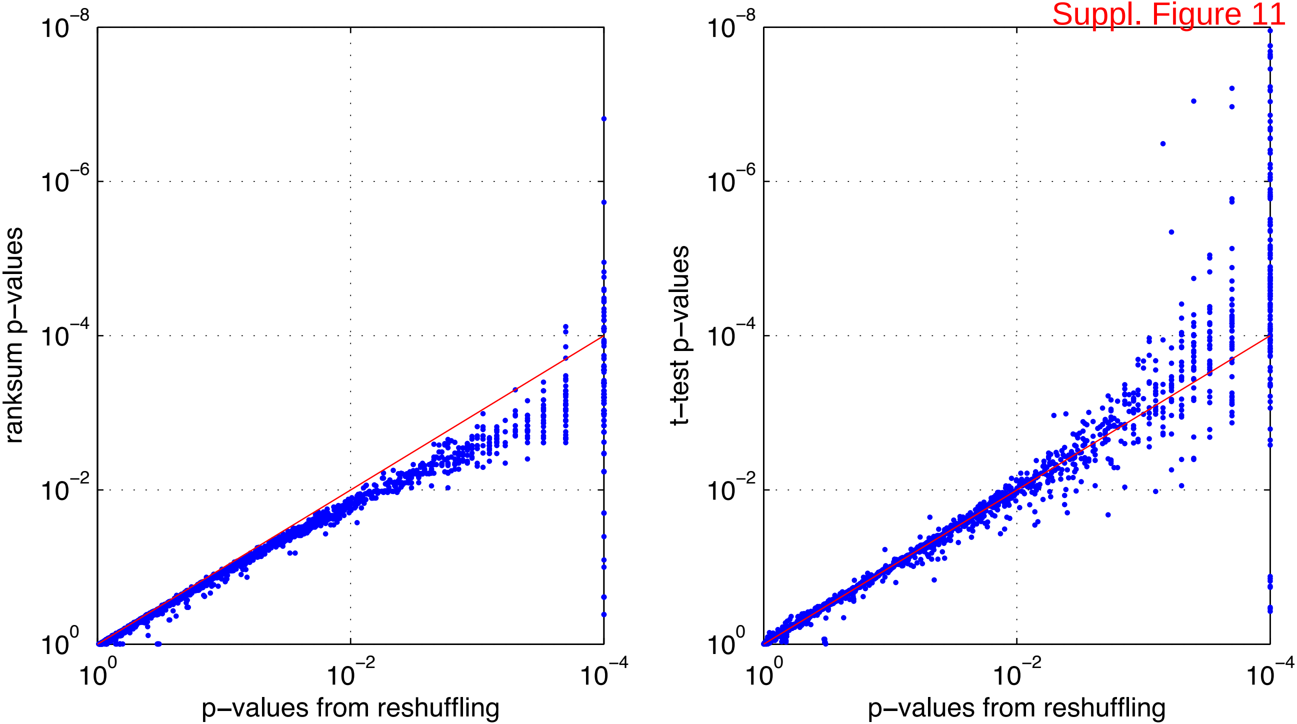

Suppl. Figure 12 part 1

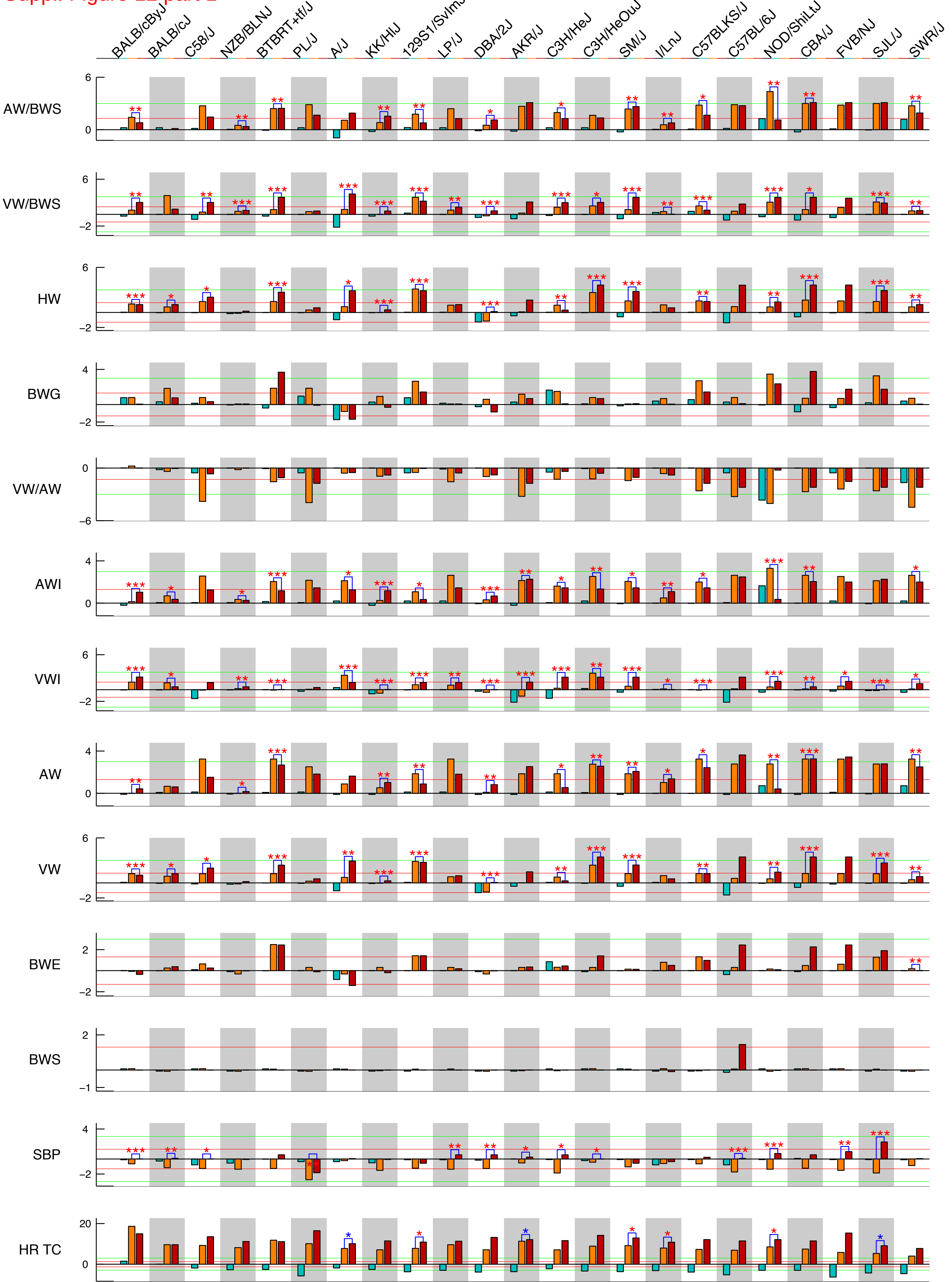

Suppl. Figure 12 part 2

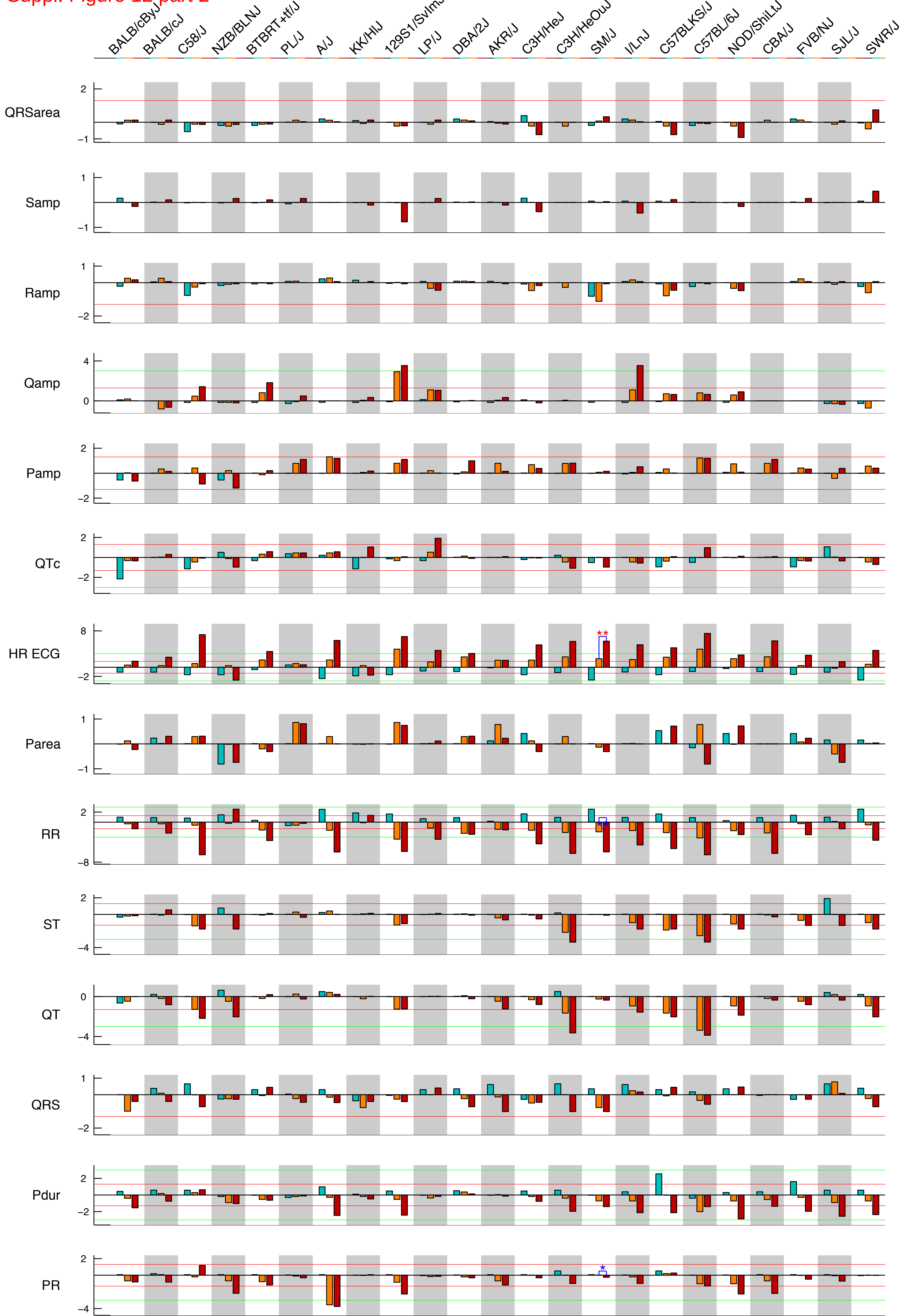

Suppl. Figure 13 part 1

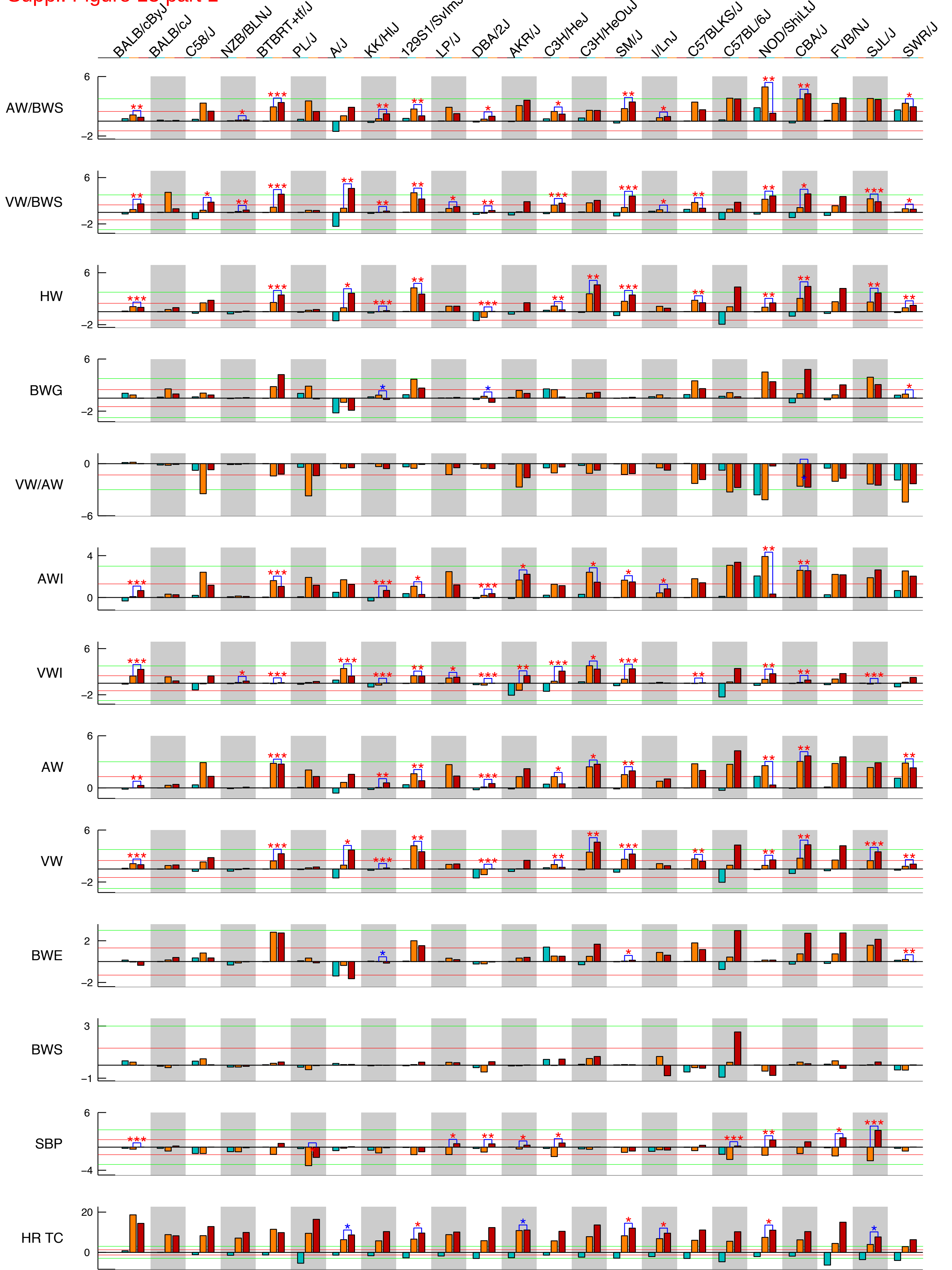

Suppl. Figure 13 part 2

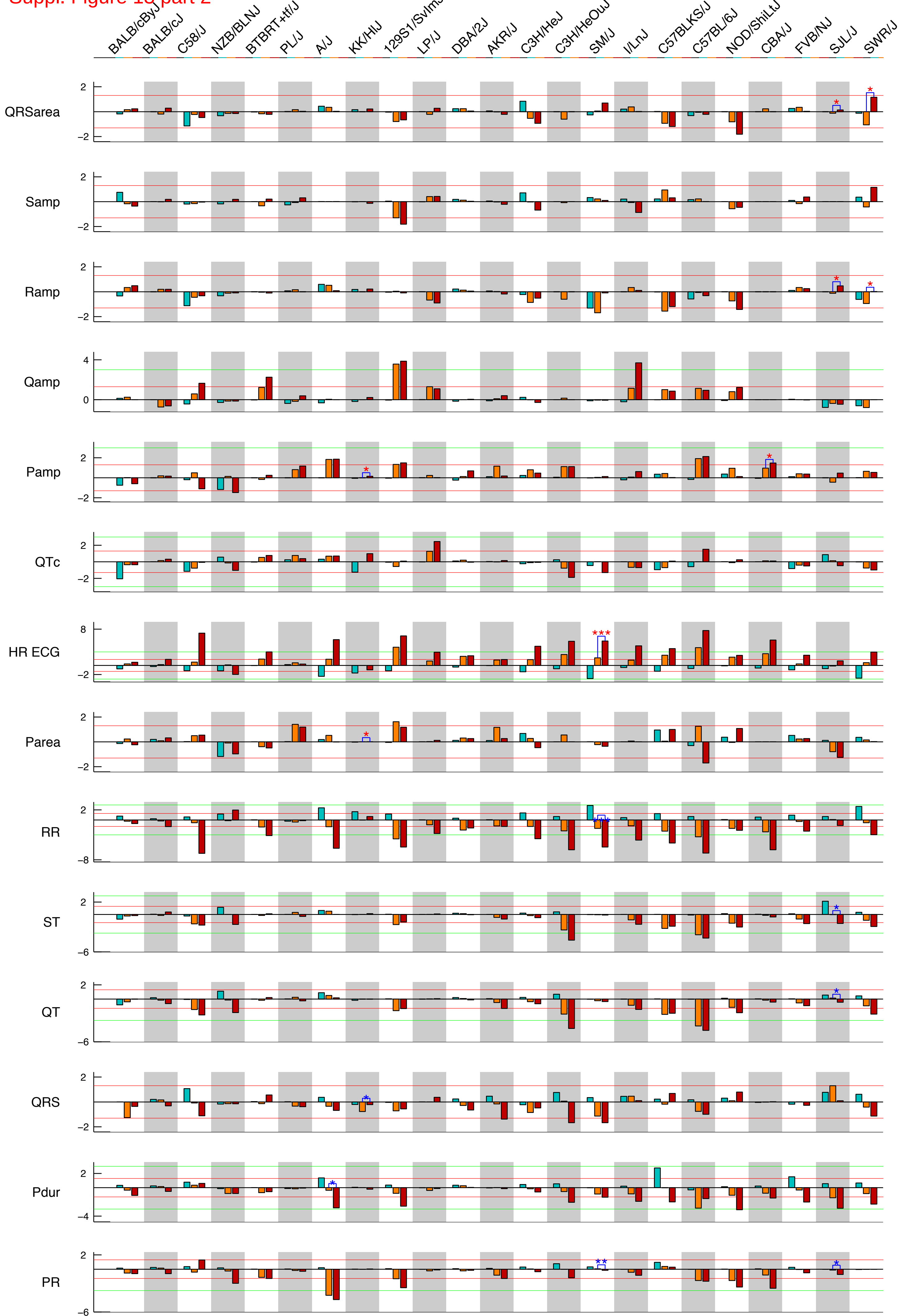

Suppl. Figure 14 part 1

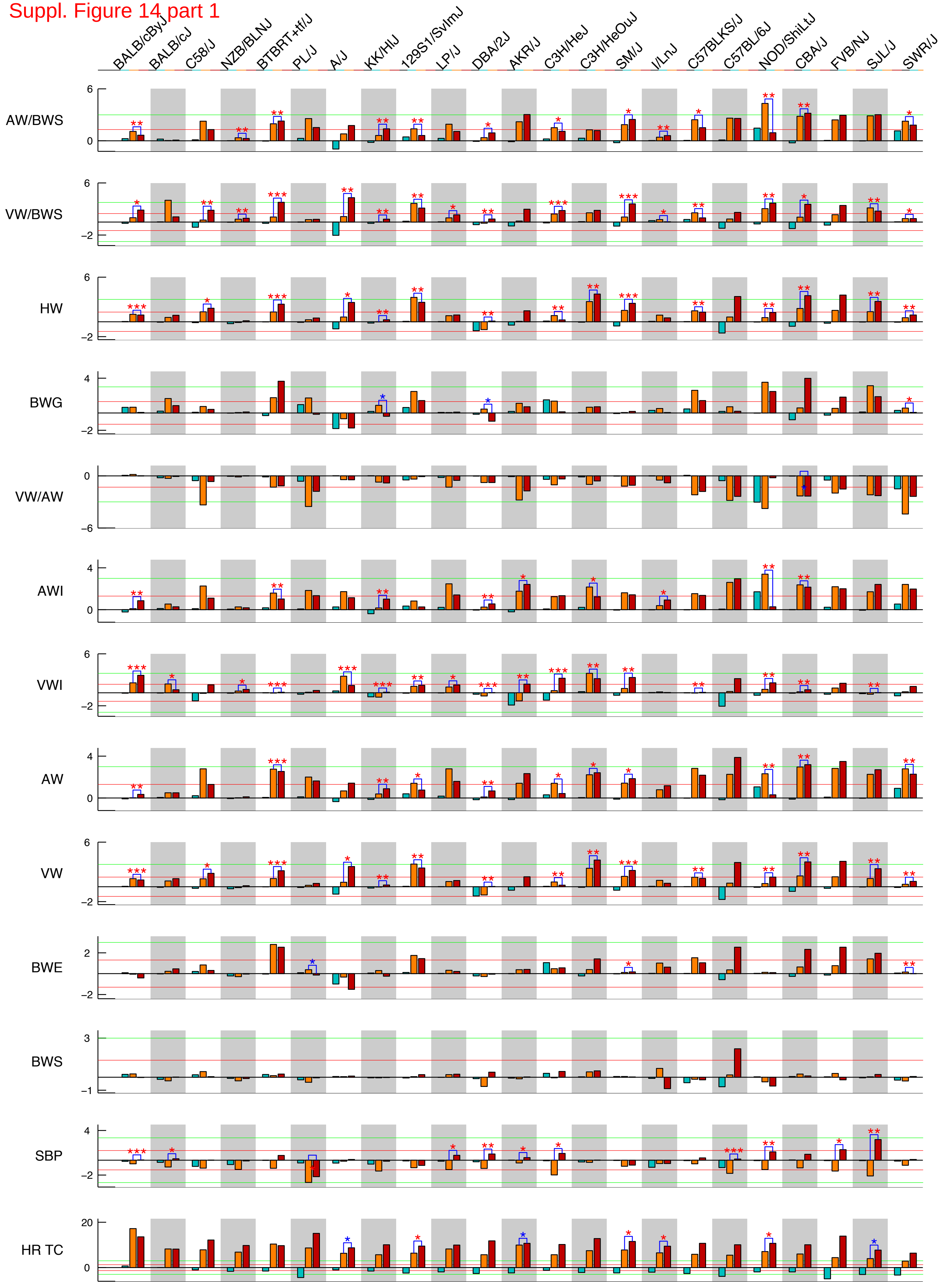

Suppl. Figure 14 part 2

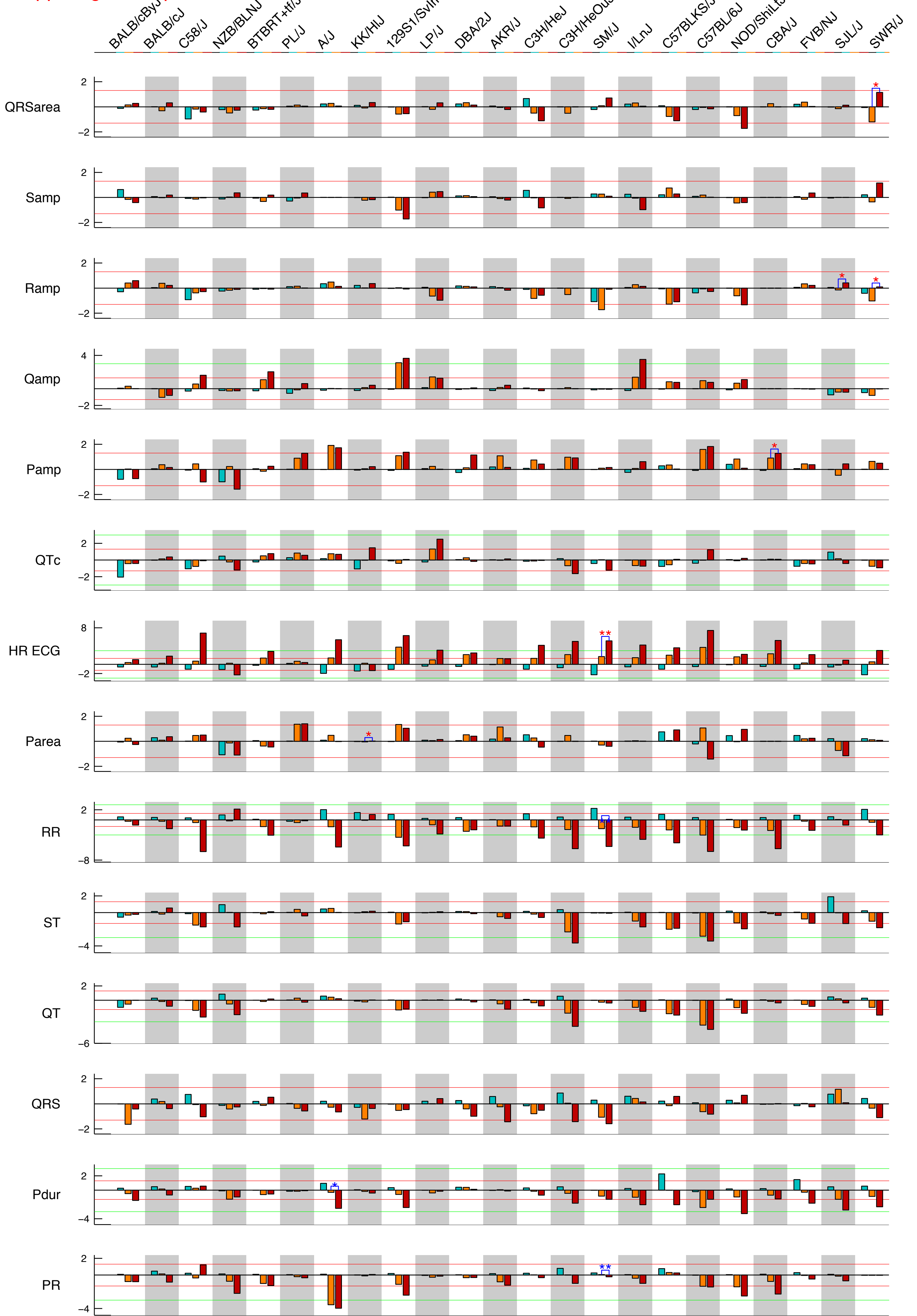

Suppl. Figure 15

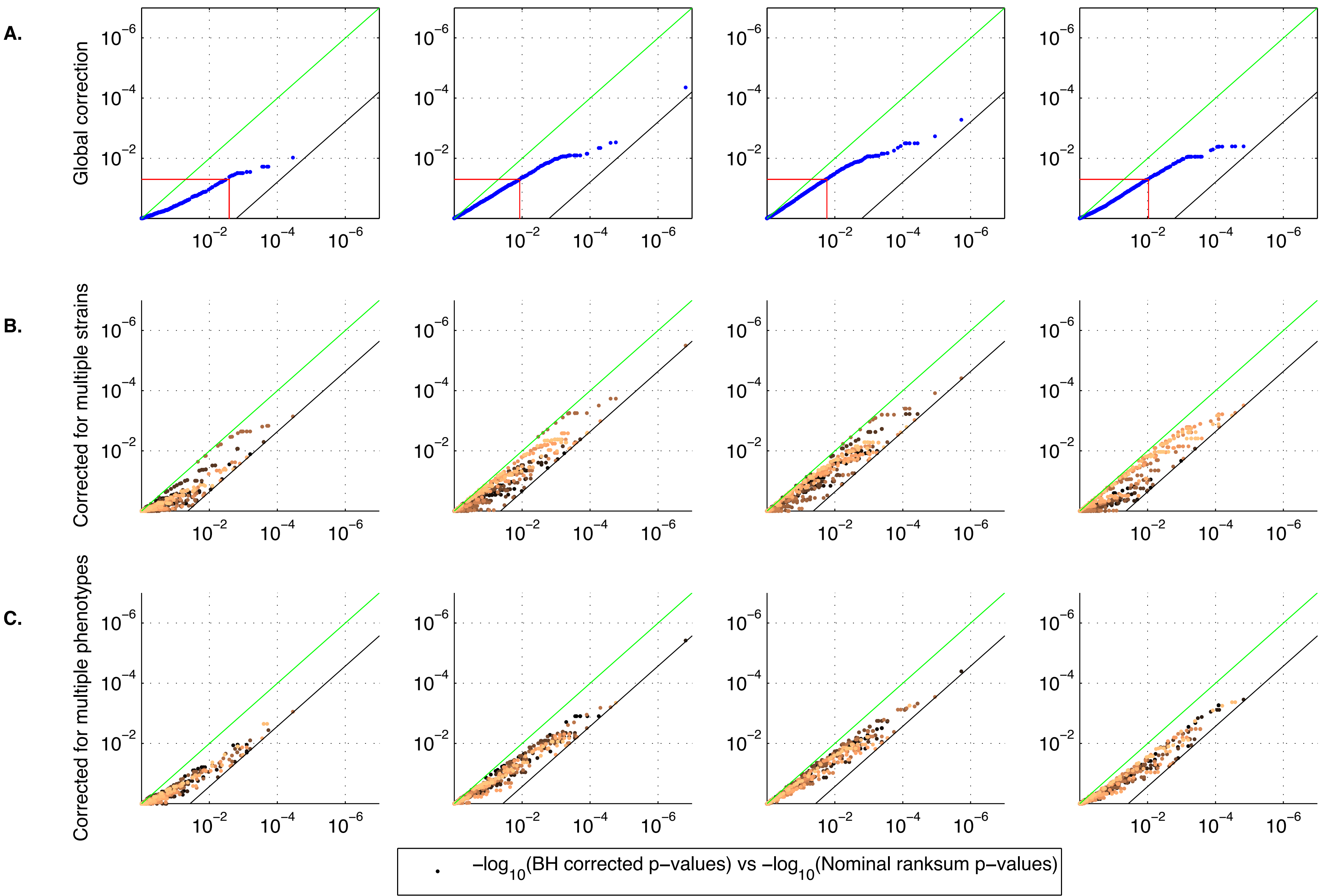

Suppl. Figure 16

# Number of significant observations with and without correction for multiple hypotheses

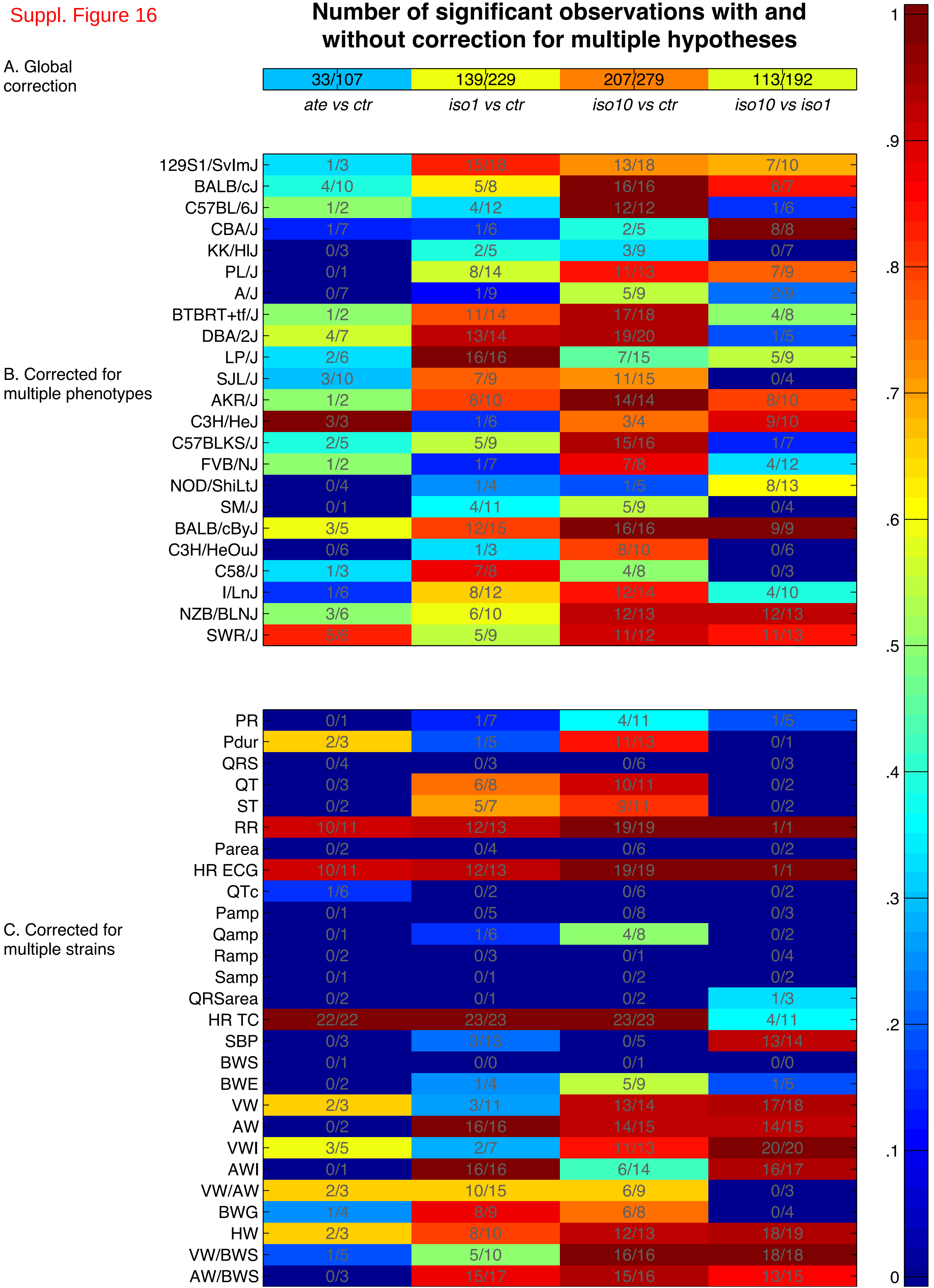

Suppl. Figure 17

# Number of significant observations with and without correction for multiple hypotheses

A. Global correction

| ate vs ctr | iso1 vs ctr | iso10 vs ctr | iso10 vs iso1 |
|---|---|---|---|
| 1/107 | 5/229 | 9/279 | 5/192 |

B. Corrected for multiple phenotypes

| | ate vs ctr | iso1 vs ctr | iso10 vs ctr | iso10 vs iso1 |
|---|---|---|---|---|
| 129S1/SvImJ | 1/3 | 8/18 | 5/18 | 6/10 |
| BALB/cJ | 3/10 | 3/8 | 6/16 | 2/7 |
| C57BL/6J | 1/2 | 2/12 | 3/12 | 1/6 |
| CBA/J | 1/7 | 1/6 | 2/5 | 6/8 |
| KK/HlJ | 0/3 | 2/5 | 1/9 | 0/7 |
| PL/J | 0/1 | 3/14 | 5/13 | 6/9 |
| A/J | 0/7 | 1/9 | 3/9 | 2/9 |
| BTBRT+tf/J | 1/2 | 4/14 | 7/18 | 2/8 |
| DBA/2J | 3/7 | 8/14 | 10/20 | 1/5 |
| LP/J | 2/6 | 3/16 | 3/15 | 2/9 |
| SJL/J | 2/10 | 4/9 | 4/15 | 0/4 |
| AKR/J | 0/2 | 4/10 | 6/14 | 3/10 |
| C3H/HeJ | 1/3 | 1/6 | 1/4 | 5/10 |
| C57BLKS/J | 1/5 | 5/9 | 7/16 | 1/7 |
| FVB/NJ | 1/2 | 1/7 | 4/8 | 0/12 |
| NOD/ShiLtJ | 0/4 | 1/4 | 1/5 | 7/13 |
| SM/J | 0/1 | 3/11 | 1/9 | 0/4 |
| BALB/cByJ | 2/5 | 5/15 | 2/16 | 7/9 |
| C3H/HeOuJ | 0/6 | 1/3 | 2/10 | 0/6 |
| C58/J | 1/3 | 4/8 | 1/8 | 0/3 |
| I/LnJ | 1/6 | 3/12 | 3/14 | 2/10 |
| NZB/BLNJ | 3/6 | 1/10 | 6/13 | 7/13 |
| SWR/J | 3/6 | 4/9 | 0/12 | 4/13 |

C. Corrected for multiple strains

| | ate vs ctr | iso1 vs ctr | iso10 vs ctr | iso10 vs iso1 |
|---|---|---|---|---|
| PR | 0/1 | 1/7 | 1/11 | 1/5 |
| Pdur | 1/3 | 1/5 | 4/13 | 0/1 |
| QRS | 0/4 | 0/3 | 0/6 | 0/3 |
| QT | 0/3 | 1/8 | 4/11 | 0/2 |
| ST | 0/2 | 1/7 | 2/11 | 0/2 |
| RR | 4/11 | 3/13 | 11/19 | 1/1 |
| Parea | 0/2 | 0/4 | 0/6 | 0/2 |
| HR ECG | 4/11 | 3/13 | 11/19 | 1/1 |
| QTc | 1/6 | 0/2 | 0/6 | 0/2 |
| Pamp | 0/1 | 0/5 | 0/8 | 0/3 |
| Qamp | 0/1 | 1/6 | 2/8 | 0/2 |
| Ramp | 0/2 | 0/3 | 0/1 | 0/4 |
| Samp | 0/1 | 0/1 | 0/2 | 0/2 |
| QRSarea | 0/2 | 0/1 | 0/2 | 1/3 |
| HR TC | 14/22 | 21/23 | 21/23 | 0/11 |
| SBP | 0/3 | 1/13 | 0/5 | 3/14 |
| BWS | 0/1 | 0/0 | 0/1 | 0/0 |
| BWE | 0/2 | 1/4 | 1/9 | 1/5 |
| VW | 2/3 | 2/11 | 4/14 | 10/18 |
| AW | 0/2 | 7/16 | 5/15 | 4/15 |
| VWI | 1/5 | 2/7 | 3/13 | 13/20 |
| AWI | 0/1 | 7/16 | 3/14 | 5/17 |
| VW/AW | 1/3 | 7/15 | 2/9 | 0/3 |
| BWG | 1/4 | 4/9 | 2/8 | 0/4 |
| HW | 0/3 | 2/10 | 5/13 | 10/19 |
| VW/BWS | 1/5 | 3/10 | 6/16 | 11/18 |
| AW/BWS | 0/3 | 10/17 | 7/16 | 4/15 |

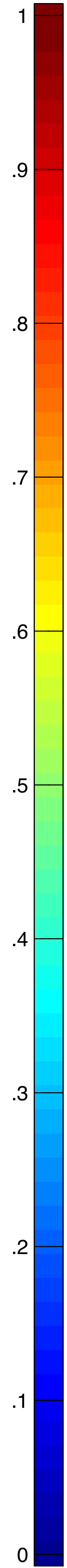

Supplement: Supplement S1 — Supplementary Results. (1.57 MB PDF) [file pone.0006610.s001.pdf]
